# Supernatants of *Bifidobacterium longum* and *Lactobacillus* *plantarum* Strains Exhibited Antioxidative Effects on A7R5 Cells

**DOI:** 10.3390/microorganisms9020452

**Published:** 2021-02-22

**Authors:** Yusheng Wang, Zhifeng Fang, Qixiao Zhai, Shumao Cui, Jianxin Zhao, Hao Zhang, Wei Chen, Wenwei Lu

**Affiliations:** 1 State Key Laboratory of Food Science and Technology, Jiangnan University, Wuxi 214122, China; 15061881533@163.com (Y.W.); zhifengf@foxmail.com (Z.F.); zhaiqixiao@jiangnan.edu.cn (Q.Z.); cuishumao@jiangnan.edu.cn (S.C.); jxzhao@jiangnan.edu.cn (J.Z.); zhanghao@jiangnan.edu.cn (H.Z.); weichen@jiangnan.edu.cn (W.C.); 2 School of Food Science and Technology, Jiangnan University, Wuxi 214122, China; 3 (Yangzhou) Institute of Food Biotechnology, Jiangnan University, Yangzhou 225004, China; 4 National Engineering Research Center for Functional Food, Jiangnan University, Wuxi 214122, China

**Keywords:** *Bifidobacterium*, *Lactobacillus*, antioxidant, transcriptome, cardiovascular diseases, protein biosynthesis

## Abstract

Vascular reactive oxygen species (ROS) play an essential role in cardiovascular diseases and the antioxidative effects of probiotics have been widely reported. To screen the probiotic strains that may prevent cardiovascular diseases, we tested the antioxidative effects of supernatants of different *Bifidobacterium* and *Lactobacillus* strains on A7R5 cells. Preincubation with supernatants of *B. longum* CCFM752, *L. plantarum* CCFM1149, or *L. plantarum* CCFM10 significantly suppressed the angiotensin II-induced increases in ROS levels and increased catalase (CAT) activity in A7R5, whereas CCFM752 inhibited NADPH oxidase activation and CCFM1149 enhanced the intracellular superoxide dismutase (SOD) activity simultaneously. Treatment with CCFM752, CCFM1149, or CCFM10 supernatants had no significant impact on transcriptional levels of *Cat*, *Sod1*, *Sod2*, *Nox1*, *p22phox*, or *p47phox*, but altered the overall transcriptomic profile and the expression of genes relevant to protein biosynthesis, and up-regulated the 60S ribosomal protein L7a (Rpl7a). A positive correlation between Rpl7a expression and intracellular CAT activity implied that Rpl7a may participate in CAT synthesis in A7R5. Supernatant of CCFM752 could also down-regulate the expression of NADPH oxidase activator 1 (Noxa1) and angiotensinogen in A7R5. Collectively, the probiotic strains CCFM752, CCFM1149, and CCFM10 exhibited antioxidative attributes on A7R5 cells and might help to reduce the risk of cardiovascular diseases.

## 1. Introduction

Reactive oxygen species (ROS) in blood vessel walls comprise an important risk factor for cardiovascular diseases such as hypertension [1,2] and atherosclerosis [2,3]. They function as signaling molecules in vascular walls, and are involved in basic physiological processes such as angiogenesis [4] and vasoconstriction [5]. However, ROS are likely to accumulate in vessels with insufficient reducing power or excessive stimulation by factors such as angiotensin II (Ang II) and aldosterone, which further inactivate the endothelium-derived nitrogen oxide (NO) and increase vascular tone [6]. Meanwhile, ROS might promote the proliferation and extracellular matrix secretion of vascular smooth muscle cells (VSMC) and result in vascular hypertrophy [7] and fibrosis [8]. Furthermore, vascular ROS can also activate T cell differentiation and up-regulate the synthesis of proinflammatory cytokines, such as IL-17A, TNF-α, and IFN-γ, thus accelerating the development of hypertension [9]. Vascular hydrogen peroxide (H_2_O_2_) can cause vascular inflammation and promote atherogenesis [3]. Therefore, scavenging excessive vascular ROS is considered helpful for the prevention of cardiovascular diseases.

Supplementation with antioxidants effectively removes ROS and helps to maintain the balance of oxide metabolism. Various diseases associated with oxidative stress, such as skin aging [10], have been treated and prevented using antioxidants, which have also been applied to treat hypertension [11,12], atherosclerosis [13], and vascular fibrosis [8]. Natural and synthetic antioxidants are available, but some synthetic types might trigger side effects [14]; thus, more natural antioxidants are needed. Microorganisms have recently emerged as a new source of natural antioxidants, and probiotics, such as *Lactobacillus* spp. and *Bifidobacterium* spp., can also help regulate the gut microbiome [15] and maintain gut epithelial integrity [16]. The antioxidative properties of *Lactobacillus plantarum* KSFY02 and *Bifidobacterium animalis* RH have recently been demonstrated in aged mice; both these strains were found to increase the activities of superoxide dismutase (SOD), catalase (CAT), and glutathione peroxidase (GSH-PX) in this model [17,18]. Probiotic metabolites can also inhibit increases in intracellular ROS in cell models [19,20]. Some probiotics can also up-regulate the intracellular SOD, CAT, and GSH-PX activities at the enzymatic or transcriptional level and prevent the cells from oxidative damage [20,21]. Hence, probiotic metabolites might enhance the activity of antioxidant enzymes and help to remove excessive ROS from hosts. Moreover, appropriate intake of probiotics regulates the balance of intestinal microflora and improves physiological metabolic status. Taken together, antioxidative probiotic supplements could be a new strategy with which to intervene in chronic diseases related to oxidative stress, including cardiovascular diseases.

The antioxidative effects of some probiotic strains have been tested in some cell lines, but seldom in vascular cells. This study aimed at evaluating the antioxidative effects of extracellular probiotic metabolites on VSMC in order to screen probiotic strains with the potential of preventing cardiovascular diseases. In this study the A7R5 cell line was incubated with the supernatants of *Bifidobacterium longum* or *Lactobacillus plantarum* strains, then stimulated with Ang II to trigger ROS generation. Intracellular ROS levels were determined to evaluate the antioxidative capacity of the probiotic strains. The effects of supernatants on intracellular total SOD (T-SOD), CAT, GSH-PX, and NADPH oxidase activities as well as the cell transcriptome were also investigated to assess the impact on the ROS-related pathways in A7R5.

## 2. Materials and Methods

### 2.1. Probiotic Strains, Culture Conditions, and Supernatant Preparation

The *B. longum* strains CCFM 666 and CCFM 752, and the *L. plantarum* strains CCFM1149, CCFM10, FGSYC22-5-L2 (L86) are preserved in Culture Collection of Food Microorganisms of Jiangnan University (Wuxi, China). Preservation and culture of microorganisms and supernatant preparation were conducted according to Xing et al. [19]. Briefly, probiotic strains were maintained at −80 °C in DeMan, Rogosa, and Sharpe (MRS) broth, supplemented with 30% (*v*/*v*) glycerol, and consecutively reactivated at least twice using 1% (*v*/*v*) inoculum in MRS broth, supplemented with 0.5 g/L L-cysteine, at 37 °C for 20–36 h under anaerobic conditions. For supernatant preparation, the *B. longum* and *L. plantarum* strains were cultured in l-cysteine-free MRS broth for 36 and 20 h, respectively, under anaerobic conditions, then separated by centrifugation at 6000× *g* for 10 min. Supernatants were neutralized with 1 M NaOH and then passed through filters (0.22 µm pore size) (Sangon Biotech (Shanghai) Co., Ltd., Shanghai, China) to remove microorganisms. The MRS broth used in this study was sterile and did not contain any antibiotics.

### 2.2. Cell Culture

A7R5 cells (National Collection of Authenticated Cell Cultures, Shanghai, China) were cultured in 75-cm^2^ flasks, containing Dulbecco’s modified Eagle’s medium (DMEM) (Gibco, NY, USA) supplemented with 10% fetal bovine serum (FBS) (Gibco), 100 U/mL penicillin, and 100 mg/mL streptomycin (both from Shanghai Bolight Biotechnology Co. Ltd., Shanghai, China), at 37 °C under a 5% CO_2_ atmosphere. The media were renewed every three days and the cells were sub-cultured when they reached 70–80% confluence.

### 2.3. Intracellular ROS Measurement

A7R5 cells (2 × 10^4^/well) seeded in 24-well plates were incubated for 48 h, then the medium was replaced with DMEM containing 0.1% FBS for 24 h. Thereafter, quiescent cells were incubated with supernatants or MRS broth (control, model, and positive control; pH 7.0) at a ratio of 3% (*v*/*v*) for 12 h. Cells in the control, the model, the positive control, and supernatant intervention groups were stimulated with 10^−7^ mol/L Ang II (MedChemExpress, Shanghai, China) for 4 h, and the control cells were incubated with phosphate buffered saline (PBS) (3% *v*/*v*). The positive control was incubated with Tiron (10 mm) for 1 h before Ang II stimulation to scavenge superoxide anions (O_2_^−^) or with 2 mM GSH for 90 min to scavenge H_2_O_2_. The intracellular ROS (O_2_^−^ and H_2_O_2_) were measured using dihydroethidium (DHE) or 2′,7′-dichlorodihydrofluorescein diacetate (DCFH-DA) as previously described by Niu et al. [22] and Zafari et al. [23], respectively. Briefly, the cells were washed twice with Hank’s balanced salt solution, then incubated with DMEM containing 10 μM DHE or 5 μM DCFH-DA (both from Beyotime, Shanghai, China) for 30 min at 37 °C under a 5% CO_2_ atmosphere. The fluorescence probe was washed twice with Hank’s balanced salt solution and the cells were photographed using an inverted fluorescence microscope (Nikon, Tokyo, Japan). Relative intracellular ROS levels were calculated as relative fluorescence density on the images using Image Pro Plus version 6.0 (Media Cybernetics Inc., Bethesda, MD, USA).

### 2.4. Cell Viability Assays

Confluent cells in 96-well plates were quiesced for 24 h, then incubated with DMEM, MRS broth, or supernatants (3% *v*/*v*) for 12 h. The media were replaced with DMEM (100 μL), containing 5.0 g/L methylthiazolyldiphenyl-tetrazolium bromide (MTT) (Beyotime, Shanghai, China), and the plates were incubated for 4 h. Thereafter, 150 μL DMSO was added to the wells, the plates were shaken, and the optical density (OD)_570_ was determined using a microplate spectrophotometer (Thermo Fisher Scientific Inc., Waltham, MA, USA).

### 2.5. Intracellular Enzyme Activity Assays

Confluent cells in 6-cm plates were quiesced for 24 h, then incubated with MRS broth (control and model; pH 7.0) or supernatants at a ratio of 3% *v*/*v* for 12 h and treated with Ang II or PBS for 4 h. Thereafter, cells were washed five times with ice-cold PBS, and then transferred to 15 mL centrifuge tubes and washed twice with ice-cold PBS, and resuspended in 1 mL of PBS containing MS-SAFE 50X (Beyotime), a protease inhibitor cocktail for general use. The mixture was ultrasonicated on ice, then CAT, T-SOD, and GSH-PX activities were determined in the sonicate using assay kits (Institute of Biological Engineering of Nanjing Jiancheng, Nanjing, China) as described by the manufacturer. Protein concentrations in the sonicates were determined using bicinchoninic acid (BCA) protein assay kits (Beyotime). NADPH activity was determined using luminescence assays as described by Griendling et al. [24].

### 2.6. Total RNA Extraction and RT-qPCR

Confluent cells in 6-cm plates used for RT-qPCR analysis were quiesced, incubated with MRS broth or supernatants, and stimulated with Ang II according to Section 2.5. Total RNA was extracted from cells using the FastPure^®®^ Cell/Tissue total RNA isolation kits (Vazyme, Nanjing, China) as described by the manufacturer. The concentration was determined using a Nanodrop 2000 spectrophotometer (Thermo Fisher Inc., Waltham, MA, USA). Single-strand cDNA was synthesized from total RNA using the HiScript^®®^ III-RT SuperMix for qPCR (+gDNA wiper) (Vazyme) according to the protocol provided. Quantitative real-time PCR (qPCR) was conducted as described in the protocol provided with iTaq™ Universal SYBR^®®^ Green Supermix (Bio-Rad Laboratories, Inc., Shanghai, China), in a CFX Connect^TM^ Real-Time System (Bio-Rad Laboratories, Inc.). Real-time PCR data were analyzed using the 2^−ΔΔCt^ method and *GAPDH* was used as the reference gene. Table S1 lists the qPCR primers.

### 2.7. Cell Transcriptome Sequencing and Annotation

Confluent cells in 10-cm plates were divided into 5 groups, containing the control, the model, CCFM752, CCFM1149, and CCFM10, and were quiesced, incubated with MRS broth or supernatants, and stimulated with Ang II according to Section 2.5. Total RNA was extracted using the FastPure^®®^ Cell/Tissue total RNA isolation kit (Vazyme), and the concentration and integrity of RNA were confirmed using the Nanodrop 2000 (Thermo Fisher Inc.) and an Agilent 2100 Bioanalyzer system (Agilent Technologies Inc., Santa Clara, CA, USA), respectively. We synthesized cDNA using the HiScript^®®^ III 1st Strand cDNA synthesis kit (+gDNA wiper) (Vazyme) and verified it using Agilent 2000 (Agilent Technologies Inc.). A cDNA library was sequenced on an Illumina HiSeq2500 sequencing platform (Illumina Inc., San Diego, CA, USA). The cDNA sequences were annotated according to the NR, Swiss-Prot, Pfam, EggNOG, GO, and KEGG databases.

### 2.8. Data Processing and Statistical Analysis

Experimental data were processed and analyzed using GraphPad Prism version 6.01 (GraphPad Software Inc., San Diego, CA, USA). Significant differences between the model and other groups were verified using one-way ANOVA with Dunnett multiple comparison tests without repeated measures. Data are shown as means ± SEM. Values with *p* < 0.05 were considered significantly different. For cell transcriptomic analysis, differentially expressed genes (DEG) were analyzed using DESeq2, DEGseq, and edgeR, and the results were considered significantly different at *p* < 0.05. Correlations between relative gene expression and intracellular enzyme activity were analyzed using GraphPad Prism version 6.01.

## 3. Results

### 3.1. Probiotic Supernatants Inhibited Ang II-Induced ROS Increases in A7R5 Cells

**Figure 1:**
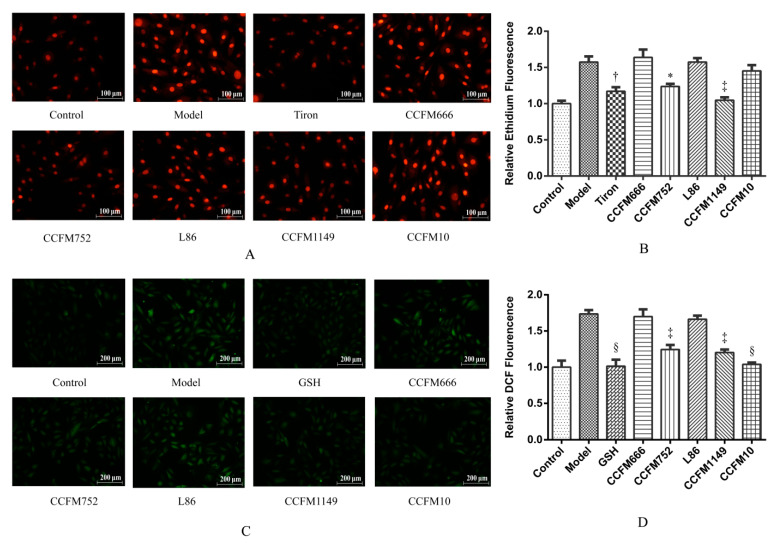
Relative intracellular reactive oxygen species (ROS) levels in different groups of A7R5 cells. (**A**) Photographs of ethidium fluorescence of A7R5 cells. (**B**) Relative intracellular O_2_^−^ levels of A7R5 cells. (**C**) Photographs of (dichlorofluorescein) (DCF) fluorescence of A7R5 cells. (**D**) Relative intracellular H_2_O_2_ levels of A7R5 cells. Values are shown as means ± SEM (n = 3). * *p* < 0.05, † *p* < 0.01, ‡ *p* < 0.001, and § *p* < 0.0001, each probiotic supernatant intervention group compared with the model group.

Different groups of A7R5 cells, except the control group, were stimulated with Ang II, and ROS levels were evaluated to determine the antioxidative effects of probiotic strains. Ang II increased the intracellular O_2_^−^ and H_2_O_2_ levels 1.57-and 1.74-fold, respectively, (Figure 1B,D). The supernatants of *B. longum* CCFM752, and *L. plantarum* CCFM1149 and CCFM10 significantly inhibited the Ang II-induced increase in intracellular H_2_O_2_ levels at rates of 66.6% ± 15.0%, 72.3% ± 10.2%, and 94.6% ± 5.9%, respectively (Figure 1B). The CCFM752 and CCFM1149 supernatants also similarly suppressed the Ang II-induced increases in intracellular O_2_^−^ at rates of 58.4% ± 11.4% and 91.7% ± 12.3%, respectively (Figure 1B). The supernatants of CCFM666 and L86 did not decrease the intracellular ROS levels in A7R5 (Figure 1B,D). 

### 3.2. Probiotic Supernatants Increased the Intracellular Antioxidative Enzyme Activities and Inhibited NADPH Oxidase Activation

**Figure 2:**
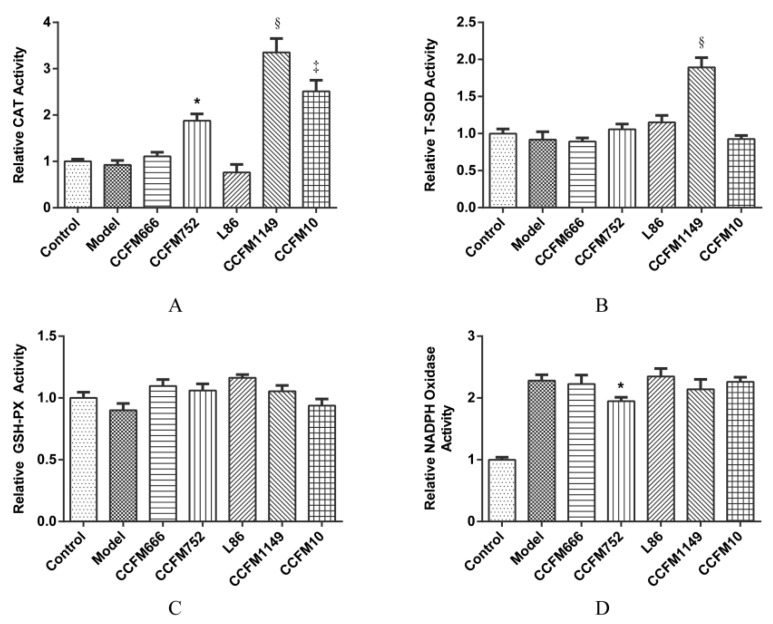
Activity of intracellular enzymes in different groups of A7R5 cells. (**A**) Intracellular catalase (CAT) activity. (**B**) Intracellular total super oxide dismutase (T-SOD) activity. (**C**) Intracellular glutathione peroxidase (GSH-PX) activity. (**D**) Intracellular NADPH oxidase activity. Values are mean ± SEM (n = 3). * *p* < 0.05, ‡ *p* < 0.001, and § *p* < 0.0001, each probiotic supernatant intervention group compared with the model group.

To verify the effects of probiotic supernatants on ROS-related enzymes in A7R5 cells, we assessed the activities of intracellular T-SOD, CAT, GSH-PX, and NADPH oxidase in cells from different groups. Figure 2A shows that CCFM752, CCFM1149, and CCFM10 supernatants increased CAT activity in A7R5 cells 1.8-, 3.3-, and 2.25-fold, respectively, compared to that in the control or the model group. Figure 2B shows that the CCFM1149 supernatant significantly increased intracellular T-SOD activity by ~1.8-fold compared to that in the control or the model group. In contrast, the CCFM666 and L86 supernatants did not affect the activities of SOD and CAT in A7R5 (Figure 2A,B). None of the supernatants significantly altered intracellular GSH-PX activity (Figure 2C). 

Ang II can activate NADPH oxidase which is one of the major sources of vascular ROS [1]. We therefore determined the effects of supernatants by measuring intracellular NADPH oxidase activity using a chemiluminescence method. Figure 2D shows that Ang II significantly increased the intracellular NADPH oxidase activity 2.3-fold compared to that in the control group. The CCFM752 supernatant slightly, but significantly inhibited NADPH oxidase activation by 25.9% ± 5.0% (*p* = 0.01), whereas the other supernatants did not significantly affect NADPH oxidase activity.

### 3.3. Probiotic Supernatants Did Not Alter Transcriptional Levels of Intracellular Enzymes

**Figure 3:**
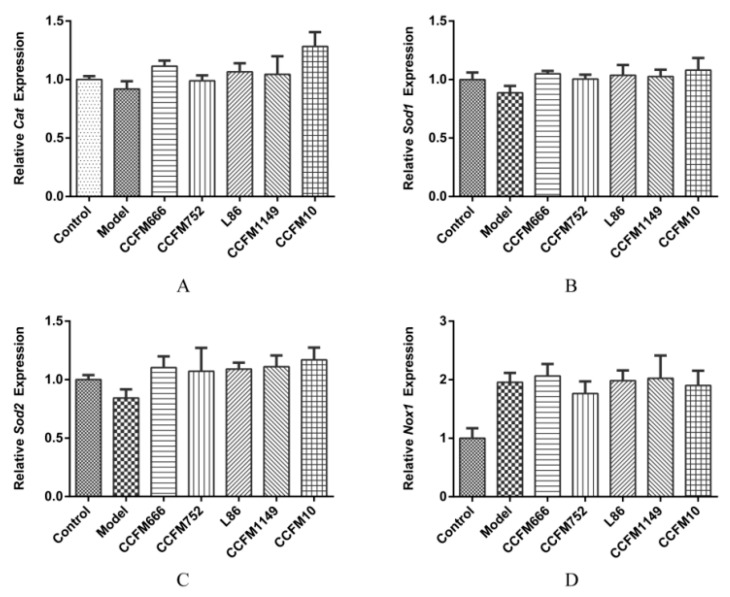
Relative transcriptional levels of genes in A7R5 cells. (**A**) Transcriptional levels of *Cat*. (**B**) Transcriptional levels of *Sod1*. (**C**) Transcriptional levels of *Sod2*. (**D**) Transcriptional levels of *Nox1*. Values are shown as means ± SEM (n = 3).

We determined *Cat*, *Sod1,* and *Sod2* expression using RT-qPCR to verify whether the supernatants affected CAT and SOD at the transcriptional level in A7R5 cells. Figure 3A shows that CCFM666, CCFM752, L86, CCFM1149, and CCFM10 supernatants did not affect intracellular *Cat* expression compared to that in the model group. None of the probiotic supernatants increased intracellular *Sod1* and *Sod2* expression (Figure 3B,C). We then determined the effects of supernatants on the expression of NADPH oxidase-related genes, *Nox1*, *p22phox,* and *p47 phox.* Figure 3D shows that intracellular *Nox1* expression almost doubled after Ang II stimulation, and that the CCFM752 supernatant tended to decrease *Nox1* expression but did not reach statistical significance. No other probiotic supernatants influenced *Nox1* (Figure 3D) or intracellular *p22phox* and *p47phox* expression (Figure S4).

### 3.4. Probiotic Supernatants Altered Transcriptome of A7R5 Cells

**Figure 4:**
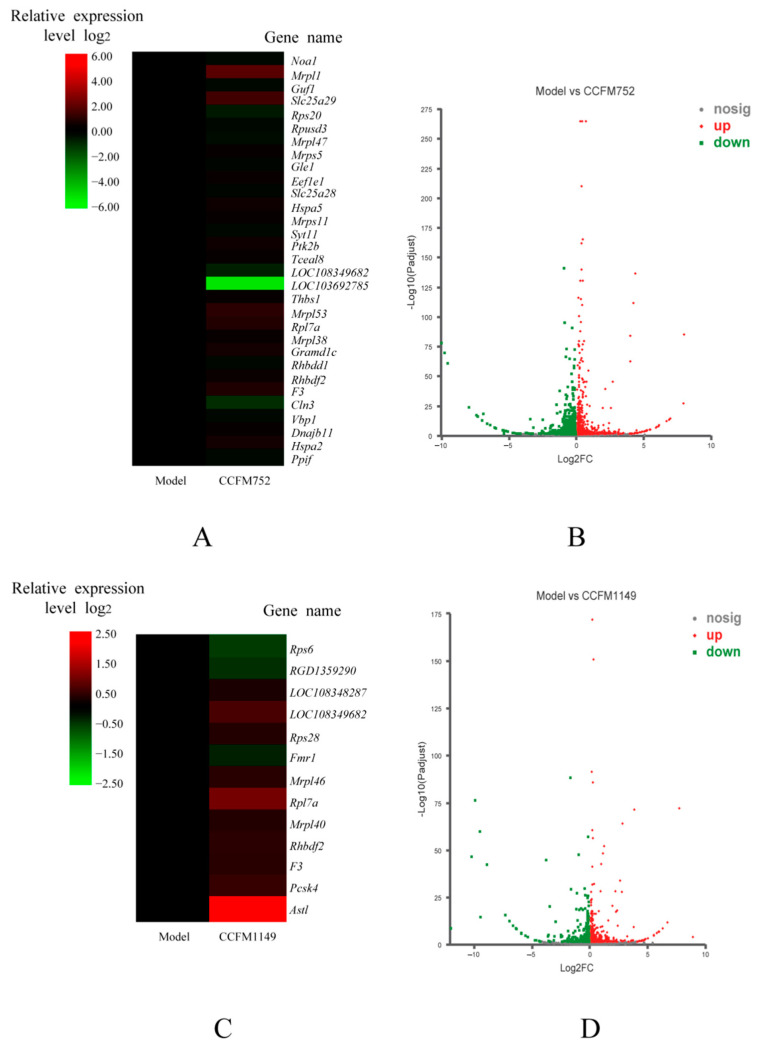

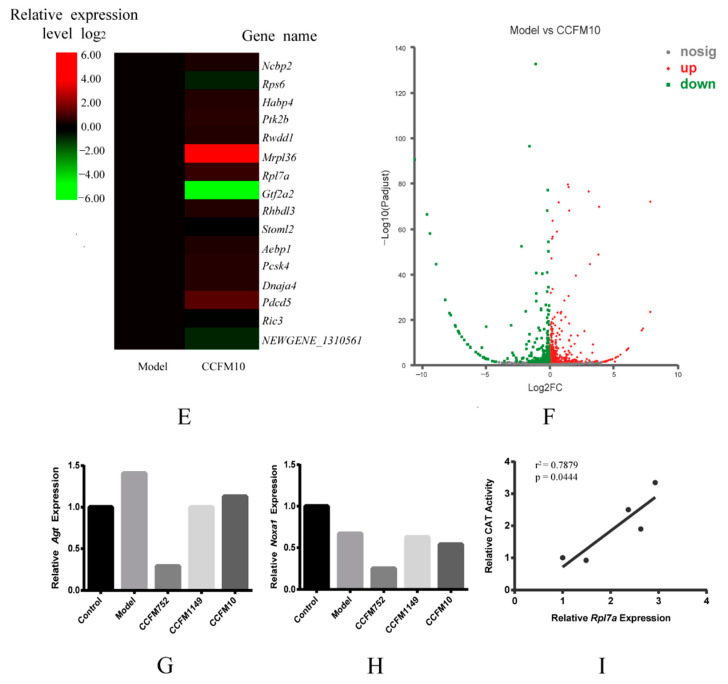
Transcriptional profiles and relative gene expression levels of A7R5 cells. (**A**) Heatmap of differentially expressed genes (DEG) relevant to protein biosynthesis between the model and CCFM752 group. (**B**) Volcano plot of transcriptomic differences between the model and CCFM752 group. (**C**) Heatmap of DEG relevant to protein biosynthesis between the model and CCFM1149 group. (**D**) Volcano plot of transcriptomic differences between the model and CCFM1149 group. (**E**) Heatmap of DEG relevant to protein biosynthesis between the model and CCFM10 group. (**F**) Volcano plot of transcriptomic differences between the model and CCFM10 group. (**G**) Relative expression of angiotensinogen in different groups. (**H**) Relative expression of NADPH oxidase activator 1 (Noxa1) in different groups. (**I**) Linear regression analysis of intracellular CAT activity and ribosomal protein L7a (Rpl7a) expression in different groups.

We then explored how CCFM752, CCFM1149, and CCFM10 supernatants increased the intracellular antioxidant capacity, by analyzing cell transcriptomes. Volcano plots showed significant changes in the transcriptomes of cells incubated with CCFM752, CCFM1149, and CCFM10 supernatants compared to those in the model group, and that the transcriptome could be clearly distinguished (Figure 4B,D,F). We found 803 DEG in the CCFM752, compared to those in the model group, among which 328 and 475 genes were significantly up-regulated and down-regulated, respectively (Table S2). We found 554 DEG in the CCFM1149, compared to those in the model group, including 275 and 279 up-regulated and down-regulated genes, respectively (Table S3). The CCFM10 group had 474 DEG compared to those in the model group, of which 215 and 259 were significantly up-regulated and down-regulated, respectively (Table S4). Gene ontology functional annotation revealed that CCFM752 altered the expression of genes related to protein synthesis, including ribosomal proteins such as Rpl7a (ribosomal protein L7a), LOC108349682 (60S ribosomal protein L35a), LOC103692785 (60S ribosomal protein L39), Rps20 (ribosomal protein S20), and mitochondrial ribosomal proteins such as Mrpl1 (mitochondrial ribosomal protein L1), Mrpl47 (mitochondrial ribosomal protein L47), and Mrpl53 (mitochondrial ribosomal protein L53) (Figure 4A). The expression of thirteen protein-synthesis-related genes was altered in the CCFM1149 group, compared to that in the model group, including Rps6 (ribosomal protein S6), LOC108348287 (60S ribosomal L36a), LOC108349682, Rps28 (ribosomal protein S28), Mrpl46, Rpl7a, and Mrpl40 (ribosomal protein L40) (Figure 4C). Compared to that in the model group, CCFM10 altered the expression of sixteen genes related to protein synthesis, including Rps6, Mrpl36 (mitochondrial ribosomal protein L36), and Rpl7a (Figure 4E). Among these DEG, the expression of Rpl7a was up-regulated, and the activity of intracellular CAT was also increased to various degrees by the three supernatants, compared to those in the model and control groups, after these interventions. The results of linear correlation analyses revealed a significant positive correlation between intracellular Rpl7a expression and CAT enzyme activity (r^2^ = 0.7879, *p* = 0.0444; Figure 4I). Among the other DEG, we found that in CCFM752 group the expression of Noxa1 (NADPH oxidase activator 1) was decreased 0.38-fold (Figure 4H), whereas that of angiotensinogen (Agt) was decreased 0.21-fold, compared to those in the model group (Figure 4G).

## 4. Discussion

Vascular ROS participate in signaling pathways associated with hypertension [1,2], atherosclerosis [2,3], vascular hypertrophy [7], and vascular fibrosis [8]. Some probiotic bacterial strains that have antioxidative functions in animal and cell models contribute to reducing ROS [17,18,19,20]. Therefore, we tested the antioxidative effects of metabolites of various probiotic strains on A7R5 cells to screen antioxidative ones that might be beneficial in preventing cardiovascular diseases. Preincubation with the supernatants of *Bifidobacterium longum* CCFM752 and *L. plantarum* CCFM1149 prevented Ang II induced O_2_^−^ and H_2_O_2_ increase in A7R5 cells, whereas *L. plantarum* CCFM10 prevented only the increase in H_2_O_2_. We also showed that the original components of MRS broth did not affect this induced ROS increase (Figure S2). These findings suggested that these probiotic strains exert antioxidative effects on VSMC and might block the ROS-induced pathological pathways in cardiovascular diseases. The supernatants of *B. longum* CCFM666 and *L. plantarum* L86 did not suppress the induced ROS increase in A7R5 cells, indicating that the antioxidative properties of CCFM752, CCFM1149, and CCFM10 were strain-specific. The detrimental effects that the supernatants might exert on cell viability were also excluded by MTT assay (Figure S1). Hence, we speculated that the CCFM752, CCFM1149, and CCFM10 supernatants probably altered intracellular pathways related to ROS metabolism.

We next assessed the intracellular T-SOD, CAT, and GSH-PX levels in A7R5 cells to determine the impact of supernatants of three probiotic strains on cellular antioxidative enzyme activities. The CCFM1149 supernatant increased the intracellular T-SOD activity in A7R5 cells, which helped to detoxify O_2_^−^, an important risk factor for hypertension [25]. The activity of CAT was higher in the cells incubated with CCFM752, CCFM1149, and CCFM10 supernatants than in the control and model cells, which might explain why the intracellular H_2_O_2_ levels were decreased in these groups. This implied that these strains might help to block the H_2_O_2_-involved pathological pathways in vascular hypertrophy [7], vascular inflammation [3], as well as vascular calcification [3]. None of the supernatants altered the intracellular GSH-PX activity of A7R5 cells, suggesting that they probably did not alter the intracellular ROS levels through affecting GSH-PX activity. The activation of NADPH oxidase, a vital ROS generator in the vascular wall, is related to hypertension [1,2] and atherosclerosis [2,3]. Since Ang II is a potent agonist of NADPH oxidase in VSMC [24], we assessed whether the supernatants could affect NADPH oxidase activation. The CCFM752 supernatant slightly inhibited the intracellular NADPH oxidase activation induced by Ang II, which could inhibit the generation of vascular ROS. The effects of MRS broth on intracellular T-SOD, CAT, GSH-PX, and NADPH oxidase activities were all excluded (Figure S3). These results showed that probiotic CCFM752, CCFM1149, and CCFM10 metabolites could enhance antioxidative capacity of A7R5 cells in different aspects.

The RT-qPCR findings showed that none of the supernatants significantly changed the relative *Cat*, *Sod1*, *Sod2*, *Nox1*, *p22phox,* and *p47phox* gene expression in A7R5 cells. This implied that the three probiotics did not affect SOD and CAT at the transcriptional level, and that the CCFM752 supernatant might affect other factors involved in the regulation of NADPH oxidase activity. 

We then investigated overall intracellular pathway changes caused by probiotic supernatants, by analyzing the transcriptomes, to identify potential mechanisms behind these phenomena. The transcriptional profiles differed between the model, and the CCFM752, CCFM1149, and CCFM10 groups. The expression of *Noxa1* was lower in the CCFM752 group than in the model group. Noxa1 is an activator of vascular NADPH oxidase and participates in ROS generation in VSMC and endothelial cells [22,26]. This helps to explain why intracellular NADPH oxidase activity was relatively lower in the CCFM752 group. The expression of genes related to translation (including ribosomal and mitochondrial ribosomal proteins), protein processing, and folding was altered in the CCFM752, CCFM1149, and CCFM10 groups, compared to that in the model group, which indicated the altered protein biosynthesis state in these three groups. Ribosomal proteins are the essential structural units of ribosomes and can impact ribosome biogenesis, thus regulating the cell proteome and affecting cell phenotypes [27]. The activities of CAT and SOD can be regulated at the translational level [28,29]. Therefore, we speculated that the enhanced intracellular SOD and/or CAT activity in the CCFM752, CCFM1149, and CCFM10 groups could be attributed to the translational upregulation of enzymatic proteins. Among these differentially expressed ribosomal proteins, Rpl7a was up-regulated in the CCFM752, CCFM1149, and CCFM10 groups. Rpl7a is involved in the 60S ribosome, the expression of which is linked to ribosome functions [30]. Although its specific functions remain indistinct, Rpl7a had been proven to be overexpressed in plants and animals under stress [31,32]. The overexpression of Rpl7a in A7R5 cells might be partly due to the complexity of probiotic metabolites, which might have imposed some stress on the cells. Since the activity of CAT was much higher in the CCFM752, CCFM1149, and CCFM10 groups than in the control and model groups, we conducted linear regression analysis which showed that these two factors were well positively correlated. This implied that Rpl7a may participate in CAT biosynthesis; however, further investigation is needed to elucidate the mechanisms. We also found down-regulated Agt expression in the CCFM752 group. In mammals, the liver and vascular walls mainly synthesize Agt, which is converted to Ang II via the catalysis of renin and angiotensin I converting enzyme (ACE) in vivo [6]. Thus, CCFM752 also has the potential to down-regulate Agt expression and reduce the risk of Ang II-related cardiovascular diseases [33].

## 5. Conclusions

The supernatants of *B. longum* CCFM752, *L. plantarum* CCFM1149, and *L. plantarum* CCFM10 significantly inhibited the increase in ROS induced by Ang II in A7R5 cells without altering cell viability. The supernatants of these three strains enhanced intracellular CAT activity, while CCFM752 suppressed NADPH oxidase activation and CCFM1149 increased intracellular SOD activity in A7R5 cells, all of which contributed to an increased intracellular antioxidative capacity. The increased intracellular CAT and SOD levels after incubation with the supernatants were probably not associated with a transcriptional increase in *Cat*, *Sod1*, or *Sod2*, and the inhibition of NADPH oxidase might not be associated with transcriptional changes in Nox1, p22phox, and p47phox. The CCFM752 supernatant decreased the expression of Noxa1, an activator of NADPH oxidase, which might explain the inhibition of NADPH oxidase activation. The supernatants of three strains altered the expression of genes related to protein synthesis in A7R5, and the up-regulated Rpl7a in these groups was positively correlated with intracellular CAT activity. The CCFM752 supernatant down-regulated Agt expression, which might help to lower the risk of hypertension and atherosclerosis. In summary, the metabolites of probiotic strains CCFM752, CCFM1149, and CCFM10 exert antioxidative effects on A7R5 through different intracellular pathways. Thus, these probiotic strains have the potential of preventing cardiovascular diseases. Further studies are needed to testify their effects on animal models and human beings, and their physiological properties, such as antibiotic resistance, virulence, as well as the dose-effect relationships, should also be examined before the industrialization of these probiotic strains. 

## 6. Patents

Wenwei Lu, Wei Chen, Yusheng Wang, et al. A *Lactobacillus plantarum* strain that can reduce risk factors for hypertension and its application [P]. Chinese patent, 2020110607514.

Wei Chen, Wenwei Lu, Yusheng Wang, et al. A *Bifidobacterium longum* strain that can reduce the level of reactive oxygen species in vascular smooth muscle cells [P]. Chinese patent, 2020110649201.

## Data Availability

Raw transcriptome data of A7R5 cells in the control, the model, CCFM752, CCFM1149, and CCFM10 groups have been deposited in the National Center for Biotechnology Information Search database (NCBI) under BioProject accession code PRJNA694834.

## Supplementary Materials

The following are available online at https://www.mdpi.com/2076-2607/9/2/452/s1, Figure S1: Influence of MRS broth and probiotic supernatants preincubation on cell viability of A7R5 cells, Table S1: Oligonucleotide primer pairs used for qPCR, Figure S2: Influence of MRS broth preincubation and angiotensin II stimulation on intracellular ROS levels of A7R5 cells, Table S2: Differentially expressed genes between CCFM752 group and the model group of A7R5 cells, Figure S3: Influence of MRS broth on intracellular enzyme activity in A7R5 cells, Table S3: Differentially expressed genes between CCFM1149 group and the model group of A7R5 cells, Figure S4: Relative transcriptional levels of *p22phox* and *p47phox* of A7R5 cells, Table S4: Differentially expressed genes between CCFM10 group and the model group of A7R5 cells.
