# Supplementary material for: Supernatants of Bifidobacterium longum and Lactobacillus plantarum Strains Exhibited Antioxidative Effects on A7R5 Cells
Source: Microorganisms. 2021 Feb 22;9(2):452. doi: 10.3390/microorganisms9020452 (PMC7927071; doi:10.3390/microorganisms9020452)
Supplement: Supplementary file 1 [file microorganisms-09-00452-s001.zip › Supplementary Information/Supplementary Materials.docx]

**Supplementary Materials**

**Table S1**. Oligonucleotide primer pairs used for qPCR

| Genes | Primer sequences | |
| --- | --- | --- |
| *Sod1* | F | 5′-TTCGAGCAGAAGGCAAGCGGTGAA |
|  | R | 5′-AATCCCAATCACACCACAAGCCAA |
| *Sod2* | F | 5′-ATTAACGCGCAGATCATGCAG |
|  | R | 5′-TTTCAGATAGTCAGGTCTGACGTT |
| *Cat* | F | 5′-CGACCGAGGGATTCCAGATG |
|  | R | 5′-ATCCGGGTCTTCCTGTGCAA |
| *Nox1* | F | 5′-CTGCTCTCCTTCCTGAGGGGCACCTGCT |
|  | R | 5′-GACAATCCCCCCCAGGCCATGGATCCCTA |
| *p22phox* | F | 5'-GCGGTGTGGACAGAAGTACC |
|  | R | 5'-CTTGGGTTTAGGCTCAATGG |
| *p47phox* | F | 5'-CCCAGCGACAGATTAGAAGC |
|  | R | 5'-TGGATTGTCCTTTGAGTCAGG |
| *GAPDH* | F | 5'-ACCCAGAAGACTGTGGATGG |
|  | R | 5'-TTCAGCTCAGGGATGACCTT |

**Table S2**. Differentially expressed genes between CCFM752 group and the model group of A7R5 cells

| Gene_id | Gene name | FC | Log_2_FC | P value | Padj. |
| --- | --- | --- | --- | --- | --- |
| ENSRNOG00000000129 | *Phf24* | 1.808 | 0.854198 | 0.000121 | 0.001774 |
| ENSRNOG00000000307 | *Mical1* | 1.214 | 0.27997 | 7.35E-05 | 0.001146 |
| ENSRNOG00000000413 | *Pln* | 1.574 | 0.654223 | 0.000111 | 0.001643 |
| ENSRNOG00000000464 | *Rxrb* | 0.809 | -0.30509 | 0.000799 | 0.008621 |
| ENSRNOG00000000476 | *Zbtb22* | 0.83 | -0.26939 | 0.002516 | 0.021257 |
| ENSRNOG00000000479 | *Kifc1* | 0.772 | -0.37268 | 0.000232 | 0.003061 |
| ENSRNOG00000000500 | *Scube3* | 2.238 | 1.162526 | 0.000189 | 0.002575 |
| ENSRNOG00000000522 | *Cpne5* | 3.852 | 1.945428 | 0.000744 | 0.008124 |
| ENSRNOG00000000561 | *Pald1* | 0.809 | -0.3063 | 0.006493 | 0.044458 |
| ENSRNOG00000000585 | *Amd1* | 1.207 | 0.271466 | 0.001778 | 0.016361 |
| ENSRNOG00000000598 | *Tube1* | 0.57 | -0.81216 | 0.000297 | 0.003779 |
| ENSRNOG00000000763 | *RT1-M3-1* | 1.297 | 0.375411 | 0.000542 | 0.00629 |
| ENSRNOG00000000809 | *Atat1* | 0.8 | -0.32239 | 0.000749 | 0.008151 |
| ENSRNOG00000000818 | *Nrm* | 0.346 | -1.53112 | 0.000858 | 0.009099 |
| ENSRNOG00000000827 | *Ier3* | 1.294 | 0.372227 | 5.01E-16 | 3.91E-14 |
| ENSRNOG00000000983 | *Zfp394* | 0.777 | -0.36418 | 0.003975 | 0.030367 |
| ENSRNOG00000001079 | *Daglb* | 1.213 | 0.278512 | 0.002046 | 0.018186 |
| ENSRNOG00000001160 | *NEWGENE_1586233* | 0.813 | -0.29877 | 0.001487 | 0.014178 |
| ENSRNOG00000001214 | *Pfkl* | 0.83 | -0.26827 | 1.95E-08 | 6.67E-07 |
| ENSRNOG00000001245 | *Pcbp3* | 1.285 | 0.361546 | 6.38E-11 | 2.98E-09 |
| ENSRNOG00000001276 | *Pcnt* | 0.816 | -0.29257 | 0.000838 | 0.00893 |
| ENSRNOG00000001304 | *Bcr* | 1.298 | 0.375994 | 3.32E-06 | 7.32E-05 |
| ENSRNOG00000001414 | *Serpine1* | 1.669 | 0.739022 | 1.2E-268 | 3.4E-265 |
| ENSRNOG00000001441 | *Tmem120a* | 0.821 | -0.28395 | 0.00016 | 0.002238 |
| ENSRNOG00000001482 | *Gtf2ird2* | 0.797 | -0.32825 | 0.002683 | 0.022256 |
| ENSRNOG00000001500 | *Rab4b* | 0.812 | -0.29965 | 1.05E-05 | 0.000206 |
| ENSRNOG00000001501 | *Snrpa* | 1.442 | 0.527648 | 9.82E-08 | 3.01E-06 |
| ENSRNOG00000001531 | *Chac2* | 0.6 | -0.73773 | 0.002807 | 0.023158 |
| ENSRNOG00000001607 | *Adamts1* | 1.496 | 0.580829 | 2.57E-83 | 1.65E-80 |
| ENSRNOG00000001699 | *Setd4* | 0.827 | -0.2746 | 0.002681 | 0.022257 |
| ENSRNOG00000001714 | *Atp13a4* | 1.856 | 0.891878 | 7.17E-05 | 0.001128 |
| ENSRNOG00000001764 | *Vps8* | 0.792 | -0.33554 | 7.91E-05 | 0.001226 |
| ENSRNOG00000001803 | *Dnajb11* | 1.214 | 0.280145 | 5.32E-14 | 3.48E-12 |
| ENSRNOG00000001845 | *Top3b* | 0.815 | -0.29522 | 0.00709 | 0.047653 |
| ENSRNOG00000001928 | *Il1rap* | 1.345 | 0.427852 | 0.001543 | 0.014596 |
| ENSRNOG00000001956 | *Dzip3* | 0.717 | -0.48065 | 0.000871 | 0.009223 |
| ENSRNOG00000001963 | *Mx2* | 0.62 | -0.69063 | 0.004094 | 0.031096 |
| ENSRNOG00000001979 | *Rcan1* | 1.252 | 0.323861 | 9.6E-06 | 0.000192 |
| ENSRNOG00000002055 | *Noa1* | 0.792 | -0.33656 | 0.000984 | 0.010134 |
| ENSRNOG00000002070 | *Mrpl1* | 3.521 | 1.815918 | 0.000583 | 0.006678 |
| ENSRNOG00000002089 | *Ccng2* | 0.76 | -0.39514 | 1.27E-07 | 3.78E-06 |
| ENSRNOG00000002105 | *Cdc7* | 0.605 | -0.72436 | 3.77E-05 | 0.000641 |
| ENSRNOG00000002207 | *Guf1* | 0.796 | -0.32892 | 0.002415 | 0.020565 |
| ENSRNOG00000002215 | *Mylk* | 1.452 | 0.538023 | 1.3E-10 | 5.94E-09 |
| ENSRNOG00000002218 | *Stbd1* | 1.492 | 0.577613 | 2.25E-06 | 5.27E-05 |
| ENSRNOG00000002294 | *Snx29* | 0.822 | -0.28201 | 0.000391 | 0.004781 |
| ENSRNOG00000002331 | *Aldh3a1* | 0.768 | -0.38066 | 1.43E-07 | 4.21E-06 |
| ENSRNOG00000002342 | *Aldh3a2* | 0.805 | -0.31232 | 2.71E-05 | 0.000479 |
| ENSRNOG00000002418 | *Tgfb2* | 1.219 | 0.286122 | 3.86E-11 | 1.87E-09 |
| ENSRNOG00000002471 | *Polq* | 0.626 | -0.67528 | 0.004413 | 0.033004 |
| ENSRNOG00000002537 | *Wnk3* | 1.369 | 0.45281 | 6E-05 | 0.000965 |
| ENSRNOG00000002542 | *Heatr6* | 0.792 | -0.33695 | 0.002413 | 0.02059 |
| ENSRNOG00000002610 | *Carhsp1* | 0.816 | -0.29331 | 3.36E-05 | 0.00058 |
| ENSRNOG00000002652 | *Rap1gap2* | 1.215 | 0.281162 | 0.003471 | 0.02737 |
| ENSRNOG00000002667 | *Lamc2* | 1.466 | 0.552035 | 2.03E-39 | 4.29E-37 |
| ENSRNOG00000002693 | *Nme1* | 1.201 | 0.264696 | 2.82E-07 | 7.79E-06 |
| ENSRNOG00000002708 | *Phf8* | 0.828 | -0.27268 | 0.000512 | 0.005986 |
| ENSRNOG00000002711 | *Nuf2* | 0.653 | -0.61585 | 1.67E-05 | 0.00031 |
| ENSRNOG00000002754 | *Areg* | 3.703 | 1.888844 | 0.001184 | 0.01182 |
| ENSRNOG00000002848 | *Maoa* | 0.797 | -0.32759 | 5.38E-05 | 0.000868 |
| ENSRNOG00000002932 | *Wdr19* | 0.823 | -0.28152 | 0.007469 | 0.049481 |
| ENSRNOG00000002936 | *Golt1a* | 1.823 | 0.866476 | 0.001489 | 0.014189 |
| ENSRNOG00000002946 | *Socs3* | 0.64 | -0.64494 | 1.03E-43 | 2.53E-41 |
| ENSRNOG00000002956 | *Stim2* | 0.804 | -0.31415 | 9.3E-05 | 0.001407 |
| ENSRNOG00000003086 | *Cenpv* | 1.314 | 0.394083 | 0.006495 | 0.044455 |
| ENSRNOG00000003105 | *Kif19* | 2.621 | 1.39036 | 0.00035 | 0.004345 |
| ENSRNOG00000003139 | *Smc1a* | 0.829 | -0.26972 | 5.49E-07 | 1.44E-05 |
| ENSRNOG00000003224 | *Nudt16l1* | 0.006 | -7.43964 | 9.43E-20 | 9.91E-18 |
| ENSRNOG00000003259 | *C1qtnf1* | 0.808 | -0.30718 | 4.34E-05 | 0.000722 |
| ENSRNOG00000003284 | *Epn3* | 1.905 | 0.929486 | 0.0011 | 0.011116 |
| ENSRNOG00000003338 | *Pmp22* | 1.244 | 0.315377 | 0.002414 | 0.020573 |
| ENSRNOG00000003346 | *Fancb* | 0.426 | -1.23059 | 1.77E-05 | 0.000327 |
| ENSRNOG00000003388 | *Cenpf* | 0.491 | -1.02669 | 0.00038 | 0.00467 |
| ENSRNOG00000003442 | *Adora1* | 2.755 | 1.462219 | 0.002614 | 0.021927 |
| ENSRNOG00000003549 | *Cnpy2* | 0.717 | -0.47939 | 2.55E-16 | 2.05E-14 |
| ENSRNOG00000003581 | *Pou2f1* | 0.54 | -0.88828 | 0.006001 | 0.041832 |
| ENSRNOG00000003597 | *Tuba4a* | 1.275 | 0.350361 | 1.86E-05 | 0.00034 |
| ENSRNOG00000003669 | *Myocd* | 1.314 | 0.39382 | 2.62E-15 | 1.88E-13 |
| ENSRNOG00000003712 | *Ppp4r3b* | 0.794 | -0.33275 | 2.01E-05 | 0.000363 |
| ENSRNOG00000003790 | *Mael* | 3.174 | 1.666452 | 0.002946 | 0.024025 |
| ENSRNOG00000003870 | *C1qtnf2* | 0.747 | -0.42123 | 0.005859 | 0.041115 |
| ENSRNOG00000003872 | *Slc40a1* | 0.442 | -1.17804 | 0.003503 | 0.027572 |
| ENSRNOG00000003881 | *Nit1* | 0.816 | -0.29355 | 1.07E-12 | 6.13E-11 |
| ENSRNOG00000004003 | *Dusp10* | 1.469 | 0.555091 | 0.000181 | 0.002488 |
| ENSRNOG00000004100 | *Trib1* | 1.263 | 0.337014 | 3.84E-09 | 1.46E-07 |
| ENSRNOG00000004135 | *RGD1311745* | 1.481 | 0.566916 | 0.000741 | 0.008108 |
| ENSRNOG00000004169 | *Fzr1* | 0.774 | -0.3693 | 1.36E-07 | 4.03E-06 |
| ENSRNOG00000004201 | *Rims2* | 0.743 | -0.4285 | 0.00134 | 0.013112 |
| ENSRNOG00000004208 | *Crim1* | 1.36 | 0.443892 | 1.4E-113 | 1.3E-110 |
| ENSRNOG00000004276 | *Itga3* | 1.25 | 0.32138 | 1.91E-91 | 1.39E-88 |
| ENSRNOG00000004298 | *Dpys* | 7.11 | 2.82995 | 0.000961 | 0.00994 |
| ENSRNOG00000004317 | *Vipr2* | 2.084 | 1.059432 | 0.000534 | 0.006202 |
| ENSRNOG00000004351 | *Slc25a29* | 2.598 | 1.377438 | 0.001445 | 0.013873 |
| ENSRNOG00000004377 | *Lpin1* | 0.718 | -0.47812 | 1.23E-15 | 9.1E-14 |
| ENSRNOG00000004403 | *Slc25a32* | 0.743 | -0.42929 | 0.000447 | 0.005385 |
| ENSRNOG00000004420 | *Rad21* | 0.824 | -0.27918 | 1.78E-09 | 7.06E-08 |
| ENSRNOG00000004479 | *Aurka* | 0.792 | -0.33673 | 1.89E-07 | 5.43E-06 |
| ENSRNOG00000004484 | *Commd5* | 0.761 | -0.3942 | 0.003299 | 0.026336 |
| ENSRNOG00000004682 | *Parpbp* | 0.467 | -1.09699 | 1.29E-08 | 4.53E-07 |
| ENSRNOG00000004733 | *Wdr25* | 0.583 | -0.77947 | 0.000175 | 0.002416 |
| ENSRNOG00000004757 | *Tmem158* | 0.825 | -0.27722 | 0.005473 | 0.038864 |
| ENSRNOG00000004874 | *Flrt3* | 0.729 | -0.45592 | 3.49E-07 | 9.44E-06 |
| ENSRNOG00000004921 | *Nusap1* | 0.631 | -0.66485 | 1.39E-11 | 7.04E-10 |
| ENSRNOG00000005008 | *Angpt4* | 1.252 | 0.324561 | 1.65E-09 | 6.57E-08 |
| ENSRNOG00000005037 | *Kif18a* | 0.464 | -1.10686 | 9.11E-08 | 2.8E-06 |
| ENSRNOG00000005096 | *Bzw2* | 1.207 | 0.271147 | 1.49E-07 | 4.38E-06 |
| ENSRNOG00000005101 | *Mia2* | 0.774 | -0.36906 | 2.96E-06 | 6.68E-05 |
| ENSRNOG00000005151 | *Dync2li1* | 0.673 | -0.57186 | 0.000431 | 0.005224 |
| ENSRNOG00000005287 | *Syne3* | 0.781 | -0.35631 | 0.00068 | 0.007548 |
| ENSRNOG00000005357 | *Ctc1* | 0.772 | -0.37247 | 3.51E-05 | 0.0006 |
| ENSRNOG00000005376 | *Mad2l1* | 0.725 | -0.46456 | 9.77E-06 | 0.000194 |
| ENSRNOG00000005659 | *Aurkb* | 0.649 | -0.62377 | 3.81E-08 | 1.25E-06 |
| ENSRNOG00000005765 | *Ap4s1* | 0.612 | -0.70921 | 0.002244 | 0.019523 |
| ENSRNOG00000005769 | *Smg8* | 0.816 | -0.29414 | 0.000967 | 0.009988 |
| ENSRNOG00000005872 | *Tcf7* | 1.868 | 0.901504 | 4.31E-05 | 0.000718 |
| ENSRNOG00000005936 | *Foxm1* | 0.591 | -0.75835 | 1.47E-14 | 9.91E-13 |
| ENSRNOG00000005960 | *RGD1311744* | 0.579 | -0.78743 | 5.51E-05 | 0.000889 |
| ENSRNOG00000005996 | *Lhx6* | 1.373 | 0.457438 | 0.00178 | 0.016369 |
| ENSRNOG00000006060 | *Matn2* | 2.271 | 1.183102 | 0.000132 | 0.001896 |
| ENSRNOG00000006086 | *Lynx1* | 4.733 | 2.242889 | 0.003734 | 0.028991 |
| ENSRNOG00000006103 | *Tbc1d31* | 0.785 | -0.34992 | 0.005975 | 0.041681 |
| ENSRNOG00000006198 | *Prr11* | 0.513 | -0.96419 | 1.42E-07 | 4.2E-06 |
| ENSRNOG00000006236 | *Dsn1* | 0.779 | -0.3609 | 4.13E-05 | 0.000693 |
| ENSRNOG00000006340 | *Memo1* | 1.247 | 0.318617 | 1.01E-06 | 2.55E-05 |
| ENSRNOG00000006403 | *Nectin1* | 1.217 | 0.283022 | 6.9E-09 | 2.56E-07 |
| ENSRNOG00000006469 | *Cdk2* | 0.728 | -0.45792 | 0.000254 | 0.003311 |
| ENSRNOG00000006472 | *Hspa2* | 1.439 | 0.525471 | 0.001994 | 0.017826 |
| ENSRNOG00000006541 | *Nup107* | 0.81 | -0.3042 | 0.001519 | 0.014435 |
| ENSRNOG00000006619 | *Dnajc9* | 0.795 | -0.33034 | 0.000518 | 0.006046 |
| ENSRNOG00000006684 | *Zfp317* | 0.808 | -0.30818 | 0.001547 | 0.014618 |
| ENSRNOG00000006700 | *Wdyhv1* | 0.561 | -0.83445 | 0.000827 | 0.008834 |
| ENSRNOG00000006731 | *Spc25* | 0.562 | -0.83218 | 1.26E-05 | 0.000241 |
| ENSRNOG00000006740 | *Castor1* | 0.61 | -0.7122 | 0.003761 | 0.029148 |
| ENSRNOG00000006763 | *Rbm18* | 1.236 | 0.305109 | 7.05E-09 | 2.61E-07 |
| ENSRNOG00000006827 | *Tmem198b* | 0.725 | -0.4645 | 0.00048 | 0.005703 |
| ENSRNOG00000006877 | *Efnb1* | 0.806 | -0.31141 | 3.59E-12 | 1.94E-10 |
| ENSRNOG00000006931 | *Eepd1* | 0.813 | -0.29866 | 0.001916 | 0.017315 |
| ENSRNOG00000007002 | *Lif* | 1.336 | 0.418367 | 1E-07 | 3.06E-06 |
| ENSRNOG00000007078 | *Ccn4* | 1.273 | 0.348066 | 8.46E-32 | 1.52E-29 |
| ENSRNOG00000007147 | *Cyp46a1* | 0.762 | -0.39168 | 0.001013 | 0.010372 |
| ENSRNOG00000007175 | *Mier1* | 0.81 | -0.30338 | 0.000132 | 0.001897 |
| ENSRNOG00000007206 | *LOC361016* | 0.435 | -1.20247 | 0.000937 | 0.00975 |
| ENSRNOG00000007221 | *Dut* | 0.738 | -0.43829 | 5.17E-05 | 0.000839 |
| ENSRNOG00000007284 | *Slc2a1* | 1.448 | 0.534495 | 0.000163 | 0.002274 |
| ENSRNOG00000007324 | *Plxna2* | 4.296 | 2.102969 | 0.000179 | 0.002468 |
| ENSRNOG00000007338 | *Fbln2* | 1.207 | 0.270924 | 2.33E-08 | 7.78E-07 |
| ENSRNOG00000007345 | *Amot* | 0.787 | -0.34552 | 8E-11 | 3.72E-09 |
| ENSRNOG00000007367 | *Sept4* | 1.612 | 0.68896 | 2.32E-06 | 5.41E-05 |
| ENSRNOG00000007390 | *Nfkbia* | 1.991 | 0.99322 | 6.06E-28 | 9.29E-26 |
| ENSRNOG00000007478 | *Cry2* | 0.817 | -0.29187 | 0.000126 | 0.001828 |
| ENSRNOG00000007483 | *Ccnf* | 0.6 | -0.73588 | 5.11E-13 | 3.04E-11 |
| ENSRNOG00000007529 | *Bmf* | 0.819 | -0.28826 | 2.79E-06 | 6.32E-05 |
| ENSRNOG00000007576 | *Zfp597* | 0.747 | -0.42159 | 0.002979 | 0.02427 |
| ENSRNOG00000007657 | *Col27a1* | 1.47 | 0.55619 | 8.2E-05 | 0.001262 |
| ENSRNOG00000007660 | *Fntb* | 1.316 | 0.396115 | 0.002095 | 0.018509 |
| ENSRNOG00000007726 | *Mcam* | 1.338 | 0.419787 | 1.39E-28 | 2.17E-26 |
| ENSRNOG00000007865 | *Ephb1* | 9.777 | 3.289382 | 0.003296 | 0.026353 |
| ENSRNOG00000007894 | *Mpdz* | 0.816 | -0.29389 | 3.85E-05 | 0.00065 |
| ENSRNOG00000007906 | *Bub1b* | 0.584 | -0.77505 | 5.61E-24 | 7.27E-22 |
| ENSRNOG00000007925 | *Pak6* | 1.758 | 0.813831 | 0.001493 | 0.014212 |
| ENSRNOG00000008012 | *Abcb4* | 1.267 | 0.341165 | 7.23E-06 | 0.000148 |
| ENSRNOG00000008040 | *Pimreg* | 0.575 | -0.79808 | 6.37E-15 | 4.45E-13 |
| ENSRNOG00000008057 | *Krt7* | 1.41 | 0.495715 | 1.27E-65 | 4.84E-63 |
| ENSRNOG00000008101 | *Tmem251* | 1.378 | 0.462808 | 0.00145 | 0.013916 |
| ENSRNOG00000008115 | *Arhgap11a* | 0.704 | -0.50644 | 1.58E-11 | 7.97E-10 |
| ENSRNOG00000008159 | *Msantd3* | 1.247 | 0.318698 | 0.00737 | 0.049 |
| ENSRNOG00000008170 | *Jph2* | 1.271 | 0.345667 | 7.28E-16 | 5.55E-14 |
| ENSRNOG00000008244 | *LOC690035* | 0.83 | -0.26818 | 0.006604 | 0.045054 |
| ENSRNOG00000008256 | *Mrpl38* | 1.257 | 0.329638 | 0.002181 | 0.019095 |
| ENSRNOG00000008264 | *Fndc8* | 5.777 | 2.53039 | 0.005063 | 0.036767 |
| ENSRNOG00000008336 | *Tnfrsf11b* | 1.614 | 0.690547 | 7.07E-12 | 3.74E-10 |
| ENSRNOG00000008351 | *RGD1308117* | 2.22 | 1.150715 | 2.89E-05 | 0.000507 |
| ENSRNOG00000008353 | *Iqch* | 0.575 | -0.79822 | 0.00254 | 0.021411 |
| ENSRNOG00000008409 | *Myo1f* | 1.568 | 0.648504 | 0.000121 | 0.001766 |
| ENSRNOG00000008450 | *LOC100359539* | 0.681 | -0.55526 | 1.31E-28 | 2.06E-26 |
| ENSRNOG00000008555 | *Rps20* | 0.614 | -0.70257 | 0.001753 | 0.016167 |
| ENSRNOG00000008569 | *Ndufa6* | 0.793 | -0.33394 | 1.39E-05 | 0.000262 |
| ENSRNOG00000008580 | *Nbn* | 0.758 | -0.39978 | 0.000183 | 0.00251 |
| ENSRNOG00000008595 | *Ttc12* | 0.662 | -0.59493 | 0.007273 | 0.048529 |
| ENSRNOG00000008609 | *Capn3* | 0.401 | -1.31891 | 0.000153 | 0.002152 |
| ENSRNOG00000008618 | *Tex10* | 0.77 | -0.37629 | 5.04E-05 | 0.000825 |
| ENSRNOG00000008620 | *Smad3* | 0.822 | -0.28304 | 1.4E-09 | 5.64E-08 |
| ENSRNOG00000008637 | *Strada* | 0.717 | -0.47938 | 4.46E-07 | 1.19E-05 |
| ENSRNOG00000008642 | *Snx11* | 0.756 | -0.40428 | 3.61E-07 | 9.72E-06 |
| ENSRNOG00000008652 | *Phip* | 0.791 | -0.33768 | 0.001443 | 0.013879 |
| ENSRNOG00000008678 | *Antxr1* | 0.822 | -0.28359 | 7.35E-09 | 2.72E-07 |
| ENSRNOG00000008846 | *Plag1* | 0.798 | -0.32629 | 0.004017 | 0.030648 |
| ENSRNOG00000008862 | *Abcg4* | 1.211 | 0.276352 | 0.005198 | 0.037551 |
| ENSRNOG00000008950 | *Adi1* | 0.768 | -0.38101 | 0.000226 | 0.002989 |
| ENSRNOG00000009084 | *Rpusd3* | 0.794 | -0.33267 | 0.001637 | 0.015278 |
| ENSRNOG00000009116 | *Itgb3bp* | 0.629 | -0.66962 | 0.006374 | 0.04379 |
| ENSRNOG00000009144 | *Lad1* | 2.555 | 1.353512 | 2.28E-05 | 0.00041 |
| ENSRNOG00000009149 | *Jam3* | 1.284 | 0.361207 | 1.08E-07 | 3.25E-06 |
| ENSRNOG00000009173 | *Smad6* | 0.809 | -0.30603 | 0.00048 | 0.005705 |
| ENSRNOG00000009198 | *Rab6b* | 1.26 | 0.333002 | 9.7E-06 | 0.000193 |
| ENSRNOG00000009222 | *Epha2* | 1.251 | 0.323289 | 5.21E-09 | 1.96E-07 |
| ENSRNOG00000009276 | *Schip1* | 1.231 | 0.299563 | 6.39E-05 | 0.001021 |
| ENSRNOG00000009278 | *Ift88* | 0.648 | -0.62581 | 0.004532 | 0.033686 |
| ENSRNOG00000009339 | *Cenpe* | 0.571 | -0.80838 | 2.1E-21 | 2.42E-19 |
| ENSRNOG00000009503 | *Depdc1* | 0.588 | -0.76566 | 7.79E-05 | 0.001211 |
| ENSRNOG00000009536 | *Pgp* | 0.815 | -0.29599 | 9.76E-06 | 0.000194 |
| ENSRNOG00000009538 | *Etfdh* | 0.817 | -0.29187 | 0.000136 | 0.001943 |
| ENSRNOG00000009681 | *Flot2* | 0.733 | -0.44855 | 4.64E-12 | 2.48E-10 |
| ENSRNOG00000009713 | *Oxa1l* | 0.806 | -0.31066 | 5.03E-05 | 0.000824 |
| ENSRNOG00000009789 | *Topbp1* | 0.811 | -0.3029 | 0.00061 | 0.006928 |
| ENSRNOG00000009867 | *Tgfb3* | 1.367 | 0.450719 | 0 | 0 |
| ENSRNOG00000009919 | *Acod1* | 22.22 | 4.473807 | 9.23E-07 | 2.34E-05 |
| ENSRNOG00000009974 | *Coq3* | 0.68 | -0.55554 | 0.002144 | 0.018842 |
| ENSRNOG00000010017 | *Wee1* | 0.755 | -0.40637 | 1.77E-05 | 0.000327 |
| ENSRNOG00000010058 | *Spry2* | 1.29 | 0.367607 | 0.000135 | 0.001932 |
| ENSRNOG00000010071 | *RGD1560010* | 0.645 | -0.63284 | 2.65E-05 | 0.000469 |
| ENSRNOG00000010165 | *Tnfaip2* | 1.417 | 0.502848 | 1.02E-15 | 7.69E-14 |
| ENSRNOG00000010171 | *Elk1* | 0.818 | -0.28917 | 0.001903 | 0.017206 |
| ENSRNOG00000010183 | *Gask1b* | 1.212 | 0.277865 | 1.12E-19 | 1.16E-17 |
| ENSRNOG00000010274 | *Smc4* | 0.728 | -0.45812 | 2.42E-09 | 9.4E-08 |
| ENSRNOG00000010319 | *Lcp1* | 1.388 | 0.472694 | 8.47E-40 | 1.81E-37 |
| ENSRNOG00000010407 | *Pex5* | 0.756 | -0.40397 | 5.05E-05 | 0.000824 |
| ENSRNOG00000010421 | *Wdr91* | 0.721 | -0.47097 | 9.21E-06 | 0.000185 |
| ENSRNOG00000010447 | *Tmod2* | 4.666 | 2.222268 | 0.000966 | 0.009988 |
| ENSRNOG00000010468 | *Elovl6* | 0.596 | -0.74747 | 4.66E-05 | 0.000771 |
| ENSRNOG00000010558 | *Ppif* | 0.787 | -0.3459 | 0.004047 | 0.030794 |
| ENSRNOG00000010642 | *Lysmd2* | 1.228 | 0.296036 | 0.003055 | 0.024716 |
| ENSRNOG00000010646 | *Tmem229b* | 1.282 | 0.357882 | 0.000845 | 0.008987 |
| ENSRNOG00000010664 | *Wdr73* | 0.743 | -0.42785 | 8.21E-05 | 0.001262 |
| ENSRNOG00000010666 | *Ccn5* | 0.817 | -0.29196 | 2.81E-10 | 1.23E-08 |
| ENSRNOG00000010716 | *Atoh8* | 0.488 | -1.03512 | 1.01E-05 | 0.0002 |
| ENSRNOG00000010720 | *Mast4* | 0.673 | -0.57121 | 2.5E-10 | 1.11E-08 |
| ENSRNOG00000010721 | *Dlgap5* | 0.713 | -0.48704 | 7.64E-07 | 1.98E-05 |
| ENSRNOG00000010728 | *Stradb* | 0.786 | -0.34828 | 0.00144 | 0.013854 |
| ENSRNOG00000010760 | *E2f5* | 0.737 | -0.43998 | 0.001153 | 0.01156 |
| ENSRNOG00000010791 | *Ripor3* | 0.797 | -0.32786 | 0.001326 | 0.012992 |
| ENSRNOG00000010896 | *Tprn* | 0.766 | -0.38486 | 4.28E-05 | 0.000714 |
| ENSRNOG00000010910 | *Gmeb1* | 0.735 | -0.44337 | 0.003597 | 0.028108 |
| ENSRNOG00000010999 | *Cep295* | 0.826 | -0.27513 | 0.003549 | 0.02781 |
| ENSRNOG00000011000 | *Rims1* | 1.467 | 0.552772 | 0.000653 | 0.007302 |
| ENSRNOG00000011032 | *Lhfpl2* | 1.285 | 0.361302 | 0.001559 | 0.014682 |
| ENSRNOG00000011076 | *Ank2* | 0.732 | -0.44974 | 0.001026 | 0.010504 |
| ENSRNOG00000011078 | *Srm* | 0.514 | -0.95929 | 5.23E-05 | 0.000848 |
| ENSRNOG00000011096 | *Hmgb3* | 0.807 | -0.3095 | 0.000247 | 0.003234 |
| ENSRNOG00000011133 | *Trpc4* | 2.592 | 1.374271 | 0.002274 | 0.019718 |
| ENSRNOG00000011135 | *Gtf2b* | 0.802 | -0.31777 | 0.002067 | 0.018335 |
| ENSRNOG00000011227 | *Atp1b2* | 1.335 | 0.416365 | 3.9E-05 | 0.000658 |
| ENSRNOG00000011316 | *Fam167a* | 1.385 | 0.469514 | 5.95E-10 | 2.5E-08 |
| ENSRNOG00000011351 | *Mat1a* | 1.377 | 0.461874 | 5.1E-11 | 2.43E-09 |
| ENSRNOG00000011421 | *Smap2* | 1.202 | 0.265769 | 2.53E-06 | 5.82E-05 |
| ENSRNOG00000011459 | *Rhbdf2* | 1.33 | 0.411866 | 7.11E-05 | 0.001121 |
| ENSRNOG00000011498 | *Psip1* | 0.799 | -0.32398 | 0.000187 | 0.002549 |
| ENSRNOG00000011542 | *Apopt1* | 0.677 | -0.56349 | 0.000221 | 0.002939 |
| ENSRNOG00000011552 | *Mon1b* | 0.004 | -7.98882 | 6.48E-27 | 9.83E-25 |
| ENSRNOG00000011584 | *Gpatch1* | 0.806 | -0.311 | 0.002307 | 0.019954 |
| ENSRNOG00000011621 | *Hnrnpc* | 1.412 | 0.498133 | 3.95E-11 | 1.91E-09 |
| ENSRNOG00000011639 | *Mrpl47* | 0.728 | -0.45804 | 0.006829 | 0.046287 |
| ENSRNOG00000011671 | *LOC498933* | 1.388 | 0.472561 | 0.00225 | 0.019554 |
| ENSRNOG00000011692 | *Dkk1* | 15.999 | 3.999875 | 2.99E-87 | 2E-84 |
| ENSRNOG00000011774 | *Fblim1* | 1.255 | 0.327998 | 2.02E-07 | 5.75E-06 |
| ENSRNOG00000011775 | *Mfap3l* | 1.27 | 0.345227 | 0.000177 | 0.00245 |
| ENSRNOG00000011777 | *Spag5* | 0.64 | -0.64496 | 1.02E-10 | 4.72E-09 |
| ENSRNOG00000011800 | *F3* | 1.669 | 0.738696 | 2.38E-36 | 4.73E-34 |
| ENSRNOG00000011820 | *Acpp* | 1.528 | 0.61131 | 1.4E-05 | 0.000265 |
| ENSRNOG00000011946 | *Ptn* | 0.148 | -2.75501 | 0.002778 | 0.022943 |
| ENSRNOG00000011962 | *Gin1* | 0.586 | -0.77058 | 5.05E-05 | 0.000825 |
| ENSRNOG00000012014 | *Fam160b2* | 0.784 | -0.35036 | 0.000461 | 0.00553 |
| ENSRNOG00000012040 | *Slc25a48* | 19.554 | 4.289382 | 0.001271 | 0.012602 |
| ENSRNOG00000012067 | *Fam111a* | 0.703 | -0.50755 | 2.07E-07 | 5.85E-06 |
| ENSRNOG00000012086 | *AABR07026271.1* | 1.568 | 0.649256 | 0.000202 | 0.002713 |
| ENSRNOG00000012208 | *Itgb7* | 4.444 | 2.151879 | 0.006256 | 0.04328 |
| ENSRNOG00000012216 | *Tgfbi* | 0.746 | -0.4234 | 5.35E-24 | 6.98E-22 |
| ENSRNOG00000012287 | *Slc35e1* | 3.854 | 1.946409 | 3.43E-05 | 0.00059 |
| ENSRNOG00000012318 | *Aspm* | 0.683 | -0.55069 | 5.2E-13 | 3.08E-11 |
| ENSRNOG00000012356 | *Slc36a1* | 0.729 | -0.45588 | 1.13E-06 | 2.81E-05 |
| ENSRNOG00000012439 | *Bid* | 1.225 | 0.293054 | 0.00308 | 0.024894 |
| ENSRNOG00000012440 | *Msra* | 0.284 | -1.81391 | 0.000819 | 0.008775 |
| ENSRNOG00000012494 | *Kctd14* | 2.133 | 1.092985 | 0.002841 | 0.023335 |
| ENSRNOG00000012720 | *Irx4* | 3.111 | 1.637305 | 0.007124 | 0.047842 |
| ENSRNOG00000012721 | *Ednra* | 0.788 | -0.34317 | 1.89E-07 | 5.43E-06 |
| ENSRNOG00000012722 | *Ppdpf* | 0.83 | -0.26903 | 0.00218 | 0.019093 |
| ENSRNOG00000012729 | *Mfsd8* | 0.634 | -0.65724 | 0.000148 | 0.002095 |
| ENSRNOG00000012785 | *Armc10* | 0.826 | -0.27513 | 0.003549 | 0.02781 |
| ENSRNOG00000012835 | *Espl1* | 0.569 | -0.81355 | 1.53E-21 | 1.78E-19 |
| ENSRNOG00000012956 | *Tgm2* | 1.386 | 0.471083 | 2.27E-13 | 1.41E-11 |
| ENSRNOG00000012965 | *Themis2* | 0.474 | -1.07694 | 0.002457 | 0.020874 |
| ENSRNOG00000013057 | *Prc1* | 0.625 | -0.67804 | 6.21E-36 | 1.21E-33 |
| ENSRNOG00000013069 | *Sapcd2* | 0.556 | -0.84812 | 1.19E-11 | 6.1E-10 |
| ENSRNOG00000013102 | *Entpd2* | 1.405 | 0.4901 | 0.000601 | 0.006852 |
| ENSRNOG00000013112 | *Dusp13* | 60.013 | 5.907193 | 4.26E-09 | 1.62E-07 |
| ENSRNOG00000013167 | *Hmgb2* | 0.689 | -0.53814 | 5.49E-10 | 2.32E-08 |
| ENSRNOG00000013376 | *Mvd* | 0.808 | -0.30842 | 6.97E-16 | 5.34E-14 |
| ENSRNOG00000013397 | *Foxo1* | 1.46 | 0.546157 | 0.005436 | 0.03873 |
| ENSRNOG00000013399 | *Minar1* | 0.339 | -1.56237 | 0.005793 | 0.040785 |
| ENSRNOG00000013428 | *Atp6v0a4* | 0.796 | -0.32966 | 0.00148 | 0.014152 |
| ENSRNOG00000013445 | *Aaas* | 0.735 | -0.44391 | 1.74E-05 | 0.000321 |
| ENSRNOG00000013459 | *Ints9* | 0.747 | -0.42133 | 0.000732 | 0.008026 |
| ENSRNOG00000013515 | *Ptpru* | 2.085 | 1.060248 | 0.00103 | 0.010519 |
| ENSRNOG00000013598 | *Melk* | 0.56 | -0.83593 | 2.69E-06 | 6.13E-05 |
| ENSRNOG00000013653 | *Pdlim7* | 1.234 | 0.302827 | 2.85E-16 | 2.29E-14 |
| ENSRNOG00000013656 | *Lpar1* | 0.162 | -2.62948 | 0.005213 | 0.037621 |
| ENSRNOG00000013663 | *Tmem86a* | 0.612 | -0.70942 | 0.000936 | 0.009745 |
| ENSRNOG00000013727 | *Ndc80* | 0.678 | -0.56133 | 3.22E-05 | 0.00056 |
| ENSRNOG00000013887 | *Adra2b* | 1.479 | 0.564217 | 0.001148 | 0.011514 |
| ENSRNOG00000013940 | *RGD1565989* | 1.725 | 0.78683 | 7.35E-05 | 0.001145 |
| ENSRNOG00000013956 | *Rnf38* | 0.777 | -0.36447 | 4.94E-06 | 0.000105 |
| ENSRNOG00000013971 | *Psat1* | 0.802 | -0.31788 | 6.28E-06 | 0.000131 |
| ENSRNOG00000014019 | *Tbc1d7* | 0.809 | -0.30629 | 0.001814 | 0.016646 |
| ENSRNOG00000014021 | *Matn4* | 1.728 | 0.789308 | 0.006081 | 0.042264 |
| ENSRNOG00000014027 | *RGD1304728* | 0.713 | -0.48715 | 2.37E-07 | 6.62E-06 |
| ENSRNOG00000014061 | *Dusp5* | 1.261 | 0.334423 | 0.00403 | 0.030675 |
| ENSRNOG00000014072 | *Txndc17* | 0.816 | -0.29318 | 3.55E-07 | 9.57E-06 |
| ENSRNOG00000014080 | *Kif23* | 0.652 | -0.61711 | 8.11E-13 | 4.7E-11 |
| ENSRNOG00000014089 | *Map3k2* | 0.751 | -0.41365 | 0.000252 | 0.003289 |
| ENSRNOG00000014093 | *Tom1* | 0.77 | -0.37743 | 6.63E-10 | 2.75E-08 |
| ENSRNOG00000014096 | *AABR07031756.1* | 0.828 | -0.27221 | 1.52E-09 | 6.12E-08 |
| ENSRNOG00000014161 | *Rbm15b* | 1.349 | 0.431756 | 1.7E-10 | 7.63E-09 |
| ENSRNOG00000014297 | *Sdc4* | 1.2 | 0.2627 | 2.3E-12 | 1.27E-10 |
| ENSRNOG00000014331 | *Zfp810* | 4.249 | 2.087027 | 1.67E-06 | 4.02E-05 |
| ENSRNOG00000014343 | *Anln* | 0.725 | -0.46357 | 7.15E-19 | 7.02E-17 |
| ENSRNOG00000014357 | *Gja4* | 2.168 | 1.116255 | 5.76E-10 | 2.43E-08 |
| ENSRNOG00000014443 | *Pde5a* | 0.711 | -0.49198 | 0.000268 | 0.003464 |
| ENSRNOG00000014513 | *Rexo5* | 0.772 | -0.37391 | 0.000601 | 0.006848 |
| ENSRNOG00000014551 | *Ccnj* | 37.881 | 5.243409 | 3.37E-06 | 7.4E-05 |
| ENSRNOG00000014605 | *Lig4* | 0.698 | -0.51797 | 0.00193 | 0.017407 |
| ENSRNOG00000014613 | *Ddah1* | 1.212 | 0.27704 | 1.16E-07 | 3.5E-06 |
| ENSRNOG00000014648 | *Efnb2* | 1.267 | 0.340848 | 3.03E-07 | 8.27E-06 |
| ENSRNOG00000014650 | *Sema4g* | 1.792 | 0.841728 | 0.000122 | 0.001778 |
| ENSRNOG00000014684 | *Npr1* | 1.387 | 0.471815 | 0.005335 | 0.038157 |
| ENSRNOG00000014806 | *Pnkd* | 0.765 | -0.38566 | 2.59E-10 | 1.14E-08 |
| ENSRNOG00000014948 | *Osgin1* | 0.601 | -0.73565 | 0.004885 | 0.035718 |
| ENSRNOG00000014960 | *Bard1* | 0.656 | -0.60879 | 3.3E-05 | 0.00057 |
| ENSRNOG00000014984 | *Dmwd* | 0.738 | -0.43894 | 1.34E-05 | 0.000255 |
| ENSRNOG00000015036 | *Ccn2* | 1.291 | 0.369012 | 2.8E-99 | 2.37E-96 |
| ENSRNOG00000015055 | *Scg2* | 2.99 | 1.579972 | 0.000499 | 0.005861 |
| ENSRNOG00000015078 | *Ifitm3* | 1.768 | 0.821761 | 6.55E-15 | 4.56E-13 |
| ENSRNOG00000015117 | *Lrrc39* | 0.558 | -0.84219 | 0.006472 | 0.044337 |
| ENSRNOG00000015133 | *Kmt2a* | 0.825 | -0.27795 | 1.7E-09 | 6.77E-08 |
| ENSRNOG00000015143 | *Siah1* | 0.796 | -0.32895 | 0.00459 | 0.033978 |
| ENSRNOG00000015192 | *Mrps5* | 1.227 | 0.294811 | 0.001092 | 0.011055 |
| ENSRNOG00000015233 | *Etfa* | 0.828 | -0.27167 | 1.13E-06 | 2.8E-05 |
| ENSRNOG00000015237 | *Gle1* | 0.816 | -0.29328 | 0.001424 | 0.013745 |
| ENSRNOG00000015250 | *Mettl14* | 0.795 | -0.33104 | 0.004598 | 0.034011 |
| ENSRNOG00000015262 | *Slc34a1* | 3.111 | 1.637305 | 0.007124 | 0.047842 |
| ENSRNOG00000015275 | *Ska1* | 0.565 | -0.8236 | 1.97E-05 | 0.000357 |
| ENSRNOG00000015308 | *Pbk* | 0.461 | -1.11852 | 1.05E-06 | 2.63E-05 |
| ENSRNOG00000015310 | *Lrrc32* | 1.336 | 0.41786 | 4.17E-05 | 0.000697 |
| ENSRNOG00000015329 | *Kpna2* | 0.727 | -0.46025 | 1.44E-29 | 2.46E-27 |
| ENSRNOG00000015375 | *Lsm1* | 0.795 | -0.33051 | 0.003543 | 0.027794 |
| ENSRNOG00000015423 | *Ccna2* | 0.598 | -0.74263 | 1.94E-15 | 1.4E-13 |
| ENSRNOG00000015495 | *Slc25a37* | 1.683 | 0.750822 | 8.2E-09 | 3.01E-07 |
| ENSRNOG00000015496 | *Tpm4* | 1.295 | 0.372503 | 1.85E-68 | 7.93E-66 |
| ENSRNOG00000015529 | *Cdca3* | 0.426 | -1.23083 | 1.75E-40 | 3.79E-38 |
| ENSRNOG00000015588 | *Nol3* | 0.771 | -0.37572 | 1.83E-08 | 6.29E-07 |
| ENSRNOG00000015614 | *Ppp1r16b* | 4.74 | 2.244988 | 0.003746 | 0.029075 |
| ENSRNOG00000015733 | *Myl12b* | 0.809 | -0.30642 | 9.24E-05 | 0.001399 |
| ENSRNOG00000015794 | *Fam83d* | 0.572 | -0.80654 | 5.65E-11 | 2.66E-09 |
| ENSRNOG00000015810 | *Trip13* | 0.745 | -0.42561 | 0.000456 | 0.005477 |
| ENSRNOG00000015818 | *Tprkb* | 69.043 | 6.109422 | 3.15E-10 | 1.36E-08 |
| ENSRNOG00000015911 | *Lrp5* | 1.237 | 0.306962 | 4.53E-06 | 9.68E-05 |
| ENSRNOG00000015916 | *Ttc38* | 0.765 | -0.38563 | 0.000825 | 0.00883 |
| ENSRNOG00000015945 | *Cd3g* | 5.777 | 2.53039 | 0.005063 | 0.036767 |
| ENSRNOG00000015969 | *Rpf1* | 1.263 | 0.336718 | 0.002639 | 0.022018 |
| ENSRNOG00000016010 | *Mul1* | 0.785 | -0.34944 | 0.002738 | 0.022681 |
| ENSRNOG00000016021 | *Lims2* | 1.377 | 0.461588 | 9.23E-09 | 3.34E-07 |
| ENSRNOG00000016067 | *Ckap5* | 0.822 | -0.28358 | 4.44E-17 | 3.78E-15 |
| ENSRNOG00000016123 | *Rnf144b* | 1.66 | 0.730972 | 0.006263 | 0.043315 |
| ENSRNOG00000016156 | *Nptxr* | 0.802 | -0.31913 | 2.72E-48 | 7.33E-46 |
| ENSRNOG00000016196 | *Dlgap1* | 0.564 | -0.8254 | 0.00412 | 0.031263 |
| ENSRNOG00000016203 | *Efna2* | 2.058 | 1.041455 | 0.006012 | 0.041853 |
| ENSRNOG00000016299 | *Klf4* | 1.327 | 0.408654 | 0.005957 | 0.041607 |
| ENSRNOG00000016303 | *Zfp236* | 0.799 | -0.32456 | 0.002385 | 0.020417 |
| ENSRNOG00000016338 | *Fam92a* | 0.742 | -0.42992 | 0.00653 | 0.044624 |
| ENSRNOG00000016368 | *Ppp1r14c* | 0.827 | -0.27351 | 1.76E-06 | 4.21E-05 |
| ENSRNOG00000016377 | *Cep55* | 0.648 | -0.62501 | 1.28E-08 | 4.51E-07 |
| ENSRNOG00000016390 | *Eef1e1* | 1.281 | 0.357688 | 0.000264 | 0.003426 |
| ENSRNOG00000016399 | *RGD1359127* | 0.823 | -0.28063 | 0.000653 | 0.0073 |
| ENSRNOG00000016454 | *Nasp* | 0.735 | -0.44416 | 3.62E-06 | 7.89E-05 |
| ENSRNOG00000016488 | *Pltp* | 1.776 | 0.828832 | 0.00023 | 0.003038 |
| ENSRNOG00000016538 | *Itga8* | 1.231 | 0.299933 | 5.93E-06 | 0.000124 |
| ENSRNOG00000016565 | *Zfp524* | 0.72 | -0.47463 | 0.002005 | 0.017899 |
| ENSRNOG00000016689 | *Fanci* | 0.77 | -0.37674 | 0.000107 | 0.001593 |
| ENSRNOG00000016748 | *Poll* | 0.821 | -0.28414 | 0.004413 | 0.032994 |
| ENSRNOG00000016751 | *Slc25a28* | 0.829 | -0.27083 | 0.001696 | 0.015744 |
| ENSRNOG00000016756 | *Ptgir* | 1.892 | 0.919588 | 0.000667 | 0.007426 |
| ENSRNOG00000016780 | *RGD1310951* | 0.621 | -0.68711 | 0.004991 | 0.03635 |
| ENSRNOG00000016810 | *Stmn1* | 0.732 | -0.44954 | 2.73E-11 | 1.34E-09 |
| ENSRNOG00000016827 | *Slc38a3* | 1.449 | 0.534753 | 0.000837 | 0.008925 |
| ENSRNOG00000016866 | *Fhl2* | 1.213 | 0.278798 | 7.26E-14 | 4.68E-12 |
| ENSRNOG00000016879 | *Ldlrad4* | 1.409 | 0.494375 | 1.1E-05 | 0.000214 |
| ENSRNOG00000016883 | *Entpd7* | 1.264 | 0.338343 | 0.005934 | 0.04148 |
| ENSRNOG00000016889 | *Fancc* | 0.622 | -0.68462 | 0.006003 | 0.041806 |
| ENSRNOG00000016945 | *Pla2g2a* | 15.999 | 3.999875 | 0.004265 | 0.032132 |
| ENSRNOG00000017047 | *Inip* | 0.79 | -0.33933 | 0.006832 | 0.04629 |
| ENSRNOG00000017075 | *Slc35e2b* | 0.656 | -0.60763 | 0.00029 | 0.003718 |
| ENSRNOG00000017137 | *Eef1akmt2* | 0.653 | -0.61435 | 1.16E-12 | 6.61E-11 |
| ENSRNOG00000017212 | *Spsb1* | 1.211 | 0.276047 | 3.81E-08 | 1.25E-06 |
| ENSRNOG00000017291 | *Sord* | 0.814 | -0.29746 | 2.17E-08 | 7.31E-07 |
| ENSRNOG00000017307 | *Prss23* | 1.277 | 0.3524 | 5.03E-67 | 2E-64 |
| ENSRNOG00000017321 | *Trub1* | 1.298 | 0.376585 | 0.006737 | 0.045756 |
| ENSRNOG00000017369 | *Mustn1* | 0.556 | -0.84592 | 2.39E-05 | 0.000429 |
| ENSRNOG00000017512 | *Aldh3b1* | 0.701 | -0.51325 | 0.004707 | 0.034644 |
| ENSRNOG00000017581 | *RGD1306227* | 0.516 | -0.95592 | 0.003807 | 0.029343 |
| ENSRNOG00000017628 | *Tagln* | 1.336 | 0.418279 | 5.9E-166 | 1.1E-162 |
| ENSRNOG00000017647 | *Zc3h8* | 0.712 | -0.48917 | 0.000273 | 0.003516 |
| ENSRNOG00000017850 | *Dctpp1* | 0.477 | -1.06748 | 1.25E-05 | 0.00024 |
| ENSRNOG00000017912 | *Atp2a3* | 1.363 | 0.447278 | 0.000739 | 0.008086 |
| ENSRNOG00000017918 | *Iglon5* | 1.491 | 0.576376 | 7.27E-05 | 0.001138 |
| ENSRNOG00000017960 | *Deaf1* | 0.8 | -0.32233 | 7.57E-08 | 2.35E-06 |
| ENSRNOG00000018110 | *Svil* | 0.811 | -0.3025 | 0.003769 | 0.029165 |
| ENSRNOG00000018111 | *Slc12a5* | 1.62 | 0.696199 | 0.000101 | 0.001511 |
| ENSRNOG00000018230 | *Gtf2h2* | 0.691 | -0.53262 | 0.000743 | 0.008114 |
| ENSRNOG00000018232 | *Srf* | 1.237 | 0.306449 | 3.26E-10 | 1.41E-08 |
| ENSRNOG00000018262 | *Ampd3* | 2.296 | 1.199184 | 2.73E-13 | 1.66E-11 |
| ENSRNOG00000018294 | *Hspa5* | 1.389 | 0.47402 | 5.5E-169 | 1.1E-165 |
| ENSRNOG00000018297 | *Ocln* | 1.845 | 0.88339 | 0.000153 | 0.002152 |
| ENSRNOG00000018371 | *Tubb6* | 1.217 | 0.283296 | 9.04E-18 | 8.08E-16 |
| ENSRNOG00000018384 | *Adam12* | 1.588 | 0.667526 | 8.92E-44 | 2.22E-41 |
| ENSRNOG00000018413 | *Per3* | 0.676 | -0.56491 | 6.07E-11 | 2.85E-09 |
| ENSRNOG00000018445 | *Agt* | 0.205 | -2.28553 | 2.1E-08 | 7.08E-07 |
| ENSRNOG00000018461 | *Pdgfrb* | 1.21 | 0.275357 | 7.75E-73 | 3.59E-70 |
| ENSRNOG00000018483 | *Smad1* | 1.211 | 0.276393 | 0.000481 | 0.005696 |
| ENSRNOG00000018531 | *Mrps11* | 1.207 | 0.272004 | 0.003535 | 0.027742 |
| ENSRNOG00000018533 | *Iffo1* | 0.81 | -0.30381 | 0.000608 | 0.006907 |
| ENSRNOG00000018598 | *Ankrd1* | 1.382 | 0.46648 | 7.5E-134 | 8.9E-131 |
| ENSRNOG00000018659 | *Csf1* | 1.331 | 0.41247 | 6.32E-19 | 6.28E-17 |
| ENSRNOG00000018694 | *Lipg* | 1.932 | 0.950245 | 0.006637 | 0.045259 |
| ENSRNOG00000018842 | *Pou4f3* | 8.888 | 3.151879 | 0.005968 | 0.041653 |
| ENSRNOG00000018886 | *Prxl2c* | 1.337 | 0.419017 | 2E-05 | 0.000363 |
| ENSRNOG00000018906 | *Ghdc* | 0.743 | -0.42785 | 0.000873 | 0.009236 |
| ENSRNOG00000018929 | *Kif20b* | 0.627 | -0.67273 | 9.38E-15 | 6.42E-13 |
| ENSRNOG00000018939 | *Rexo2* | 1.235 | 0.304233 | 3.86E-08 | 1.26E-06 |
| ENSRNOG00000018944 | *Pank1* | 1.312 | 0.392079 | 4.58E-18 | 4.16E-16 |
| ENSRNOG00000018962 | *Ctf1* | 0.776 | -0.36567 | 4.92E-06 | 0.000105 |
| ENSRNOG00000018971 | *Mob3a* | 1.272 | 0.346849 | 0.000106 | 0.001577 |
| ENSRNOG00000019048 | *Sod2* | 1.236 | 0.305881 | 1.02E-07 | 3.11E-06 |
| ENSRNOG00000019079 | *Polr1c* | 0.829 | -0.2698 | 0.004413 | 0.033017 |
| ENSRNOG00000019098 | *Car5a* | 0.056 | -4.14825 | 0.004144 | 0.031387 |
| ENSRNOG00000019100 | *Kif2c* | 0.506 | -0.98307 | 2.45E-16 | 2E-14 |
| ENSRNOG00000019103 | *Cln3* | 0.458 | -1.12698 | 0.007133 | 0.047861 |
| ENSRNOG00000019127 | *Zfp606* | 0.633 | -0.66038 | 0.001228 | 0.012227 |
| ENSRNOG00000019150 | *Polb* | 0.688 | -0.53877 | 0.000144 | 0.002046 |
| ENSRNOG00000019206 | *Nupr1* | 0.773 | -0.37158 | 2.1E-55 | 6.6E-53 |
| ENSRNOG00000019229 | *Fbl* | 1.217 | 0.28324 | 0.00014 | 0.001997 |
| ENSRNOG00000019249 | *Ap3b2* | 0.714 | -0.48608 | 0.000422 | 0.005121 |
| ENSRNOG00000019330 | *Procr* | 1.866 | 0.900114 | 4.79E-58 | 1.54E-55 |
| ENSRNOG00000019351 | *Chid1* | 2.622 | 1.390766 | 3.29E-09 | 1.26E-07 |
| ENSRNOG00000019383 | *Tef* | 0.758 | -0.39977 | 2.68E-11 | 1.32E-09 |
| ENSRNOG00000019414 | *Tmem79* | 1.436 | 0.522385 | 0.005435 | 0.038744 |
| ENSRNOG00000019438 | *Rnf31* | 0.794 | -0.33319 | 0.001586 | 0.014893 |
| ENSRNOG00000019656 | *Btd* | 0.776 | -0.36621 | 1.17E-11 | 6.01E-10 |
| ENSRNOG00000019723 | *Rbm12* | 1.204 | 0.268351 | 0.001046 | 0.010667 |
| ENSRNOG00000019735 | *Dph2* | 0.532 | -0.91041 | 2.85E-06 | 6.47E-05 |
| ENSRNOG00000019772 | *Dnpep* | 1.208 | 0.272246 | 1.15E-16 | 9.59E-15 |
| ENSRNOG00000019802 | *Zfp428* | 0.785 | -0.34932 | 0.001439 | 0.013852 |
| ENSRNOG00000019810 | *Des* | 1.206 | 0.270326 | 1.99E-08 | 6.77E-07 |
| ENSRNOG00000019851 | *Cox6a2* | 0.513 | -0.96224 | 1.91E-09 | 7.53E-08 |
| ENSRNOG00000019914 | *Fam57b* | 0.794 | -0.33364 | 4.79E-05 | 0.000789 |
| ENSRNOG00000019918 | *Coasy* | 0.802 | -0.3182 | 1.92E-05 | 0.00035 |
| ENSRNOG00000019965 | *Tgfb1i1* | 1.215 | 0.281173 | 1.14E-15 | 8.56E-14 |
| ENSRNOG00000019982 | *Ethe1* | 1.213 | 0.278177 | 0.004239 | 0.032018 |
| ENSRNOG00000019996 | *Slc16a1* | 0.825 | -0.27778 | 0.000128 | 0.00185 |
| ENSRNOG00000020097 | *Inha* | 0.695 | -0.52528 | 0.00012 | 0.00176 |
| ENSRNOG00000020113 | *Cnnm2* | 1.294 | 0.371591 | 2.77E-09 | 1.07E-07 |
| ENSRNOG00000020133 | *LOC108348044* | 0.478 | -1.06592 | 1.59E-06 | 3.87E-05 |
| ENSRNOG00000020244 | *Perm1* | 0.205 | -2.28553 | 0.005082 | 0.036819 |
| ENSRNOG00000020250 | *Pcgf6* | 0.772 | -0.37402 | 0.000384 | 0.004706 |
| ENSRNOG00000020263 | *Atp1a3* | 3.456 | 1.789308 | 0.000217 | 0.002898 |
| ENSRNOG00000020269 | *Sugp2* | 0.822 | -0.28343 | 0.001955 | 0.017575 |
| ENSRNOG00000020279 | *Syt11* | 0.807 | -0.30916 | 7.28E-05 | 0.001138 |
| ENSRNOG00000020281 | *Kif22* | 0.646 | -0.63138 | 1.22E-18 | 1.17E-16 |
| ENSRNOG00000020295 | *Plekhn1* | 0.503 | -0.99108 | 0.00214 | 0.018822 |
| ENSRNOG00000020372 | *Hdac4* | 0.828 | -0.27286 | 0.0003 | 0.003805 |
| ENSRNOG00000020389 | *Capn12* | 6.648 | 2.733027 | 0.001626 | 0.015193 |
| ENSRNOG00000020410 | *Th* | 1.646 | 0.718919 | 0.002501 | 0.02115 |
| ENSRNOG00000020433 | *Actn4* | 1.218 | 0.284208 | 3.1E-134 | 3.9E-131 |
| ENSRNOG00000020441 | *Wnk4* | 1.235 | 0.304779 | 1.58E-08 | 5.45E-07 |
| ENSRNOG00000020525 | *Col5a3* | 0.796 | -0.32894 | 3.99E-06 | 8.62E-05 |
| ENSRNOG00000020554 | *Slc50a1* | 0.774 | -0.36978 | 0.000646 | 0.007256 |
| ENSRNOG00000020607 | *Bckdha* | 0.794 | -0.33355 | 0.000356 | 0.00441 |
| ENSRNOG00000020650 | *Slc17a7* | 1.43 | 0.515842 | 0.000814 | 0.008731 |
| ENSRNOG00000020679 | *Icam1* | 1.391 | 0.476051 | 4.18E-38 | 8.61E-36 |
| ENSRNOG00000020694 | *Icam5* | 0.824 | -0.27857 | 0.000832 | 0.00888 |
| ENSRNOG00000020696 | *Pmvk* | 0.817 | -0.29127 | 3.28E-05 | 0.000568 |
| ENSRNOG00000020719 | *Hrc* | 0.458 | -1.12698 | 0.007133 | 0.047861 |
| ENSRNOG00000020721 | *Nme6* | 0.697 | -0.52152 | 0.00106 | 0.01078 |
| ENSRNOG00000020737 | *Cdc25a* | 0.805 | -0.31294 | 0.001384 | 0.013443 |
| ENSRNOG00000020751 | *Rdm1* | 0.689 | -0.53691 | 0.000196 | 0.002654 |
| ENSRNOG00000020762 | *Zfp260* | 1.428 | 0.513716 | 2.04E-10 | 9.06E-09 |
| ENSRNOG00000020769 | *Crebrf* | 0.749 | -0.4176 | 1.64E-05 | 0.000306 |
| ENSRNOG00000020774 | *LOC103690163* | 0.727 | -0.45958 | 0.000204 | 0.002735 |
| ENSRNOG00000020781 | *Tbcb* | 2.691 | 1.428325 | 1.26E-06 | 3.11E-05 |
| ENSRNOG00000020783 | *Ntf4* | 1.553 | 0.634934 | 0.000838 | 0.008926 |
| ENSRNOG00000020792 | *Etv4* | 1.263 | 0.336759 | 1.59E-06 | 3.87E-05 |
| ENSRNOG00000020811 | *Il6r* | 0.748 | -0.41836 | 0.002637 | 0.022006 |
| ENSRNOG00000020847 | *Elp6* | 0.724 | -0.46667 | 0.001079 | 0.01094 |
| ENSRNOG00000020848 | *Coq8b* | 0.775 | -0.36863 | 7.05E-06 | 0.000145 |
| ENSRNOG00000020884 | *Cd6* | 19.554 | 4.289382 | 0.001271 | 0.012602 |
| ENSRNOG00000020918 | *Ccnd1* | 1.337 | 0.419089 | 3.28E-51 | 9.61E-49 |
| ENSRNOG00000020952 | *Cgn* | 1.822 | 0.865226 | 3.33E-07 | 9.06E-06 |
| ENSRNOG00000020995 | *Fut1* | 0.395 | -1.33997 | 0.00529 | 0.037967 |
| ENSRNOG00000020996 | *Vps51* | 0.78 | -0.35927 | 1.8E-07 | 5.23E-06 |
| ENSRNOG00000021061 | *Map4k2* | 0.679 | -0.55932 | 0.001807 | 0.016591 |
| ENSRNOG00000021063 | *Grin2d* | 0.733 | -0.44739 | 0.001787 | 0.016421 |
| ENSRNOG00000021153 | *Fkbp2* | 1.205 | 0.269491 | 0.003362 | 0.026639 |
| ENSRNOG00000021174 | *Macrod1* | 2.127 | 1.088685 | 0.00044 | 0.005311 |
| ENSRNOG00000021243 | *Siglec1* | 0.54 | -0.88828 | 0.006001 | 0.041832 |
| ENSRNOG00000021248 | *Cdc25b* | 0.704 | -0.5067 | 1.2E-08 | 4.22E-07 |
| ENSRNOG00000021298 | *Dstyk* | 0.825 | -0.27675 | 1.54E-05 | 0.000288 |
| ENSRNOG00000021365 | *Ybey* | 0.593 | -0.75313 | 0.00272 | 0.022544 |
| ENSRNOG00000021380 | *Fads6* | 0.444 | -1.17005 | 0.002927 | 0.023895 |
| ENSRNOG00000021402 | *Ankrd53* | 1.83 | 0.871771 | 0.002817 | 0.023236 |
| ENSRNOG00000021412 | *Slfn13* | 0.793 | -0.33543 | 8.38E-13 | 4.84E-11 |
| ENSRNOG00000021440 | *Pptc7* | 0.823 | -0.28041 | 1.25E-06 | 3.09E-05 |
| ENSRNOG00000021528 | *Tusc2* | 0.819 | -0.28736 | 0.001551 | 0.014634 |
| ENSRNOG00000021536 | *Plxdc1* | 1.4 | 0.48496 | 0.002036 | 0.018146 |
| ENSRNOG00000021548 | *Rassf1* | 0.69 | -0.53467 | 7.69E-07 | 1.99E-05 |
| ENSRNOG00000021713 | *Kif18b* | 0.693 | -0.5285 | 2.36E-07 | 6.61E-06 |
| ENSRNOG00000021847 | *Ska3* | 0.642 | -0.64027 | 0.000374 | 0.004607 |
| ENSRNOG00000022082 | *Dusp11* | 2.603 | 1.380449 | 6.33E-08 | 2E-06 |
| ENSRNOG00000022325 | *Smc2* | 0.77 | -0.37632 | 1.12E-05 | 0.000217 |
| ENSRNOG00000022393 | *Faap24* | 0.65 | -0.62126 | 0.000193 | 0.002612 |
| ENSRNOG00000022499 | *Sgo1* | 0.527 | -0.92494 | 4.89E-09 | 1.85E-07 |
| ENSRNOG00000022597 | *Cenpj* | 0.631 | -0.66377 | 1.18E-07 | 3.53E-06 |
| ENSRNOG00000022657 | *Tmem97* | 0.815 | -0.29516 | 0.000392 | 0.004795 |
| ENSRNOG00000022911 | *Hjurp* | 0.542 | -0.88329 | 7.19E-14 | 4.66E-12 |
| ENSRNOG00000022929 | *Mtmr12* | 0.829 | -0.27069 | 0.000854 | 0.009068 |
| ENSRNOG00000023023 | *Trpt1* | 1.807 | 0.853977 | 0.003419 | 0.027025 |
| ENSRNOG00000023093 | *Mis18bp1* | 0.522 | -0.9376 | 1.47E-06 | 3.59E-05 |
| ENSRNOG00000023202 | *Usp15* | 0.818 | -0.28921 | 3.63E-05 | 0.000618 |
| ENSRNOG00000023346 | *Cplane2* | 0.501 | -0.99787 | 0.000352 | 0.004371 |
| ENSRNOG00000023352 | *Fam78a* | 1.25 | 0.322231 | 0.002866 | 0.023512 |
| ENSRNOG00000023403 | *Gtpbp3* | 0.671 | -0.57489 | 0.000312 | 0.003943 |
| ENSRNOG00000023465 | *Depp1* | 1.501 | 0.586327 | 2.05E-07 | 5.8E-06 |
| ENSRNOG00000023579 | *Tet2* | 0.796 | -0.32989 | 0.001436 | 0.013846 |
| ENSRNOG00000023587 | *Dhtkd1* | 1.237 | 0.307025 | 0.007079 | 0.047614 |
| ENSRNOG00000023593 | *Upf2* | 0.808 | -0.30755 | 0.00571 | 0.040255 |
| ENSRNOG00000023764 | *Kctd2* | 0.789 | -0.34149 | 0.002122 | 0.018713 |
| ENSRNOG00000023920 | *Zfp791* | 26.54 | 4.730093 | 0.000125 | 0.001817 |
| ENSRNOG00000023946 | *Dmac1* | 0.662 | -0.59455 | 0.006206 | 0.042969 |
| ENSRNOG00000024008 | *Cdc25c* | 0.793 | -0.33385 | 0.001862 | 0.01695 |
| ENSRNOG00000024239 | *Fam89b* | 0.795 | -0.33009 | 1.85E-11 | 9.18E-10 |
| ENSRNOG00000024365 | *Ect2* | 0.574 | -0.80014 | 3.08E-12 | 1.66E-10 |
| ENSRNOG00000024428 | *Kif20a* | 0.61 | -0.71219 | 4.02E-34 | 7.47E-32 |
| ENSRNOG00000024452 | *Carmil2* | 0.611 | -0.71093 | 7.76E-05 | 0.001206 |
| ENSRNOG00000024595 | *Cers6* | 1.282 | 0.358944 | 0.004291 | 0.032251 |
| ENSRNOG00000024650 | *Ckap2* | 0.587 | -0.76906 | 1.26E-18 | 1.21E-16 |
| ENSRNOG00000024730 | *Ppm1e* | 0.829 | -0.27128 | 0.003905 | 0.029931 |
| ENSRNOG00000025079 | *Fam126b* | 0.786 | -0.34759 | 0.005249 | 0.037784 |
| ENSRNOG00000025100 | *Ikbke* | 0.829 | -0.2714 | 0.000383 | 0.004699 |
| ENSRNOG00000025220 | *Slc6a16* | 5.555 | 2.473807 | 0.000124 | 0.001808 |
| ENSRNOG00000025269 | *Slc25a44* | 0.725 | -0.463 | 9.91E-06 | 0.000196 |
| ENSRNOG00000025302 | *Cdca2* | 0.53 | -0.91629 | 3.02E-12 | 1.64E-10 |
| ENSRNOG00000025742 | *Lmnb2* | 0.827 | -0.27452 | 0.000304 | 0.003847 |
| ENSRNOG00000026672 | *MGC94199* | 2.025 | 1.01784 | 3.26E-06 | 7.23E-05 |
| ENSRNOG00000026691 | *Coq4* | 0.513 | -0.96261 | 9.86E-09 | 3.55E-07 |
| ENSRNOG00000026907 | *Zbtb37* | 0.418 | -1.25751 | 0.00289 | 0.02366 |
| ENSRNOG00000026951 | *Susd5* | 1.247 | 0.318434 | 1.54E-13 | 9.6E-12 |
| ENSRNOG00000026981 | *Zfp282* | 0.794 | -0.33319 | 6.15E-05 | 0.000983 |
| ENSRNOG00000027012 | *Usp54* | 0.811 | -0.3021 | 0.007307 | 0.048714 |
| ENSRNOG00000027030 | *Adm* | 1.212 | 0.276893 | 0.00052 | 0.006062 |
| ENSRNOG00000027035 | *Sgo2* | 0.704 | -0.5061 | 0.000221 | 0.002941 |
| ENSRNOG00000027089 | *Ell2* | 0.804 | -0.31535 | 0.005835 | 0.041033 |
| ENSRNOG00000027220 | *Pcdhgb8* | 1.201 | 0.264258 | 0.004965 | 0.03621 |
| ENSRNOG00000027259 | *AABR07029661.1* | 0.726 | -0.46129 | 0.00091 | 0.009538 |
| ENSRNOG00000027263 | *Tmem267* | 1.757 | 0.81352 | 0.000636 | 0.007151 |
| ENSRNOG00000027430 | *Ikzf2* | 0.593 | -0.75501 | 0.002595 | 0.021778 |
| ENSRNOG00000027436 | *Zfp324* | 0.653 | -0.61428 | 0.001277 | 0.012609 |
| ENSRNOG00000027451 | *Mettl25* | 0.626 | -0.67559 | 0.003642 | 0.02841 |
| ENSRNOG00000027456 | *Cdc42bpg* | 1.294 | 0.371324 | 0.00275 | 0.022758 |
| ENSRNOG00000027542 | *LOC100302465* | 2.133 | 1.092985 | 0.002841 | 0.023335 |
| ENSRNOG00000027593 | *Ndufv3* | 2.093 | 1.065819 | 0.003323 | 0.026449 |
| ENSRNOG00000027646 | *Pop4* | 0.793 | -0.33461 | 0.000865 | 0.009173 |
| ENSRNOG00000027736 | *Cnn1* | 1.366 | 0.449443 | 3.67E-22 | 4.41E-20 |
| ENSRNOG00000027797 | *Nit2* | 0.82 | -0.28704 | 0.00591 | 0.041381 |
| ENSRNOG00000027839 | *Ptk2b* | 1.376 | 0.460717 | 7.18E-05 | 0.001128 |
| ENSRNOG00000027894 | *Iqgap3* | 0.69 | -0.53492 | 3.15E-06 | 7.03E-05 |
| ENSRNOG00000028063 | *Tmem38b* | 1.457 | 0.543069 | 0.00087 | 0.009211 |
| ENSRNOG00000028072 | *Chit1* | 1.452 | 0.538488 | 0.000925 | 0.00965 |
| ENSRNOG00000028129 | *Fktn* | 0.804 | -0.31539 | 5.16E-05 | 0.000839 |
| ENSRNOG00000028206 | *Pheta1* | 0.768 | -0.38133 | 0.000809 | 0.0087 |
| ENSRNOG00000028404 | *Ppp1r1b* | 1.302 | 0.380871 | 0.001354 | 0.013216 |
| ENSRNOG00000028415 | *Cdc20* | 0.619 | -0.6915 | 2.46E-12 | 1.34E-10 |
| ENSRNOG00000028556 | *Usp30* | 0.78 | -0.3583 | 7.95E-07 | 2.05E-05 |
| ENSRNOG00000028585 | *Tceal8* | 1.238 | 0.308344 | 1.77E-08 | 6.09E-07 |
| ENSRNOG00000028659 | *Szt2* | 0.69 | -0.5355 | 7.23E-08 | 2.25E-06 |
| ENSRNOG00000028668 | *Slc28a2* | 1.508 | 0.592745 | 3.6E-05 | 0.000614 |
| ENSRNOG00000028717 | *Ndufb7* | 1.272 | 0.346741 | 2.21E-05 | 0.000398 |
| ENSRNOG00000028781 | *Abcc6* | 0.787 | -0.34533 | 0.000357 | 0.004414 |
| ENSRNOG00000028856 | *Pknox2* | 8.888 | 3.151879 | 0.000101 | 0.001511 |
| ENSRNOG00000029055 | *Ttk* | 0.508 | -0.97849 | 3.16E-14 | 2.1E-12 |
| ENSRNOG00000029401 | *Actg2* | 1.4 | 0.485753 | 4.08E-80 | 2.35E-77 |
| ENSRNOG00000029571 | *Coq10a* | 0.772 | -0.37395 | 0.000229 | 0.003026 |
| ENSRNOG00000029651 | *Rdh16* | 1.466 | 0.552043 | 0.000901 | 0.009478 |
| ENSRNOG00000029773 | *Atm* | 0.809 | -0.3064 | 1.39E-05 | 0.000263 |
| ENSRNOG00000029830 | *Adm2* | 3.555 | 1.82995 | 0.004529 | 0.033693 |
| ENSRNOG00000029911 | *Cilp* | 0.663 | -0.59355 | 0.003866 | 0.029689 |
| ENSRNOG00000029939 | *Gypc* | 1.255 | 0.327329 | 9.39E-05 | 0.001417 |
| ENSRNOG00000030034 | *Sox11* | 0.816 | -0.29356 | 0.000651 | 0.007287 |
| ENSRNOG00000030334 | *Adck5* | 0.659 | -0.60094 | 1.92E-05 | 0.00035 |
| ENSRNOG00000030537 | *Slc26a11* | 0.796 | -0.32996 | 0.005967 | 0.041664 |
| ENSRNOG00000030750 | *Zfp2* | 0.311 | -1.68462 | 0.004474 | 0.033328 |
| ENSRNOG00000030932 | *Zfp14* | 4.096 | 2.034042 | 4.03E-11 | 1.95E-09 |
| ENSRNOG00000031031 | *Zfp292* | 0.762 | -0.39294 | 4.67E-05 | 0.000771 |
| ENSRNOG00000031093 | *Mov10l1* | 10.666 | 3.414913 | 0.001821 | 0.016687 |
| ENSRNOG00000031127 | *Snrpe* | 22.629 | 4.500111 | 0.000454 | 0.005465 |
| ENSRNOG00000031171 | *Wdr46* | 1.207 | 0.271855 | 0.004029 | 0.030696 |
| ENSRNOG00000031266 | *Siae* | 0.806 | -0.31114 | 0.001062 | 0.010799 |
| ENSRNOG00000031431 | *Cdca8* | 0.617 | -0.69645 | 5.4E-09 | 2.03E-07 |
| ENSRNOG00000031495 | *Tmem170b* | 0.662 | -0.59546 | 2.49E-05 | 0.000445 |
| ENSRNOG00000031706 | *RGD1563601* | 0.079 | -3.65904 | 0.00096 | 0.009937 |
| ENSRNOG00000031934 | *Enah* | 1.2 | 0.262881 | 1.79E-25 | 2.47E-23 |
| ENSRNOG00000032048 | *Zfp462* | 1.286 | 0.362558 | 0.000505 | 0.00591 |
| ENSRNOG00000032178 | *Cenpa* | 0.512 | -0.96473 | 2.88E-14 | 1.92E-12 |
| ENSRNOG00000032258 | *Swt1* | 0.79 | -0.33997 | 0.004052 | 0.030799 |
| ENSRNOG00000032303 | *LOC108349682* | 0.552 | -0.85729 | 6.55E-05 | 0.001045 |
| ENSRNOG00000032394 | *Tymp* | 0.791 | -0.3382 | 0.000277 | 0.003568 |
| ENSRNOG00000032446 | *Recql4* | 0.71 | -0.49397 | 0.002494 | 0.021105 |
| ENSRNOG00000032487 | *Sts* | 0.759 | -0.39871 | 0.00011 | 0.001635 |
| ENSRNOG00000032778 | *Bub1* | 0.688 | -0.5396 | 2.1E-12 | 1.17E-10 |
| ENSRNOG00000032835 | *LOC103692785* | 0.024 | -5.3836 | 0.004495 | 0.033454 |
| ENSRNOG00000032871 | *Mlc1* | 4.74 | 2.244988 | 0.003746 | 0.029075 |
| ENSRNOG00000032885 | *Cycs* | 1.691 | 0.757502 | 0.004652 | 0.03435 |
| ENSRNOG00000032929 | *Incenp* | 0.747 | -0.4199 | 2.73E-13 | 1.66E-11 |
| ENSRNOG00000033335 | *Cenpi* | 0.674 | -0.56831 | 8.53E-05 | 0.001311 |
| ENSRNOG00000033570 | *Arhgap8* | 1.369 | 0.453183 | 0.005818 | 0.040944 |
| ENSRNOG00000033658 | *Kntc1* | 0.814 | -0.29637 | 0.001454 | 0.013948 |
| ENSRNOG00000033772 | *Serpinb9* | 1.329 | 0.410517 | 1.22E-26 | 1.79E-24 |
| ENSRNOG00000034130 | *LOC108349189* | 2.337 | 1.2244 | 3.01E-08 | 9.96E-07 |
| ENSRNOG00000034200 | *Atp8a1* | 0.417 | -1.26316 | 0.003727 | 0.028951 |
| ENSRNOG00000034254 | *Actb* | 1.247 | 0.318536 | 0 | 0 |
| ENSRNOG00000035603 | *Mir143* | 1.331 | 0.412991 | 8.13E-10 | 3.34E-08 |
| ENSRNOG00000035631 | *Mir221* | 1.889 | 0.917413 | 0.002114 | 0.01865 |
| ENSRNOG00000036601 | *Exoc6* | 0.827 | -0.27452 | 0.002038 | 0.018134 |
| ENSRNOG00000036667 | *Hexd* | 0.798 | -0.32599 | 0.000973 | 0.010038 |
| ENSRNOG00000036942 | *Tnfrsf9* | 2.933 | 1.552416 | 0.001171 | 0.011712 |
| ENSRNOG00000037113 | *Slfn2* | 0.803 | -0.31654 | 2.9E-16 | 2.31E-14 |
| ENSRNOG00000037134 | *Shc4* | 1.393 | 0.478392 | 8.04E-06 | 0.000164 |
| ENSRNOG00000037198 | *Usp18* | 1.371 | 0.454739 | 0.004439 | 0.03311 |
| ENSRNOG00000037211 | *Kif14* | 0.519 | -0.94672 | 1.59E-09 | 6.38E-08 |
| ENSRNOG00000037509 | *Chek2* | 0.718 | -0.47868 | 0.000255 | 0.003315 |
| ENSRNOG00000037541 | *Sdhaf1* | 0.327 | -1.61212 | 3.3E-05 | 0.00057 |
| ENSRNOG00000037604 | *Ascc3* | 0.819 | -0.28834 | 0.002662 | 0.022115 |
| ENSRNOG00000037688 | *Ak9* | 2.173 | 1.119457 | 0.003577 | 0.027994 |
| ENSRNOG00000037931 | *Plaur* | 1.2 | 0.263516 | 8.7E-05 | 0.001334 |
| ENSRNOG00000037984 | *Sccpdh* | 0.001 | -9.80155 | 8.94E-73 | 4.04E-70 |
| ENSRNOG00000038035 | *Kif4a* | 0.529 | -0.91994 | 2.41E-18 | 2.25E-16 |
| ENSRNOG00000038165 | *Pjvk* | 4.222 | 2.077878 | 0.002616 | 0.021898 |
| ENSRNOG00000039183 | *Cip2a* | 0.528 | -0.92212 | 7.04E-06 | 0.000145 |
| ENSRNOG00000039278 | *Mcart1* | 0.665 | -0.58758 | 2.07E-06 | 4.89E-05 |
| ENSRNOG00000042286 | *Nsl1* | 0.562 | -0.83218 | 1.26E-05 | 0.000241 |
| ENSRNOG00000042561 | *Cks1b* | 0.826 | -0.27635 | 0.003265 | 0.026139 |
| ENSRNOG00000042912 | *Mycbpap* | 2.747 | 1.457982 | 0.001659 | 0.015439 |
| ENSRNOG00000042944 | *Cenpw* | 0.606 | -0.72195 | 2.23E-08 | 7.49E-07 |
| ENSRNOG00000042980 | *Adam19* | 1.386 | 0.471013 | 3.77E-35 | 7.16E-33 |
| ENSRNOG00000043068 | *Tipin* | 1.402 | 0.487188 | 0.005586 | 0.039529 |
| ENSRNOG00000043201 | *Coq8a* | 0.381 | -1.39244 | 0.00345 | 0.027232 |
| ENSRNOG00000043288 | *Smim13* | 0.737 | -0.44032 | 0.00121 | 0.012066 |
| ENSRNOG00000043295 | *RGD1565033* | 0.727 | -0.46002 | 6.55E-05 | 0.001044 |
| ENSRNOG00000043300 | *Enho* | 2.889 | 1.53039 | 0.004357 | 0.032663 |
| ENSRNOG00000043357 | *Zfp407* | 0.755 | -0.40477 | 0.000503 | 0.005902 |
| ENSRNOG00000043364 | *Zfp362* | 0.815 | -0.29558 | 9.58E-05 | 0.001441 |
| ENSRNOG00000045548 | *Entpd7* | 0.27 | -1.88967 | 3.23E-10 | 1.4E-08 |
| ENSRNOG00000045636 | *Fasn* | 0.816 | -0.2936 | 6.08E-94 | 4.62E-91 |
| ENSRNOG00000045752 | *Rrm1* | 0.736 | -0.44224 | 4.74E-28 | 7.33E-26 |
| ENSRNOG00000045760 | *Ebna1bp2* | 1.799 | 0.847469 | 7.86E-12 | 4.1E-10 |
| ENSRNOG00000045770 | *Hira* | 1.238 | 0.308465 | 0.002234 | 0.019472 |
| ENSRNOG00000045814 | *RGD1563962* | 0.709 | -0.49699 | 0.001364 | 0.013286 |
| ENSRNOG00000045829 | *Thbs1* | 1.241 | 0.311881 | 0 | 0 |
| ENSRNOG00000045911 | *Mepce* | 0.316 | -1.66102 | 1.09E-06 | 2.72E-05 |
| ENSRNOG00000045961 | *Lyrm7* | 0.494 | -1.01805 | 0.001533 | 0.014514 |
| ENSRNOG00000045965 | *N4bp3* | 1.969 | 0.977192 | 0.002001 | 0.017874 |
| ENSRNOG00000046006 | *LOC100362783* | 5.576 | 2.479131 | 0.002985 | 0.024297 |
| ENSRNOG00000046172 | *Sf3b6* | 3.232 | 1.692447 | 0.000143 | 0.002036 |
| ENSRNOG00000046214 | *Cyp27b1* | 0.261 | -1.93558 | 0.003938 | 0.030171 |
| ENSRNOG00000046276 | *Myh3* | 1.392 | 0.47705 | 0.004549 | 0.033749 |
| ENSRNOG00000046280 | *Tceal5* | 24.158 | 4.594424 | 0.000274 | 0.003529 |
| ENSRNOG00000046445 | *Rcor3* | 1.35 | 0.433081 | 0.001714 | 0.015878 |
| ENSRNOG00000046635 | *Cdca5* | 0.546 | -0.87173 | 7.79E-08 | 2.41E-06 |
| ENSRNOG00000046791 | *Sh3rf3* | 1.315 | 0.395101 | 1.26E-07 | 3.77E-06 |
| ENSRNOG00000046834 | *C3* | 10.666 | 3.414913 | 0.001821 | 0.016687 |
| ENSRNOG00000046912 | *Nr1d2* | 0.726 | -0.46128 | 3.73E-07 | 1E-05 |
| ENSRNOG00000046990 | *Cpne1* | 0.693 | -0.52878 | 5.57E-10 | 2.35E-08 |
| ENSRNOG00000047113 | *Spsb2* | 2.902 | 1.537187 | 3.51E-07 | 9.49E-06 |
| ENSRNOG00000047115 | *Cox16* | 0.689 | -0.53671 | 1.7E-05 | 0.000315 |
| ENSRNOG00000047280 | *Cttn* | 0.66 | -0.6005 | 2.7E-10 | 1.19E-08 |
| ENSRNOG00000047300 | *Bdkrb2* | 1.566 | 0.647289 | 1.63E-18 | 1.54E-16 |
| ENSRNOG00000047314 | *Tk1* | 0.741 | -0.4327 | 2.73E-08 | 9.1E-07 |
| ENSRNOG00000047379 | *Zfp82* | 0.023 | -5.46283 | 2.12E-06 | 5E-05 |
| ENSRNOG00000047396 | *Rmnd5b* | 0.714 | -0.4869 | 4.46E-05 | 0.000739 |
| ENSRNOG00000047446 | *Foxc2* | 0.772 | -0.37392 | 6.9E-06 | 0.000143 |
| ENSRNOG00000047505 | *Tubb4a* | 1.204 | 0.26799 | 0.003653 | 0.028452 |
| ENSRNOG00000047516 | *Map3k7* | 1.305 | 0.383753 | 1.86E-07 | 5.37E-06 |
| ENSRNOG00000047526 | *Zfp526* | 0.775 | -0.36853 | 6.65E-05 | 0.001056 |
| ENSRNOG00000047719 | *LOC103689927* | 2.242 | 1.165062 | 1.12E-07 | 3.38E-06 |
| ENSRNOG00000047812 | *Triap1* | 0.484 | -1.04701 | 0.005466 | 0.038881 |
| ENSRNOG00000047854 | *RT1-DMa* | 27.926 | 4.803562 | 7.98E-05 | 0.001234 |
| ENSRNOG00000047876 | *LOC689986* | 0.32 | -1.64503 | 0.00379 | 0.029247 |
| ENSRNOG00000047967 | *Arhgef19* | 0.81 | -0.30366 | 0.00332 | 0.026442 |
| ENSRNOG00000048136 | *Senp5* | 0.046 | -4.43908 | 0.001253 | 0.012432 |
| ENSRNOG00000048166 | *Arhgap19* | 0.671 | -0.57569 | 1.05E-07 | 3.19E-06 |
| ENSRNOG00000048169 | *Tuba8* | 19.554 | 4.289382 | 0.001271 | 0.012602 |
| ENSRNOG00000048230 | *AABR07056118.1* | 8.444 | 3.077878 | 0.000178 | 0.002455 |
| ENSRNOG00000048394 | *Zfp560* | 0.734 | -0.44611 | 0.00538 | 0.038449 |
| ENSRNOG00000048450 | *Cyp4f37* | 0.757 | -0.40173 | 0.00185 | 0.016859 |
| ENSRNOG00000048470 | *Pomp* | 1.968 | 0.977093 | 0.000646 | 0.007255 |
| ENSRNOG00000048650 | *LOC103690175* | 2.322 | 1.215351 | 7.09E-05 | 0.001119 |
| ENSRNOG00000048812 | *Gpx1* | 0.754 | -0.40727 | 9.36E-11 | 4.33E-09 |
| ENSRNOG00000049028 | *C5ar2* | 5.777 | 2.53039 | 0.005063 | 0.036767 |
| ENSRNOG00000049052 | *Sgk3* | 0.581 | -0.78223 | 0.000357 | 0.004419 |
| ENSRNOG00000049097 | *Rpl7a* | 1.731 | 0.79149 | 6.48E-29 | 1.05E-26 |
| ENSRNOG00000049232 | *Tcf7l2* | 1.221 | 0.287782 | 9.06E-05 | 0.001377 |
| ENSRNOG00000049287 | *MGC94199* | 0.302 | -1.72578 | 1.66E-11 | 8.31E-10 |
| ENSRNOG00000049324 | *Tspan4* | 1.257 | 0.330204 | 0.000315 | 0.003975 |
| ENSRNOG00000049426 | *Mmab* | 0.768 | -0.38155 | 0.001542 | 0.01459 |
| ENSRNOG00000049517 | *Tnfaip3* | 1.83 | 0.871771 | 2.3E-07 | 6.45E-06 |
| ENSRNOG00000049695 | *Myh1* | 1.565 | 0.646182 | 2.4E-48 | 6.56E-46 |
| ENSRNOG00000049783 | *Rcor1* | 1.696 | 0.761801 | 1.14E-06 | 2.82E-05 |
| ENSRNOG00000050205 | *Afmid* | 0.483 | -1.04924 | 0.00402 | 0.030659 |
| ENSRNOG00000050374 | *Pigg* | 258.403 | 8.013479 | 1.6E-88 | 1.12E-85 |
| ENSRNOG00000050401 | *Vbp1* | 0.783 | -0.35301 | 1.91E-06 | 4.53E-05 |
| ENSRNOG00000050404 | *Pmepa1* | 1.23 | 0.299177 | 4.17E-17 | 3.57E-15 |
| ENSRNOG00000050482 | *Dbf4* | 0.624 | -0.68133 | 0.003051 | 0.024709 |
| ENSRNOG00000050792 | *Tnfaip6* | 1.293 | 0.371157 | 3.83E-05 | 0.000648 |
| ENSRNOG00000050806 | *Spty2d1* | 0.83 | -0.26842 | 0.007229 | 0.048331 |
| ENSRNOG00000050854 | *LOC108348101* | 0.413 | -1.27708 | 0.005863 | 0.041124 |
| ENSRNOG00000050888 | *LOC691320* | 62.59 | 5.967864 | 2.01E-09 | 7.93E-08 |
| ENSRNOG00000050946 | *Fam110a* | 0.799 | -0.32323 | 0.001839 | 0.01677 |
| ENSRNOG00000052096 | *Cox11* | 0.672 | -0.57315 | 0.000502 | 0.005888 |
| ENSRNOG00000052204 | *Tbc1d24* | 0.809 | -0.30501 | 0.000109 | 0.001609 |
| ENSRNOG00000052275 | *H2afv* | 0.811 | -0.30265 | 0.000174 | 0.002416 |
| ENSRNOG00000052613 | *Casp6* | 3.148 | 1.654629 | 4.35E-09 | 1.65E-07 |
| ENSRNOG00000052837 | *Dcun1d2* | 0.503 | -0.99014 | 0.000672 | 0.007471 |
| ENSRNOG00000053026 | *Shcbp1* | 0.639 | -0.64578 | 0.005921 | 0.041428 |
| ENSRNOG00000053047 | *Top2a* | 0.585 | -0.77443 | 1.38E-69 | 6.06E-67 |
| ENSRNOG00000053109 | *Mrpl53* | 1.954 | 0.966282 | 0.006301 | 0.043483 |
| ENSRNOG00000053272 | *Chi3l1* | 9.777 | 3.289382 | 0.003296 | 0.026353 |
| ENSRNOG00000053288 | *Ank3* | 0.79 | -0.34066 | 5.18E-06 | 0.00011 |
| ENSRNOG00000053406 | *Gramd1c* | 1.474 | 0.559303 | 0.006645 | 0.045278 |
| ENSRNOG00000053635 | *Zkscan8* | 0.818 | -0.29068 | 0.006921 | 0.046759 |
| ENSRNOG00000053735 | *Hebp2* | 1.313 | 0.392702 | 3.27E-06 | 7.25E-05 |
| ENSRNOG00000053875 | *Nacad* | 0.783 | -0.35321 | 0.006797 | 0.046088 |
| ENSRNOG00000053893 | *Opn4* | 0.261 | -1.93558 | 0.003938 | 0.030171 |
| ENSRNOG00000054218 | *Il18rap* | 1.329 | 0.410355 | 0.000201 | 0.002702 |
| ENSRNOG00000054286 | *Rrm2* | 0.588 | -0.76637 | 2.28E-37 | 4.65E-35 |
| ENSRNOG00000054297 | *Senp1* | 0.55 | -0.86364 | 2.44E-12 | 1.33E-10 |
| ENSRNOG00000054334 | *Abhd10* | 0.259 | -1.9506 | 2.32E-08 | 7.77E-07 |
| ENSRNOG00000054561 | *Isg20* | 1.398 | 0.482931 | 0.001401 | 0.01357 |
| ENSRNOG00000054563 | *Selenoh* | 0.794 | -0.33291 | 5.83E-06 | 0.000122 |
| ENSRNOG00000054576 | *Gsdmc* | 15.999 | 3.999875 | 0.004265 | 0.032132 |
| ENSRNOG00000054695 | *Calcrl* | 0.148 | -2.75501 | 0.002778 | 0.022943 |
| ENSRNOG00000054890 | *Flna* | 1.236 | 0.305186 | 0 | 0 |
| ENSRNOG00000054963 | *Rhbdd1* | 0.796 | -0.32951 | 1.86E-07 | 5.37E-06 |
| ENSRNOG00000055286 | *LOC103689986* | 0.754 | -0.40692 | 0.0002 | 0.002692 |
| ENSRNOG00000056069 | *Kif11* | 0.564 | -0.82695 | 8.98E-24 | 1.14E-21 |
| ENSRNOG00000056174 | *Pdzd4* | 0.797 | -0.3273 | 0.000166 | 0.002307 |
| ENSRNOG00000056610 | *Cog5* | 0.715 | -0.48448 | 0.000489 | 0.005768 |
| ENSRNOG00000056756 | *Actn1* | 1.213 | 0.27863 | 1.98E-64 | 7.36E-62 |
| ENSRNOG00000057009 | *Ptp4a1* | 30.486 | 4.930087 | 3.49E-05 | 0.000598 |
| ENSRNOG00000057031 | *Six3* | 10.666 | 3.414913 | 0.001821 | 0.016687 |
| ENSRNOG00000057058 | *Cd300a* | 4.222 | 2.077878 | 0.002616 | 0.021898 |
| ENSRNOG00000057153 | *Pla1a* | 1.296 | 0.37369 | 0.000687 | 0.007613 |
| ENSRNOG00000057322 | *Efna3* | 1.269 | 0.343752 | 0.000364 | 0.004494 |
| ENSRNOG00000057416 | *Zfp763* | 2.505 | 1.324715 | 0.00523 | 0.037696 |
| ENSRNOG00000057458 | *Oip5* | 0.606 | -0.72341 | 0.000248 | 0.003252 |
| ENSRNOG00000057696 | *Sbk1* | 3.978 | 1.992103 | 2.93E-26 | 4.22E-24 |
| ENSRNOG00000057795 | *Dusp13* | 0.183 | -2.45376 | 0.000128 | 0.001845 |
| ENSRNOG00000057834 | *LOC102553018* | 1.444 | 0.529888 | 1.29E-05 | 0.000246 |
| ENSRNOG00000057880 | *Myh11* | 1.523 | 0.607383 | 3.58E-19 | 3.65E-17 |
| ENSRNOG00000058007 | *Nde1* | 0.779 | -0.35942 | 3.66E-06 | 7.96E-05 |
| ENSRNOG00000058039 | *Acta2* | 1.67 | 0.739492 | 0 | 0 |
| ENSRNOG00000058186 | *Errfi1* | 1.238 | 0.308226 | 2.98E-06 | 6.71E-05 |
| ENSRNOG00000058288 | *Tcf19* | 0.684 | -0.54752 | 0.003016 | 0.024496 |
| ENSRNOG00000058372 | *Ac1576* | 0.395 | -1.33997 | 0.000318 | 0.004 |
| ENSRNOG00000058439 | *Fam50a* | 0.008 | -7.01001 | 1.79E-15 | 1.29E-13 |
| ENSRNOG00000058497 | *Fdxr* | 0.752 | -0.41212 | 0.002361 | 0.02027 |
| ENSRNOG00000058539 | *Ccnb1* | 0.539 | -0.89117 | 2.27E-17 | 1.98E-15 |
| ENSRNOG00000058555 | *RF00100* | 0.025 | -5.33997 | 5.7E-06 | 0.000119 |
| ENSRNOG00000058645 | *Tnc* | 1.771 | 0.824214 | 6.97E-08 | 2.18E-06 |
| ENSRNOG00000058739 | *Snn* | 0.731 | -0.45206 | 1.81E-17 | 1.59E-15 |
| ENSRNOG00000058790 | *Fam120b* | 0.818 | -0.28969 | 4.16E-05 | 0.000695 |
| ENSRNOG00000058904 | *AABR07040847.1* | 0.167 | -2.58509 | 0.000844 | 0.008985 |
| ENSRNOG00000059443 | *Zfp622* | 0.67 | -0.57695 | 7.83E-08 | 2.42E-06 |
| ENSRNOG00000059663 | *Rufy2* | 0.69 | -0.53524 | 1.3E-05 | 0.000248 |
| ENSRNOG00000059799 | *Ficd* | 1.321 | 0.401997 | 2.59E-09 | 1E-07 |
| ENSRNOG00000059810 | *Txnrd3* | 0.782 | -0.35404 | 0.000353 | 0.004382 |
| ENSRNOG00000059857 | *Rnd1* | 1.376 | 0.460305 | 0.000194 | 0.002619 |
| ENSRNOG00000059926 | *RF00576* | 0.781 | -0.35708 | 0.000663 | 0.007384 |
| ENSRNOG00000060047 | *Aldh18a1* | 252.725 | 7.981424 | 5.98E-30 | 1.04E-27 |
| ENSRNOG00000060066 | *Hoxa6* | 0.265 | -1.91324 | 5.11E-05 | 0.000833 |
| ENSRNOG00000060100 | *Knl1* | 0.697 | -0.52006 | 1.22E-07 | 3.64E-06 |
| ENSRNOG00000060146 | *Six5* | 0.787 | -0.34468 | 4.8E-05 | 0.00079 |
| ENSRNOG00000060185 | *LOC103690317* | 1.335 | 0.416386 | 5.28E-05 | 0.000854 |
| ENSRNOG00000060340 | *Psme4* | 0.81 | -0.30359 | 4.32E-14 | 2.85E-12 |
| ENSRNOG00000060356 | *Kif15* | 0.712 | -0.49027 | 0.00095 | 0.00986 |
| ENSRNOG00000060464 | *Plxna3* | 0.797 | -0.32733 | 3.41E-07 | 9.24E-06 |
| ENSRNOG00000060594 | *Atn1* | 1.226 | 0.29386 | 6.42E-09 | 2.39E-07 |
| ENSRNOG00000060604 | *LOC103694910* | 0.029 | -5.08844 | 3.49E-05 | 0.000599 |
| ENSRNOG00000060617 | *AC241873.1* | 0.723 | -0.46789 | 0.00246 | 0.020884 |
| ENSRNOG00000060687 | *Slc24a3* | 0.777 | -0.3643 | 2.13E-18 | 2E-16 |
| ENSRNOG00000060703 | *Troap* | 0.589 | -0.76307 | 1.55E-10 | 7.06E-09 |
| ENSRNOG00000060775 | *Lmo7* | 1.24 | 0.309821 | 8.1E-07 | 2.08E-05 |
| ENSRNOG00000061085 | *Fancd2* | 0.687 | -0.54082 | 2.95E-06 | 6.67E-05 |
| ENSRNOG00000061348 | *Fam53b* | 1.789 | 0.839086 | 7.21E-15 | 4.97E-13 |
| ENSRNOG00000061526 | *Rsph6a* | 3.022 | 1.595485 | 0.000777 | 0.008416 |
| ENSRNOG00000061995 | *Grtp1* | 1.849 | 0.88641 | 0.000488 | 0.005758 |
| ENSRNOG00000062220 | *Cenpp* | 0.499 | -1.00404 | 0.006345 | 0.043644 |

FC: Fold change, Padj. : adjusted P value.

**Table S3**. Differentially expressed genes between CCFM1149 group and the model group of A7R5 cells

| Gene_id | Gene name | FC | Log2FC | Pvalue | Padj. |
| --- | --- | --- | --- | --- | --- |
| ENSRNOG00000000413 | *Pln* | 1.757 | 0.813227 | 9.62E-07 | 5.58E-05 |
| ENSRNOG00000000487 | *Grm4* | 1.215 | 0.280394 | 0.000228 | 0.006247 |
| ENSRNOG00000000498 | *Anks1a* | 0.822 | -0.28267 | 0.000435 | 0.010449 |
| ENSRNOG00000000500 | *Scube3* | 1.969 | 0.977146 | 0.002576 | 0.039113 |
| ENSRNOG00000000504 | *Fance* | 1.284 | 0.361203 | 0.002888 | 0.042272 |
| ENSRNOG00000000818 | *Nrm* | 0.406 | -1.30114 | 0.003451 | 0.047607 |
| ENSRNOG00000000858 | *Sapcd1* | 1.42 | 0.506321 | 0.002408 | 0.037225 |
| ENSRNOG00000000925 | *Psph* | 0.762 | -0.39238 | 0.003347 | 0.046517 |
| ENSRNOG00000001058 | *Timm44* | 0.807 | -0.30928 | 0.000157 | 0.004608 |
| ENSRNOG00000001079 | *Daglb* | 1.225 | 0.29322 | 0.001244 | 0.022841 |
| ENSRNOG00000001160 | *NEWGENE_1586233* | 0.826 | -0.27503 | 0.003576 | 0.048771 |
| ENSRNOG00000001214 | *Pfkl* | 0.791 | -0.33842 | 3.84E-12 | 5.6E-10 |
| ENSRNOG00000001252 | *Chst12* | 1.245 | 0.316377 | 7.06E-05 | 0.002403 |
| ENSRNOG00000001271 | *Card6* | 0.749 | -0.4175 | 0.002847 | 0.041891 |
| ENSRNOG00000001347 | *Adam1a* | 1.696 | 0.761789 | 0.002089 | 0.033471 |
| ENSRNOG00000001416 | *Vgf* | 1.451 | 0.536675 | 0.001691 | 0.02846 |
| ENSRNOG00000001499 | *Mia* | 11.585 | 3.534149 | 0.000767 | 0.016112 |
| ENSRNOG00000001500 | *Rab4b* | 0.823 | -0.28171 | 3.75E-05 | 0.001416 |
| ENSRNOG00000001956 | *Dzip3* | 0.698 | -0.51948 | 0.000392 | 0.009582 |
| ENSRNOG00000002001 | *Itsn1* | 0.824 | -0.28001 | 1.81E-08 | 1.49E-06 |
| ENSRNOG00000002176 | *Nectin3* | 0.808 | -0.3073 | 0.000283 | 0.00737 |
| ENSRNOG00000002215 | *Mylk* | 1.209 | 0.273459 | 0.001965 | 0.031753 |
| ENSRNOG00000002831 | *Wfikkn2* | 2.095 | 1.066698 | 0.003206 | 0.045073 |
| ENSRNOG00000002946 | *Socs3* | 0.808 | -0.30771 | 3.47E-12 | 5.14E-10 |
| ENSRNOG00000002977 | *Ttc19* | 1.226 | 0.293927 | 0.002549 | 0.038764 |
| ENSRNOG00000002997 | *Slc9a3r2* | 1.256 | 0.329178 | 0.000114 | 0.003629 |
| ENSRNOG00000003066 | *Wnt9a* | 1.604 | 0.681407 | 0.002109 | 0.033689 |
| ENSRNOG00000003105 | *Kif19* | 2.806 | 1.488762 | 0.000112 | 0.003583 |
| ENSRNOG00000003224 | *Nudt16l1* | 0.13 | -2.93874 | 7.59E-15 | 1.34E-12 |
| ENSRNOG00000003870 | *C1qtnf2* | 0.694 | -0.52619 | 0.000799 | 0.016641 |
| ENSRNOG00000004425 | *Klhdc1* | 1.762 | 0.817469 | 0.003199 | 0.045091 |
| ENSRNOG00000004430 | *Cep131* | 0.818 | -0.2897 | 0.000348 | 0.008721 |
| ENSRNOG00000004757 | *Tmem158* | 1.238 | 0.308122 | 0.0007 | 0.015017 |
| ENSRNOG00000004789 | *Utp25* | 0.787 | -0.34496 | 0.000283 | 0.007369 |
| ENSRNOG00000005037 | *Kif18a* | 0.636 | -0.65288 | 0.000718 | 0.015235 |
| ENSRNOG00000005151 | *Dync2li1* | 0.719 | -0.47605 | 0.00312 | 0.044384 |
| ENSRNOG00000005711 | *Ptprd* | 0.83 | -0.26931 | 0.003369 | 0.046698 |
| ENSRNOG00000005747 | *Il27ra* | 2.23 | 1.157309 | 0.002808 | 0.041498 |
| ENSRNOG00000005868 | *Ttc21b* | 0.803 | -0.31582 | 0.00064 | 0.014048 |
| ENSRNOG00000005929 | *Them6* | 1.396 | 0.481432 | 0.001367 | 0.024296 |
| ENSRNOG00000006619 | *Dnajc9* | 0.777 | -0.36479 | 0.000157 | 0.004599 |
| ENSRNOG00000006684 | *Zfp317* | 0.765 | -0.38632 | 0.000103 | 0.003353 |
| ENSRNOG00000006700 | *Wdyhv1* | 0.556 | -0.84584 | 0.000792 | 0.016509 |
| ENSRNOG00000006740 | *Castor1* | 0.475 | -1.07472 | 4.63E-05 | 0.001685 |
| ENSRNOG00000006827 | *Tmem198b* | 0.765 | -0.38676 | 0.003467 | 0.047669 |
| ENSRNOG00000006966 | *Nfia* | 0.735 | -0.44463 | 0.002112 | 0.033675 |
| ENSRNOG00000007206 | *LOC361016* | 0.448 | -1.15837 | 0.001442 | 0.025141 |
| ENSRNOG00000007224 | *Bmt2* | 0.747 | -0.42069 | 0.001128 | 0.021286 |
| ENSRNOG00000007319 | *Trib3* | 0.803 | -0.31621 | 0.000639 | 0.014049 |
| ENSRNOG00000007324 | *Plxna2* | 3.666 | 1.874052 | 0.001456 | 0.025255 |
| ENSRNOG00000007398 | *Zfp691* | 1.793 | 0.842344 | 0.000922 | 0.018376 |
| ENSRNOG00000007430 | *Slx4ip* | 0.726 | -0.46121 | 0.000224 | 0.006183 |
| ENSRNOG00000007541 | *Fhl3* | 1.206 | 0.27057 | 0.000569 | 0.012827 |
| ENSRNOG00000007663 | *Rps6* | 0.659 | -0.60221 | 4.32E-05 | 0.00159 |
| ENSRNOG00000007817 | *Kctd6* | 1.935 | 0.952055 | 0.003455 | 0.047577 |
| ENSRNOG00000007922 | *Cldn19* | 1.216 | 0.281556 | 0.002073 | 0.033305 |
| ENSRNOG00000008010 | *Hoxb5* | 0.767 | -0.38343 | 3.11E-05 | 0.001203 |
| ENSRNOG00000008059 | *Rnf17* | 1.932 | 0.950001 | 0.000639 | 0.01404 |
| ENSRNOG00000008086 | *Dpf3* | 13.746 | 3.780943 | 0.000264 | 0.006978 |
| ENSRNOG00000008118 | *Sync* | 0.818 | -0.28994 | 0.003433 | 0.047399 |
| ENSRNOG00000008351 | *RGD1308117* | 1.939 | 0.955244 | 0.000872 | 0.017804 |
| ENSRNOG00000008432 | *Slc22a5* | 1.305 | 0.384543 | 0.000936 | 0.018572 |
| ENSRNOG00000008618 | *Tex10* | 0.784 | -0.35159 | 0.000161 | 0.004692 |
| ENSRNOG00000008738 | *Tp53i11* | 1.245 | 0.316571 | 0.000288 | 0.007435 |
| ENSRNOG00000008861 | *AABR07047011.1* | 0.683 | -0.54966 | 0.002809 | 0.041486 |
| ENSRNOG00000008873 | *Ino80b* | 1.348 | 0.430692 | 0.002677 | 0.040133 |
| ENSRNOG00000009014 | *Slc35f2* | 0.344 | -1.54099 | 0.000184 | 0.005231 |
| ENSRNOG00000009144 | *Lad1* | 2.062 | 1.043977 | 0.002129 | 0.033892 |
| ENSRNOG00000009173 | *Smad6* | 1.222 | 0.28909 | 0.000286 | 0.00743 |
| ENSRNOG00000009599 | *Rwdd2a* | 1.892 | 0.919856 | 0.002609 | 0.039538 |
| ENSRNOG00000009640 | *Psmf1* | 1.223 | 0.290617 | 0.000875 | 0.0178 |
| ENSRNOG00000010407 | *Pex5* | 0.812 | -0.30002 | 0.002361 | 0.036638 |
| ENSRNOG00000010468 | *Elovl6* | 0.671 | -0.5765 | 0.001331 | 0.023809 |
| ENSRNOG00000010720 | *Mast4* | 1.241 | 0.311364 | 6.71E-05 | 0.002294 |
| ENSRNOG00000010983 | *Otog* | 1.227 | 0.295139 | 0.003266 | 0.045691 |
| ENSRNOG00000010994 | *Has1* | 0.115 | -3.12595 | 0.000267 | 0.007015 |
| ENSRNOG00000011000 | *Rims1* | 1.438 | 0.523995 | 0.001426 | 0.024914 |
| ENSRNOG00000011053 | *Cyp2u1* | 0.822 | -0.283 | 0.001238 | 0.022804 |
| ENSRNOG00000011096 | *Hmgb3* | 0.68 | -0.5563 | 3.79E-10 | 4.12E-08 |
| ENSRNOG00000011227 | *Atp1b2* | 1.268 | 0.342569 | 0.000917 | 0.018349 |
| ENSRNOG00000011459 | *Rhbdf2* | 1.325 | 0.406322 | 0.000103 | 0.003355 |
| ENSRNOG00000011552 | *Mon1b* | 0.611 | -0.71091 | 0.000817 | 0.01687 |
| ENSRNOG00000011621 | *Hnrnpc* | 1.321 | 0.401982 | 1.98E-07 | 1.36E-05 |
| ENSRNOG00000011692 | *Dkk1* | 14.338 | 3.841759 | 3.01E-75 | 6.31E-72 |
| ENSRNOG00000011800 | *F3* | 1.308 | 0.386823 | 7.58E-10 | 7.99E-08 |
| ENSRNOG00000011908 | *Asxl2* | 0.799 | -0.32459 | 2.18E-11 | 2.86E-09 |
| ENSRNOG00000011964 | *Abcd4* | 1.233 | 0.302022 | 0.00053 | 0.01211 |
| ENSRNOG00000012151 | *Itpripl1* | 0.092 | -3.44788 | 0.002642 | 0.03986 |
| ENSRNOG00000012287 | *Slc35e1* | 3.155 | 1.6575 | 0.000873 | 0.017791 |
| ENSRNOG00000012307 | *Mybpc3* | 3.131 | 1.646642 | 0.000143 | 0.004312 |
| ENSRNOG00000012343 | *Pdp2* | 0.79 | -0.3393 | 0.000661 | 0.01439 |
| ENSRNOG00000012729 | *Mfsd8* | 0.637 | -0.65048 | 0.000192 | 0.005404 |
| ENSRNOG00000012851 | *Dnajc5b* | 0.051 | -4.29587 | 0.002154 | 0.034124 |
| ENSRNOG00000012920 | *Col9a1* | 32.99 | 5.043977 | 1.84E-05 | 0.000777 |
| ENSRNOG00000012962 | *Nudt16* | 0.778 | -0.36255 | 0.001263 | 0.022914 |
| ENSRNOG00000013069 | *Sapcd2* | 0.788 | -0.34334 | 0.002958 | 0.042854 |
| ENSRNOG00000013314 | *Avl9* | 0.792 | -0.33583 | 0.001121 | 0.021229 |
| ENSRNOG00000013323 | *Rnf217* | 0.766 | -0.38539 | 0.000297 | 0.007585 |
| ENSRNOG00000013397 | *Foxo1* | 1.506 | 0.59026 | 0.002671 | 0.040106 |
| ENSRNOG00000013515 | *Ptpru* | 1.974 | 0.980968 | 0.002964 | 0.042867 |
| ENSRNOG00000013541 | *Sh2d4a* | 1.386 | 0.47142 | 0.000368 | 0.00912 |
| ENSRNOG00000013653 | *Pdlim7* | 1.203 | 0.266343 | 1.33E-12 | 2.06E-10 |
| ENSRNOG00000013656 | *Lpar1* | 0.083 | -3.58538 | 0.001365 | 0.024295 |
| ENSRNOG00000013729 | *RGD1306271* | 0.776 | -0.36623 | 1.08E-05 | 0.000483 |
| ENSRNOG00000013898 | *Cdca4* | 0.775 | -0.36715 | 7.96E-06 | 0.00037 |
| ENSRNOG00000013946 | *Rnf149* | 0.76 | -0.3968 | 0.000704 | 0.015094 |
| ENSRNOG00000014021 | *Matn4* | 1.858 | 0.893952 | 0.001697 | 0.028535 |
| ENSRNOG00000014027 | *RGD1304728* | 0.771 | -0.37578 | 5.54E-05 | 0.001942 |
| ENSRNOG00000014041 | *Cep78* | 0.715 | -0.4831 | 0.000773 | 0.016208 |
| ENSRNOG00000014089 | *Map3k2* | 0.727 | -0.45913 | 6.38E-05 | 0.0022 |
| ENSRNOG00000014314 | *Slc39a4* | 4.124 | 2.043977 | 0.003505 | 0.048105 |
| ENSRNOG00000014480 | *Sys1* | 1.257 | 0.329797 | 0.000166 | 0.004797 |
| ENSRNOG00000014551 | *Ccnj* | 80.002 | 6.321962 | 2.08E-11 | 2.74E-09 |
| ENSRNOG00000014568 | *Ndufb10* | 1.201 | 0.264314 | 0.000667 | 0.014433 |
| ENSRNOG00000014684 | *Npr1* | 1.588 | 0.667123 | 5.29E-05 | 0.001883 |
| ENSRNOG00000014956 | *Slc11a1* | 6.415 | 2.681407 | 0.000212 | 0.00589 |
| ENSRNOG00000014987 | *Mdfi* | 2.749 | 1.459015 | 0.000266 | 0.00701 |
| ENSRNOG00000015035 | *Myo7b* | 1.727 | 0.7883 | 9.14E-21 | 2.89E-18 |
| ENSRNOG00000015078 | *Ifitm3* | 1.376 | 0.460829 | 4.65E-05 | 0.001689 |
| ENSRNOG00000015275 | *Ska1* | 0.602 | -0.73167 | 0.000132 | 0.00406 |
| ENSRNOG00000015440 | *Wrn* | 0.773 | -0.37228 | 0.000372 | 0.009157 |
| ENSRNOG00000015529 | *Cdca3* | 0.742 | -0.43104 | 1.06E-07 | 7.67E-06 |
| ENSRNOG00000015591 | *Cndp2* | 1.221 | 0.287861 | 0.000239 | 0.00648 |
| ENSRNOG00000015818 | *Tprkb* | 26.796 | 4.743924 | 0.000131 | 0.004043 |
| ENSRNOG00000015969 | *Rpf1* | 1.276 | 0.351301 | 0.001809 | 0.029933 |
| ENSRNOG00000016021 | *Lims2* | 1.235 | 0.305047 | 0.000247 | 0.006676 |
| ENSRNOG00000016303 | *Zfp236* | 0.795 | -0.33153 | 0.00211 | 0.033684 |
| ENSRNOG00000016338 | *Fam92a* | 0.68 | -0.55693 | 0.000628 | 0.013871 |
| ENSRNOG00000016368 | *Ppp1r14c* | 0.827 | -0.27369 | 2.1E-06 | 0.000112 |
| ENSRNOG00000016405 | *Pcsk4* | 1.398 | 0.48371 | 2.96E-05 | 0.001151 |
| ENSRNOG00000016454 | *Nasp* | 0.765 | -0.38608 | 5.47E-05 | 0.00193 |
| ENSRNOG00000016581 | *Serpinb1a* | 1.214 | 0.279885 | 0.000873 | 0.017778 |
| ENSRNOG00000016603 | *Rtn2* | 1.493 | 0.57792 | 0.001615 | 0.0274 |
| ENSRNOG00000016656 | *Nkx2-3* | 1.413 | 0.498761 | 0.000266 | 0.007011 |
| ENSRNOG00000016767 | *Ggps1* | 0.794 | -0.33355 | 0.003035 | 0.043744 |
| ENSRNOG00000016875 | *Cbx7* | 1.345 | 0.427744 | 0.002536 | 0.03867 |
| ENSRNOG00000017001 | *Zfp652* | 0.802 | -0.31911 | 0.002504 | 0.038367 |
| ENSRNOG00000017075 | *Slc35e2b* | 0.693 | -0.52984 | 0.001487 | 0.02562 |
| ENSRNOG00000017136 | *Syt17* | 1.524 | 0.608071 | 0.000802 | 0.016673 |
| ENSRNOG00000017137 | *Eef1akmt2* | 0.746 | -0.42211 | 5.45E-07 | 3.36E-05 |
| ENSRNOG00000017149 | *Fam131b* | 1.608 | 0.685443 | 9.34E-05 | 0.003076 |
| ENSRNOG00000017538 | *Lrrc27* | 0.434 | -1.20395 | 0.002535 | 0.038687 |
| ENSRNOG00000018297 | *Ocln* | 1.677 | 0.746045 | 0.00195 | 0.031577 |
| ENSRNOG00000018515 | *Det1* | 1.397 | 0.482007 | 0.000715 | 0.015235 |
| ENSRNOG00000018516 | *Impa2* | 1.393 | 0.477825 | 0.00337 | 0.046686 |
| ENSRNOG00000018547 | *Mrpl46* | 1.3 | 0.378437 | 0.000875 | 0.017762 |
| ENSRNOG00000018886 | *Prxl2c* | 1.23 | 0.298309 | 0.003181 | 0.04492 |
| ENSRNOG00000018929 | *Kif20b* | 0.828 | -0.27161 | 0.00088 | 0.017792 |
| ENSRNOG00000019147 | *Stom* | 1.375 | 0.459015 | 0.000158 | 0.004629 |
| ENSRNOG00000019208 | *P2rx5* | 0.792 | -0.33596 | 0.00137 | 0.024278 |
| ENSRNOG00000019330 | *Procr* | 1.36 | 0.443659 | 3.99E-13 | 6.56E-11 |
| ENSRNOG00000019351 | *Chid1* | 1.89 | 0.918507 | 0.000355 | 0.008868 |
| ENSRNOG00000019478 | *Irf9* | 0.583 | -0.77825 | 0.000116 | 0.003665 |
| ENSRNOG00000019737 | *Sema4a* | 1.347 | 0.429914 | 0.000627 | 0.013871 |
| ENSRNOG00000019982 | *Ethe1* | 1.287 | 0.363858 | 0.000166 | 0.004797 |
| ENSRNOG00000020263 | *Atp1a3* | 3.156 | 1.658324 | 0.000889 | 0.01789 |
| ENSRNOG00000020372 | *Hdac4* | 0.798 | -0.32625 | 2.15E-05 | 0.000879 |
| ENSRNOG00000020374 | *Tmem8a* | 1.372 | 0.456733 | 6.61E-07 | 4E-05 |
| ENSRNOG00000020389 | *Capn12* | 7.336 | 2.874945 | 0.000751 | 0.015832 |
| ENSRNOG00000020762 | *Zfp260* | 1.455 | 0.541382 | 2.4E-11 | 3.12E-09 |
| ENSRNOG00000020781 | *Tbcb* | 2.628 | 1.394049 | 3.11E-06 | 0.00016 |
| ENSRNOG00000020829 | *Them4* | 1.795 | 0.843679 | 0.000697 | 0.015 |
| ENSRNOG00000020941 | *Psenen* | 1.202 | 0.265561 | 0.001648 | 0.027841 |
| ENSRNOG00000020952 | *Cgn* | 1.461 | 0.546758 | 0.002465 | 0.037971 |
| ENSRNOG00000021153 | *Fkbp2* | 1.299 | 0.377926 | 3.23E-05 | 0.001243 |
| ENSRNOG00000021234 | *Slc4a11* | 1.55 | 0.632347 | 0.000607 | 0.013635 |
| ENSRNOG00000021244 | *Hspa12b* | 1.424 | 0.510362 | 0.00015 | 0.004492 |
| ENSRNOG00000021536 | *Plxdc1* | 1.511 | 0.595866 | 0.000127 | 0.003943 |
| ENSRNOG00000021856 | *Lat2* | 1.477 | 0.562966 | 0.000807 | 0.016748 |
| ENSRNOG00000022082 | *Dusp11* | 0.012 | -6.34628 | 1.01E-10 | 1.17E-08 |
| ENSRNOG00000022391 | *Zfp711* | 0.783 | -0.35206 | 0.002808 | 0.041532 |
| ENSRNOG00000022597 | *Cenpj* | 0.665 | -0.58768 | 2.43E-06 | 0.000127 |
| ENSRNOG00000022603 | *Astl* | 5.193 | 2.376553 | 0.001844 | 0.030328 |
| ENSRNOG00000022723 | *RGD1562029* | 2.083 | 1.058477 | 0.002798 | 0.041493 |
| ENSRNOG00000022845 | *Cep70* | 1.869 | 0.902622 | 0.000184 | 0.005237 |
| ENSRNOG00000022929 | *Mtmr12* | 0.804 | -0.3145 | 0.000137 | 0.004148 |
| ENSRNOG00000022946 | *Slc22a3* | 8.248 | 3.043977 | 0.000256 | 0.006831 |
| ENSRNOG00000023016 | *Magea9* | 1.208 | 0.272199 | 0.00148 | 0.025543 |
| ENSRNOG00000023023 | *Trpt1* | 1.956 | 0.968241 | 0.000784 | 0.016412 |
| ENSRNOG00000023035 | *Smim8* | 1.549 | 0.631482 | 0.001212 | 0.022416 |
| ENSRNOG00000023230 | *RGD1307621* | 0.501 | -0.99721 | 0.000271 | 0.007101 |
| ENSRNOG00000023318 | *Tigd3* | 2.851 | 1.511482 | 0.003147 | 0.044503 |
| ENSRNOG00000023403 | *Gtpbp3* | 0.673 | -0.57201 | 0.000371 | 0.009159 |
| ENSRNOG00000023410 | *Apol9a* | 0.657 | -0.60628 | 0.003639 | 0.049459 |
| ENSRNOG00000023538 | *Aldh5a1* | 2.495 | 1.318837 | 0.000425 | 0.010268 |
| ENSRNOG00000023686 | *Upk3b* | 1.812 | 0.857933 | 0.000818 | 0.016856 |
| ENSRNOG00000023920 | *Zfp791* | 19.648 | 4.296285 | 0.001348 | 0.024087 |
| ENSRNOG00000024288 | *Etv2* | 3.849 | 1.944442 | 0.002272 | 0.03542 |
| ENSRNOG00000024338 | *LOC690276* | 1.526 | 0.610084 | 0.001291 | 0.023295 |
| ENSRNOG00000024505 | *Ppp1r35* | 0.021 | -5.58996 | 5.85E-07 | 3.58E-05 |
| ENSRNOG00000024635 | *Cramp1* | 0.782 | -0.35554 | 0.000475 | 0.011166 |
| ENSRNOG00000024730 | *Ppm1e* | 0.82 | -0.28554 | 0.00265 | 0.039865 |
| ENSRNOG00000024889 | *Snip1* | 1.947 | 0.960952 | 2.77E-05 | 0.001099 |
| ENSRNOG00000025059 | *Nxph4* | 2.062 | 1.043977 | 0.000169 | 0.004848 |
| ENSRNOG00000025100 | *Ikbke* | 0.781 | -0.35685 | 5.01E-06 | 0.000249 |
| ENSRNOG00000025184 | *Prss35* | 1.572 | 0.652838 | 0.000215 | 0.005956 |
| ENSRNOG00000025269 | *Slc25a44* | 0.779 | -0.36102 | 0.000503 | 0.011581 |
| ENSRNOG00000026235 | *Hk3* | 0.282 | -1.82639 | 0.000671 | 0.014493 |
| ENSRNOG00000026672 | *MGC94199* | 3.015 | 1.592296 | 1.49E-15 | 2.81E-13 |
| ENSRNOG00000027002 | *NEWGENE_1310139* | 0.567 | -0.81877 | 1.43E-13 | 2.39E-11 |
| ENSRNOG00000027089 | *Ell2* | 0.771 | -0.37523 | 0.00128 | 0.023109 |
| ENSRNOG00000027220 | *Pcdhgb8* | 1.25 | 0.321697 | 0.000611 | 0.0137 |
| ENSRNOG00000027271 | *RGD1359290* | 0.707 | -0.5 | 0.001825 | 0.030136 |
| ENSRNOG00000027430 | *Ikzf2* | 0.407 | -1.29587 | 3.02E-06 | 0.000156 |
| ENSRNOG00000027456 | *Cdc42bpg* | 1.366 | 0.450402 | 0.000259 | 0.006902 |
| ENSRNOG00000027540 | *Fam102b* | 0.62 | -0.68938 | 0.000406 | 0.009871 |
| ENSRNOG00000027593 | *Ndufv3* | 2.64 | 1.400511 | 4.99E-05 | 0.001788 |
| ENSRNOG00000027736 | *Cnn1* | 1.219 | 0.286072 | 2.83E-09 | 2.75E-07 |
| ENSRNOG00000027770 | *Trpm3* | 1.959 | 0.970268 | 0.001947 | 0.031561 |
| ENSRNOG00000027906 | *Ankrd11* | 0.763 | -0.38943 | 1.35E-29 | 7.06E-27 |
| ENSRNOG00000028302 | *Smarcb1* | 1.338 | 0.419629 | 9.35E-06 | 0.000423 |
| ENSRNOG00000028404 | *Ppp1r1b* | 1.444 | 0.530441 | 5.54E-06 | 0.000274 |
| ENSRNOG00000028659 | *Szt2* | 0.717 | -0.48076 | 1.29E-06 | 7.24E-05 |
| ENSRNOG00000028904 | *Vps9d1* | 0.74 | -0.43509 | 0.000205 | 0.005736 |
| ENSRNOG00000029055 | *Ttk* | 0.736 | -0.44135 | 0.000193 | 0.005417 |
| ENSRNOG00000029738 | *Diras1* | 2.199 | 1.137087 | 0.001911 | 0.03122 |
| ENSRNOG00000029773 | *Atm* | 0.789 | -0.34231 | 1.71E-06 | 9.38E-05 |
| ENSRNOG00000030452 | *Rimbp3* | 1.693 | 0.759881 | 0.003214 | 0.045112 |
| ENSRNOG00000030463 | *Faim* | 0.084 | -3.57359 | 1.85E-07 | 1.28E-05 |
| ENSRNOG00000031315 | *LOC108348287* | 1.225 | 0.292672 | 1.92E-05 | 0.000799 |
| ENSRNOG00000031495 | *Tmem170b* | 0.596 | -0.74704 | 3.21E-07 | 2.12E-05 |
| ENSRNOG00000031660 | *LOC500948* | 4.054 | 2.019501 | 0.000894 | 0.017961 |
| ENSRNOG00000031834 | *Nkain4* | 1.646 | 0.719078 | 0.002702 | 0.040433 |
| ENSRNOG00000031851 | *Ndufa4l2* | 0.038 | -4.71091 | 0.0003 | 0.007649 |
| ENSRNOG00000032303 | *LOC108349682* | 1.558 | 0.639973 | 0.000127 | 0.003953 |
| ENSRNOG00000032439 | *Rsl1d1* | 4.805 | 2.26442 | 1.34E-20 | 4.08E-18 |
| ENSRNOG00000032618 | *Mst1r* | 12.83 | 3.681407 | 0.00048 | 0.011226 |
| ENSRNOG00000032778 | *Bub1* | 0.812 | -0.29997 | 5.35E-05 | 0.001896 |
| ENSRNOG00000032902 | *Ybx1-ps3* | 1.257 | 0.330202 | 0.001065 | 0.020468 |
| ENSRNOG00000033299 | *Mt-atp8* | 1.287 | 0.363971 | 1.96E-35 | 1.42E-32 |
| ENSRNOG00000033741 | *Ankrd34a* | 1.292 | 0.369294 | 0.000731 | 0.015459 |
| ENSRNOG00000034130 | *LOC108349189* | 4.599 | 2.201412 | 1.19E-31 | 7.11E-29 |
| ENSRNOG00000035631 | *Mir221* | 1.861 | 0.89642 | 0.002992 | 0.043192 |
| ENSRNOG00000036572 | *Sfxn4* | 0.626 | -0.67586 | 4.58E-05 | 0.001681 |
| ENSRNOG00000036697 | *Mafg* | 0.778 | -0.36288 | 0.000239 | 0.006488 |
| ENSRNOG00000037080 | *Adamts17* | 2.679 | 1.42154 | 0.001015 | 0.019714 |
| ENSRNOG00000037221 | *Tipinl1* | 1.308 | 0.386954 | 0.002172 | 0.034275 |
| ENSRNOG00000037275 | *Tlcd2* | 2.802 | 1.486457 | 0.001012 | 0.019685 |
| ENSRNOG00000037556 | *Zfp420* | 0.175 | -2.51406 | 1.31E-07 | 9.3E-06 |
| ENSRNOG00000037984 | *Sccpdh* | 0.439 | -1.18691 | 5.2E-22 | 1.86E-19 |
| ENSRNOG00000038166 | *Ptgr2* | 1.676 | 0.74481 | 1.42E-08 | 1.2E-06 |
| ENSRNOG00000038436 | *RGD1307100* | 0.805 | -0.31379 | 2.66E-14 | 4.59E-12 |
| ENSRNOG00000038902 | *RGD1565641* | 1.369 | 0.453279 | 0.001447 | 0.025179 |
| ENSRNOG00000038970 | *AABR07037410.1* | 1.359 | 0.442193 | 3.28E-07 | 2.15E-05 |
| ENSRNOG00000039924 | *Triqk* | 1.666 | 0.736549 | 0.001869 | 0.030674 |
| ENSRNOG00000040300 | *Raet1e* | 1.495 | 0.580053 | 0.000152 | 0.004521 |
| ENSRNOG00000042118 | *Cplane1* | 0.817 | -0.29164 | 0.00027 | 0.007092 |
| ENSRNOG00000042679 | *AABR07006860.1* | 0.811 | -0.30136 | 7.27E-06 | 0.000345 |
| ENSRNOG00000042886 | *Rps28* | 1.272 | 0.346661 | 1.98E-10 | 2.21E-08 |
| ENSRNOG00000042944 | *Cenpw* | 0.781 | -0.35609 | 0.003547 | 0.048531 |
| ENSRNOG00000043342 | *LOC290595* | 1.624 | 0.699507 | 6.51E-09 | 5.93E-07 |
| ENSRNOG00000043357 | *Zfp407* | 0.735 | -0.44463 | 0.000167 | 0.004797 |
| ENSRNOG00000045545 | *Eid2b* | 0.218 | -2.19634 | 0.000566 | 0.012777 |
| ENSRNOG00000045548 | *Entpd7* | 0.417 | -1.26307 | 3.37E-06 | 0.000172 |
| ENSRNOG00000045670 | *Tnfsf12* | 1.217 | 0.282938 | 0.001243 | 0.022848 |
| ENSRNOG00000045679 | *Apoa1* | 4.658 | 2.219591 | 0.001081 | 0.020667 |
| ENSRNOG00000045760 | *Ebna1bp2* | 1.464 | 0.54944 | 2.94E-05 | 0.001147 |
| ENSRNOG00000045814 | *RGD1563962* | 0.629 | -0.66846 | 3.36E-05 | 0.001289 |
| ENSRNOG00000045911 | *Mepce* | 0.29 | -1.78363 | 3.7E-07 | 2.39E-05 |
| ENSRNOG00000045961 | *Lyrm7* | 0.526 | -0.92664 | 0.003655 | 0.049605 |
| ENSRNOG00000046242 | *Klf7* | 0.741 | -0.43205 | 0.000921 | 0.018397 |
| ENSRNOG00000046382 | *Gatd1* | 1.406 | 0.491771 | 0.000123 | 0.003831 |
| ENSRNOG00000046414 | *Vwa5a* | 0.313 | -1.67572 | 2.15E-92 | 9E-89 |
| ENSRNOG00000046700 | *Mettl27* | 2.286 | 1.192519 | 0.000619 | 0.013773 |
| ENSRNOG00000046727 | *Abcc2* | 3.093 | 1.62894 | 0.002197 | 0.03451 |
| ENSRNOG00000046867 | *Zfp260* | 0.786 | -0.34669 | 3.53E-07 | 2.29E-05 |
| ENSRNOG00000047040 | *Lhb* | 4.949 | 2.307012 | 0.000115 | 0.00365 |
| ENSRNOG00000047113 | *Spsb2* | 2.046 | 1.032953 | 0.001863 | 0.030604 |
| ENSRNOG00000047115 | *Cox16* | 0.68 | -0.55534 | 1.08E-05 | 0.000483 |
| ENSRNOG00000047165 | *Fgd1* | 0.74 | -0.43413 | 2.82E-05 | 0.001115 |
| ENSRNOG00000047300 | *Bdkrb2* | 1.235 | 0.30404 | 0.000116 | 0.003659 |
| ENSRNOG00000047396 | *Rmnd5b* | 0.48 | -1.05775 | 1.6E-15 | 2.99E-13 |
| ENSRNOG00000047516 | *Map3k7* | 1.237 | 0.306508 | 4.59E-05 | 0.00168 |
| ENSRNOG00000047635 | *Tmem178b* | 0.701 | -0.5116 | 0.002821 | 0.041612 |
| ENSRNOG00000047719 | *LOC103689927* | 3.848 | 1.944062 | 9.09E-24 | 3.63E-21 |
| ENSRNOG00000047854 | *RT1-DMa* | 46.553 | 5.540809 | 2.91E-07 | 1.94E-05 |
| ENSRNOG00000048136 | *Senp5* | 0.048 | -4.39498 | 0.001407 | 0.024765 |
| ENSRNOG00000048172 | *Rac3* | 0.33 | -1.59988 | 0.002378 | 0.03687 |
| ENSRNOG00000048195 | *Nudt13* | 0.437 | -1.19306 | 0.001257 | 0.022883 |
| ENSRNOG00000048470 | *Pomp* | 2.001 | 1.000378 | 0.000503 | 0.011575 |
| ENSRNOG00000048561 | *Hprt1* | 2.296 | 1.199332 | 1.07E-09 | 1.11E-07 |
| ENSRNOG00000048650 | *LOC103690175* | 5.207 | 2.380346 | 3.49E-21 | 1.17E-18 |
| ENSRNOG00000049062 | *Trappc2b* | 2.816 | 1.493892 | 1.56E-06 | 8.68E-05 |
| ENSRNOG00000049097 | *Rpl7a* | 1.981 | 0.986235 | 3.53E-46 | 3.12E-43 |
| ENSRNOG00000049232 | *Tcf7l2* | 1.208 | 0.273057 | 0.000238 | 0.006481 |
| ENSRNOG00000049287 | *MGC94199* | 0.09 | -3.47541 | 3E-23 | 1.14E-20 |
| ENSRNOG00000049593 | *Wbp11* | 0.697 | -0.5199 | 4.78E-11 | 5.81E-09 |
| ENSRNOG00000049686 | *Mrpl40* | 1.276 | 0.351794 | 0.003554 | 0.048551 |
| ENSRNOG00000049708 | *Wdr62* | 25.348 | 4.663782 | 0.000208 | 0.005813 |
| ENSRNOG00000050374 | *Pigg* | 219.694 | 7.77935 | 5.7E-76 | 1.37E-72 |
| ENSRNOG00000050482 | *Dbf4* | 1.884 | 0.913423 | 2.06E-07 | 1.4E-05 |
| ENSRNOG00000050497 | *Zfp7* | 0.192 | -2.38074 | 9.79E-07 | 5.66E-05 |
| ENSRNOG00000050792 | *Tnfaip6* | 1.277 | 0.353146 | 0.000107 | 0.003457 |
| ENSRNOG00000050794 | *Pdlim4* | 1.206 | 0.269981 | 0.000256 | 0.006834 |
| ENSRNOG00000050828 | *Vkorc1* | 1.288 | 0.365093 | 0.00251 | 0.038413 |
| ENSRNOG00000051671 | *Herc1* | 0.83 | -0.26817 | 6.68E-09 | 6.05E-07 |
| ENSRNOG00000052157 | *Nav3* | 0.757 | -0.40158 | 0.001169 | 0.02192 |
| ENSRNOG00000052226 | *LOC108348144* | 1.2 | 0.263219 | 1.17E-05 | 0.000518 |
| ENSRNOG00000052613 | *Casp6* | 0.016 | -5.92244 | 2.06E-08 | 1.67E-06 |
| ENSRNOG00000052804 | *Hoxa5* | 28.115 | 4.813279 | 8.57E-05 | 0.002861 |
| ENSRNOG00000053269 | *Tmem106c* | 1.218 | 0.284037 | 0.001953 | 0.031596 |
| ENSRNOG00000053362 | *Gabarapl1* | 1.209 | 0.274203 | 3.79E-07 | 2.44E-05 |
| ENSRNOG00000053541 | *Kansl2* | 0.705 | -0.50406 | 1.73E-10 | 1.98E-08 |
| ENSRNOG00000053560 | *Rhou* | 3.36 | 1.748522 | 0.003658 | 0.049602 |
| ENSRNOG00000053735 | *Hebp2* | 1.237 | 0.307012 | 0.000377 | 0.009276 |
| ENSRNOG00000054286 | *Rrm2* | 0.808 | -0.30747 | 3.69E-08 | 2.89E-06 |
| ENSRNOG00000054297 | *Senp1* | 0.757 | -0.40231 | 0.000453 | 0.010806 |
| ENSRNOG00000054334 | *Abhd10* | 0.494 | -1.01649 | 0.000685 | 0.014782 |
| ENSRNOG00000055647 | *Rbfa* | 0.817 | -0.29217 | 0.002126 | 0.033877 |
| ENSRNOG00000056458 | *Cep290* | 0.687 | -0.54258 | 0.00193 | 0.031349 |
| ENSRNOG00000056740 | *Cylc1* | 0.36 | -1.47228 | 0.000287 | 0.007423 |
| ENSRNOG00000056898 | *RF00544* | 1.607 | 0.684782 | 0.002543 | 0.038707 |
| ENSRNOG00000057009 | *Ptp4a1* | 62.205 | 5.958967 | 3.02E-09 | 2.92E-07 |
| ENSRNOG00000057347 | *Cebpb* | 1.493 | 0.578498 | 1.28E-11 | 1.75E-09 |
| ENSRNOG00000057416 | *Zfp763* | 3.166 | 1.662548 | 0.000228 | 0.006242 |
| ENSRNOG00000057464 | *Fmr1* | 0.787 | -0.34602 | 6.87E-08 | 5.19E-06 |
| ENSRNOG00000057696 | *Sbk1* | 7.178 | 2.843585 | 8.42E-68 | 1.57E-64 |
| ENSRNOG00000057834 | *LOC102553018* | 1.36 | 0.443702 | 0.000357 | 0.008874 |
| ENSRNOG00000058039 | *Acta2* | 1.264 | 0.338507 | 3.1E-155 | 2.6E-151 |
| ENSRNOG00000058439 | *Fam50a* | 2.061 | 1.043306 | 2.12E-06 | 0.000113 |
| ENSRNOG00000058645 | *Tnc* | 1.411 | 0.496685 | 0.002324 | 0.036128 |
| ENSRNOG00000058920 | *Apopt1* | 1.697 | 0.76297 | 3.61E-05 | 0.001371 |
| ENSRNOG00000059443 | *Zfp622* | 0.791 | -0.33839 | 0.001126 | 0.021305 |
| ENSRNOG00000059663 | *Rufy2* | 0.777 | -0.36331 | 0.002488 | 0.03826 |
| ENSRNOG00000059776 | *Tnks2* | 0.002 | -8.91757 | 1.11E-45 | 9.27E-43 |
| ENSRNOG00000059799 | *Ficd* | 1.24 | 0.310152 | 7.25E-06 | 0.000345 |
| ENSRNOG00000059926 | *RF00576* | 0.785 | -0.35013 | 0.000915 | 0.018326 |
| ENSRNOG00000060066 | *Hoxa6* | 2.861 | 1.516553 | 7.89E-09 | 7.07E-07 |
| ENSRNOG00000060144 | *Ankle2* | 0.813 | -0.29919 | 0.001461 | 0.025325 |
| ENSRNOG00000060185 | *LOC103690317* | 1.599 | 0.677573 | 8.59E-12 | 1.18E-09 |
| ENSRNOG00000060594 | *Atn1* | 0.517 | -0.95301 | 3.58E-51 | 3.75E-48 |
| ENSRNOG00000061080 | *AABR07059168.2* | 0.824 | -0.27872 | 8.82E-07 | 5.17E-05 |
| ENSRNOG00000061348 | *Fam53b* | 1.57 | 0.650356 | 7.04E-09 | 6.34E-07 |
| ENSRNOG00000061376 | *Psca* | 1.225 | 0.29298 | 0.000887 | 0.017866 |
| ENSRNOG00000061862 | *Zbtb10* | 0.696 | -0.52225 | 0.000638 | 0.014049 |

**Table S4**. Differentially expressed genes between CCFM10 group and the model group of A7R5 cells

| Gene_id | Gene name | FC | Log2FC | Pvalue | Padjust |
| --- | --- | --- | --- | --- | --- |
| ENSRNOG00000000065 | *Pde6b* | 3.023 | 1.596128 | 0.00201 | 0.039228 |
| ENSRNOG00000000327 | *Hace1* | 1.261 | 0.334009 | 0.002121 | 0.040489 |
| ENSRNOG00000000397 | *Ccar1* | 0.824 | -0.27877 | 2.84E-05 | 0.001295 |
| ENSRNOG00000000413 | *Pln* | 1.673 | 0.742827 | 1.6E-05 | 0.000766 |
| ENSRNOG00000000500 | *Scube3* | 2.351 | 1.233558 | 0.000103 | 0.003924 |
| ENSRNOG00000001047 | *Map2k7* | 0.71 | -0.49511 | 0.000902 | 0.022456 |
| ENSRNOG00000001193 | *Hsf2bp* | 0.168 | -2.5738 | 0.000627 | 0.016986 |
| ENSRNOG00000001214 | *Pfkl* | 0.732 | -0.44951 | 1.05E-18 | 2.26E-16 |
| ENSRNOG00000001232 | *Slc19a1* | 1.248 | 0.31961 | 0.002177 | 0.041221 |
| ENSRNOG00000001273 | *Psmg3* | 0.767 | -0.38193 | 0.001106 | 0.025936 |
| ENSRNOG00000001294 | *Ift81* | 1.22 | 0.287209 | 0.001539 | 0.033074 |
| ENSRNOG00000001323 | *Zfp157* | 1.351 | 0.433955 | 0.00049 | 0.013757 |
| ENSRNOG00000001699 | *Setd4* | 0.744 | -0.42741 | 1.05E-05 | 0.00053 |
| ENSRNOG00000001720 | *Hes1* | 1.624 | 0.699375 | 1.37E-75 | 2.52E-72 |
| ENSRNOG00000001746 | *Ncbp2* | 1.217 | 0.283182 | 4.98E-05 | 0.00213 |
| ENSRNOG00000001996 | *Ythdc1* | 1.235 | 0.304563 | 2.19E-06 | 0.000132 |
| ENSRNOG00000002215 | *Mylk* | 1.218 | 0.285042 | 0.001601 | 0.033617 |
| ENSRNOG00000002332 | *Mospd1* | 0.756 | -0.40441 | 2.69E-05 | 0.001239 |
| ENSRNOG00000002537 | *Wnk3* | 1.284 | 0.360264 | 0.002349 | 0.043388 |
| ENSRNOG00000002667 | *Lamc2* | 1.224 | 0.291551 | 1.35E-10 | 1.49E-08 |
| ENSRNOG00000002831 | *Wfikkn2* | 2.447 | 1.291273 | 0.000294 | 0.009211 |
| ENSRNOG00000003088 | *Arhgap31* | 0.712 | -0.49049 | 0.000194 | 0.006686 |
| ENSRNOG00000003224 | *Nudt16l1* | 0.43 | -1.21841 | 2.25E-05 | 0.001057 |
| ENSRNOG00000003717 | *Cnih4* | 1.239 | 0.309143 | 1.4E-05 | 0.000688 |
| ENSRNOG00000003833 | *Nenf* | 1.444 | 0.529598 | 1.84E-15 | 3.06E-13 |
| ENSRNOG00000004035 | *Krr1* | 1.363 | 0.446962 | 0.000676 | 0.017992 |
| ENSRNOG00000004206 | *Glrx5* | 1.376 | 0.460878 | 9.87E-13 | 1.31E-10 |
| ENSRNOG00000004417 | *Fam117a* | 1.321 | 0.401566 | 0.000848 | 0.021447 |
| ENSRNOG00000004424 | *Rabif* | 1.248 | 0.319874 | 0.000342 | 0.010446 |
| ENSRNOG00000004488 | *Bdkrb1* | 2.338 | 1.22529 | 0.000211 | 0.007169 |
| ENSRNOG00000004757 | *Tmem158* | 1.864 | 0.898601 | 1.15E-26 | 4.16E-24 |
| ENSRNOG00000004874 | *Flrt3* | 0.798 | -0.32468 | 0.000326 | 0.009998 |
| ENSRNOG00000004940 | *Rnf215* | 1.237 | 0.306621 | 0.000674 | 0.017994 |
| ENSRNOG00000005099 | *Top3a* | 1.381 | 0.465544 | 0.00037 | 0.0112 |
| ENSRNOG00000005243 | *Pop1* | 0.621 | -0.68833 | 3.8E-05 | 0.001673 |
| ENSRNOG00000005303 | *Tbc1d25* | 0.785 | -0.34983 | 0.002179 | 0.041162 |
| ENSRNOG00000005515 | *Rhbdl3* | 1.292 | 0.369852 | 0.000199 | 0.006835 |
| ENSRNOG00000005542 | *Apob* | 1.327 | 0.407871 | 0.000224 | 0.007497 |
| ENSRNOG00000005608 | *Tead4* | 1.212 | 0.277232 | 0.001416 | 0.031195 |
| ENSRNOG00000005769 | *Smg8* | 0.812 | -0.30023 | 0.001122 | 0.026109 |
| ENSRNOG00000006460 | *Amdhd2* | 0.8 | -0.32235 | 0.000193 | 0.006675 |
| ENSRNOG00000006622 | *Cry1* | 1.478 | 0.563338 | 5.04E-05 | 0.002147 |
| ENSRNOG00000006700 | *Wdyhv1* | 0.456 | -1.13322 | 2.98E-05 | 0.001353 |
| ENSRNOG00000006740 | *Castor1* | 0.522 | -0.93761 | 0.000382 | 0.0114 |
| ENSRNOG00000006911 | *Sptb* | 1.249 | 0.320794 | 5.67E-05 | 0.002353 |
| ENSRNOG00000006950 | *Padi3* | 1.601 | 0.67859 | 0.002077 | 0.040115 |
| ENSRNOG00000007291 | *Terf1* | 1.656 | 0.727373 | 0.002524 | 0.045971 |
| ENSRNOG00000007319 | *Trib3* | 0.704 | -0.5058 | 2.48E-07 | 1.78E-05 |
| ENSRNOG00000007324 | *Plxna2* | 4.199 | 2.070059 | 0.000384 | 0.011434 |
| ENSRNOG00000007346 | *Grasp* | 1.257 | 0.330331 | 1.53E-07 | 1.12E-05 |
| ENSRNOG00000007437 | *Irf5* | 0.369 | -1.43682 | 0.002636 | 0.047435 |
| ENSRNOG00000007514 | *Sox12* | 2.339 | 1.225822 | 3.64E-24 | 1.12E-21 |
| ENSRNOG00000007590 | *Eya1* | 0.806 | -0.31076 | 0.000816 | 0.020806 |
| ENSRNOG00000007657 | *Col27a1* | 1.425 | 0.510519 | 0.000515 | 0.014335 |
| ENSRNOG00000007663 | *Rps6* | 0.515 | -0.95667 | 2.46E-09 | 2.27E-07 |
| ENSRNOG00000007823 | *Hoxb6* | 1.342 | 0.424264 | 0.000486 | 0.013743 |
| ENSRNOG00000008010 | *Hoxb5* | 0.795 | -0.33147 | 0.00039 | 0.011534 |
| ENSRNOG00000008075 | *Ift74* | 0.632 | -0.66223 | 8.55E-05 | 0.003316 |
| ENSRNOG00000008182 | *Htra3* | 1.395 | 0.480651 | 0.001414 | 0.031182 |
| ENSRNOG00000008277 | *Bag1* | 1.251 | 0.323279 | 8.57E-06 | 0.000443 |
| ENSRNOG00000008351 | *RGD1308117* | 2.346 | 1.229932 | 1.15E-05 | 0.000576 |
| ENSRNOG00000008452 | *Eid1* | 1.223 | 0.290044 | 1.64E-21 | 4.48E-19 |
| ENSRNOG00000008459 | *Anapc13* | 0.722 | -0.47037 | 0.001578 | 0.033377 |
| ENSRNOG00000008658 | *Mitf* | 0.603 | -0.7303 | 0.001129 | 0.026231 |
| ENSRNOG00000008873 | *Ino80b* | 1.365 | 0.449239 | 0.002164 | 0.041029 |
| ENSRNOG00000009173 | *Smad6* | 1.271 | 0.34576 | 1.9E-05 | 0.000907 |
| ENSRNOG00000009329 | *Nr1d1* | 0.795 | -0.33111 | 2.46E-06 | 0.000147 |
| ENSRNOG00000009481 | *Ddhd1* | 1.246 | 0.316974 | 0.000662 | 0.017791 |
| ENSRNOG00000009535 | *Stoml2* | 0.817 | -0.29149 | 0.002385 | 0.043919 |
| ENSRNOG00000010160 | *Ice2* | 1.337 | 0.418935 | 0.001879 | 0.037558 |
| ENSRNOG00000010268 | *Vom2r44* | 0.385 | -1.37586 | 0.001988 | 0.039028 |
| ENSRNOG00000010720 | *Mast4* | 0.824 | -0.2786 | 0.001671 | 0.034689 |
| ENSRNOG00000010875 | *Fam241a* | 1.764 | 0.81852 | 4.22E-14 | 6.56E-12 |
| ENSRNOG00000010894 | *Tmem203* | 0.699 | -0.51677 | 0.000273 | 0.008624 |
| ENSRNOG00000010932 | *Matn1* | 7.054 | 2.81852 | 0.001484 | 0.03222 |
| ENSRNOG00000011078 | *Srm* | 2.864 | 1.517853 | 1.73E-21 | 4.64E-19 |
| ENSRNOG00000011239 | *RGD1307595* | 0.768 | -0.38115 | 0.00049 | 0.013758 |
| ENSRNOG00000011284 | *Sgms2* | 1.205 | 0.268573 | 0.002815 | 0.049903 |
| ENSRNOG00000011305 | *Sox10* | 11.085 | 3.470597 | 0.001949 | 0.038491 |
| ENSRNOG00000011351 | *Mat1a* | 1.224 | 0.291902 | 9.52E-05 | 0.003644 |
| ENSRNOG00000011404 | *Chkb* | 1.311 | 0.390911 | 0.001686 | 0.034906 |
| ENSRNOG00000011421 | *Smap2* | 1.232 | 0.300789 | 2.13E-07 | 1.54E-05 |
| ENSRNOG00000011543 | *Fam118b* | 1.323 | 0.40408 | 8.22E-05 | 0.003205 |
| ENSRNOG00000011552 | *Mon1b* | 0.123 | -3.0273 | 1.26E-20 | 3.21E-18 |
| ENSRNOG00000011621 | *Hnrnpc* | 1.372 | 0.456627 | 5.84E-09 | 5.11E-07 |
| ENSRNOG00000011692 | *Dkk1* | 14.655 | 3.873286 | 1.04E-73 | 1.73E-70 |
| ENSRNOG00000012086 | *AABR07026271.1* | 1.753 | 0.809912 | 3.91E-06 | 0.000223 |
| ENSRNOG00000012091 | *Ppa2* | 0.601 | -0.73358 | 0.000433 | 0.012467 |
| ENSRNOG00000012106 | *Dnaja4* | 1.322 | 0.402553 | 0.001107 | 0.025897 |
| ENSRNOG00000012287 | *Slc35e1* | 5.383 | 2.428495 | 5.31E-08 | 4.12E-06 |
| ENSRNOG00000012349 | *Tlnrd1* | 1.265 | 0.339173 | 1.35E-08 | 1.13E-06 |
| ENSRNOG00000012785 | *Armc10* | 0.797 | -0.3265 | 0.000898 | 0.022438 |
| ENSRNOG00000013112 | *Dusp13* | 40.331 | 5.333815 | 3.33E-06 | 0.000193 |
| ENSRNOG00000013250 | *Pdcd5* | 2.17 | 1.117604 | 9.27E-32 | 4.16E-29 |
| ENSRNOG00000013328 | *Rbpms* | 1.428 | 0.513666 | 3.36E-05 | 0.001505 |
| ENSRNOG00000013459 | *Ints9* | 0.738 | -0.4377 | 0.000706 | 0.018546 |
| ENSRNOG00000013532 | *Pgam2* | 1.204 | 0.267956 | 0.000809 | 0.020667 |
| ENSRNOG00000013572 | *Lxn* | 0.812 | -0.3003 | 0.001553 | 0.033227 |
| ENSRNOG00000013663 | *Tmem86a* | 0.592 | -0.75702 | 0.000697 | 0.01839 |
| ENSRNOG00000013694 | *Ntng2* | 1.242 | 0.312821 | 0.000808 | 0.020677 |
| ENSRNOG00000013720 | *Aebp1* | 1.228 | 0.296455 | 9.45E-05 | 0.003634 |
| ENSRNOG00000013932 | *Rbp2* | 0.084 | -3.5738 | 0.001114 | 0.026011 |
| ENSRNOG00000013993 | *AABR07007000.1* | 1.408 | 0.493442 | 0.002618 | 0.047253 |
| ENSRNOG00000014019 | *Tbc1d7* | 0.764 | -0.38858 | 0.000159 | 0.005665 |
| ENSRNOG00000014089 | *Map3k2* | 0.78 | -0.35841 | 0.001934 | 0.038243 |
| ENSRNOG00000014161 | *Rbm15b* | 1.214 | 0.279728 | 9.54E-05 | 0.003641 |
| ENSRNOG00000014480 | *Sys1* | 1.245 | 0.316169 | 0.000439 | 0.012614 |
| ENSRNOG00000014551 | *Ccnj* | 157.837 | 7.30229 | 3.46E-19 | 7.87E-17 |
| ENSRNOG00000014588 | *Dbr1* | 0.79 | -0.34031 | 0.001545 | 0.033144 |
| ENSRNOG00000014684 | *Npr1* | 1.468 | 0.554064 | 0.001312 | 0.029523 |
| ENSRNOG00000014691 | *Ric3* | 0.751 | -0.41276 | 1.21E-05 | 0.000603 |
| ENSRNOG00000014765 | *Vapa* | 1.355 | 0.437827 | 1.99E-20 | 4.92E-18 |
| ENSRNOG00000014768 | *Glis3* | 1.231 | 0.300162 | 0.000454 | 0.013011 |
| ENSRNOG00000014795 | *Nr2f1* | 0.781 | -0.35604 | 0.002004 | 0.039155 |
| ENSRNOG00000015225 | *Gramd2b* | 0.827 | -0.27387 | 0.001631 | 0.034068 |
| ENSRNOG00000015275 | *Ska1* | 0.662 | -0.59456 | 0.001906 | 0.037916 |
| ENSRNOG00000015290 | *Tpi1* | 0.815 | -0.29571 | 9.44E-30 | 4.02E-27 |
| ENSRNOG00000015529 | *Cdca3* | 0.744 | -0.42662 | 2.76E-07 | 1.95E-05 |
| ENSRNOG00000015614 | *Ppp1r16b* | 5.375 | 2.426203 | 0.001748 | 0.035793 |
| ENSRNOG00000015750 | *Wnt7b* | 1.406 | 0.491971 | 0.002415 | 0.044363 |
| ENSRNOG00000015913 | *Tspan5* | 1.22 | 0.286864 | 3.76E-06 | 0.000215 |
| ENSRNOG00000016399 | *RGD1359127* | 0.815 | -0.29556 | 0.000526 | 0.014621 |
| ENSRNOG00000016405 | *Pcsk4* | 1.321 | 0.401566 | 0.000848 | 0.021447 |
| ENSRNOG00000016454 | *Nasp* | 0.809 | -0.30569 | 0.001568 | 0.033301 |
| ENSRNOG00000016622 | *Ankra2* | 0.666 | -0.58699 | 0.000176 | 0.006192 |
| ENSRNOG00000016879 | *Ldlrad4* | 1.307 | 0.386715 | 0.001082 | 0.025607 |
| ENSRNOG00000016940 | *Ppp2r2d* | 1.229 | 0.297048 | 0.000146 | 0.005263 |
| ENSRNOG00000017137 | *Eef1akmt2* | 0.697 | -0.52071 | 3.03E-09 | 2.78E-07 |
| ENSRNOG00000017226 | *Slc2a4* | 1.522 | 0.606324 | 0.001402 | 0.03096 |
| ENSRNOG00000017680 | *Dennd2d* | 12.093 | 3.596128 | 0.001036 | 0.02494 |
| ENSRNOG00000018230 | *Gtf2h2* | 0.688 | -0.53985 | 0.000945 | 0.02329 |
| ENSRNOG00000018379 | *Zfp688* | 0.745 | -0.42493 | 0.00053 | 0.014659 |
| ENSRNOG00000018877 | *Zfp689* | 1.286 | 0.363243 | 0.000684 | 0.018098 |
| ENSRNOG00000018886 | *Prxl2c* | 1.26 | 0.333915 | 0.001184 | 0.027206 |
| ENSRNOG00000018898 | *Mpi* | 0.808 | -0.30746 | 0.000871 | 0.021894 |
| ENSRNOG00000019027 | *Habp4* | 1.267 | 0.341917 | 0.001354 | 0.030219 |
| ENSRNOG00000019330 | *Procr* | 1.282 | 0.357847 | 1.82E-08 | 1.51E-06 |
| ENSRNOG00000019351 | *Chid1* | 0.012 | -6.44103 | 8.58E-12 | 1.06E-09 |
| ENSRNOG00000019381 | *Eri3* | 0.811 | -0.30207 | 0.00074 | 0.019231 |
| ENSRNOG00000019659 | *Aspa* | 4.233 | 2.081555 | 0.001091 | 0.025745 |
| ENSRNOG00000019698 | *Ssbp4* | 1.698 | 0.764073 | 0.001716 | 0.035395 |
| ENSRNOG00000019736 | *Nfs1* | 147.074 | 7.200397 | 4.19E-18 | 8.8E-16 |
| ENSRNOG00000019802 | *Zfp428* | 0.791 | -0.33747 | 0.002817 | 0.049825 |
| ENSRNOG00000020066 | *Pcdhb11* | 0.164 | -2.60786 | 0.000491 | 0.013761 |
| ENSRNOG00000020133 | *LOC108348044* | 0.004 | -7.83307 | 4.11E-26 | 1.39E-23 |
| ENSRNOG00000020150 | *Il18bp* | 0.142 | -2.81823 | 1.18E-06 | 7.51E-05 |
| ENSRNOG00000020263 | *Atp1a3* | 3.135 | 1.648595 | 0.001285 | 0.029144 |
| ENSRNOG00000020774 | *LOC103690163* | 0.697 | -0.52066 | 5.54E-05 | 0.002323 |
| ENSRNOG00000020848 | *Coq8b* | 0.823 | -0.28053 | 0.000777 | 0.020075 |
| ENSRNOG00000020952 | *Cgn* | 1.497 | 0.581773 | 0.001559 | 0.033275 |
| ENSRNOG00000020996 | *Vps51* | 0.809 | -0.30612 | 1.39E-05 | 0.000683 |
| ENSRNOG00000021013 | *Stx3* | 0.824 | -0.2785 | 3.83E-05 | 0.001682 |
| ENSRNOG00000021061 | *Map4k2* | 0.647 | -0.62843 | 0.000796 | 0.020475 |
| ENSRNOG00000021174 | *Macrod1* | 2.987 | 1.57885 | 7.75E-08 | 5.82E-06 |
| ENSRNOG00000021295 | *Rae1* | 1.202 | 0.265322 | 0.000105 | 0.003956 |
| ENSRNOG00000021463 | *Ppara* | 1.278 | 0.353558 | 0.002091 | 0.040337 |
| ENSRNOG00000021812 | *Scx* | 2.868 | 1.520179 | 0.000484 | 0.013747 |
| ENSRNOG00000022082 | *Dusp11* | 0.025 | -5.31386 | 2.62E-10 | 2.75E-08 |
| ENSRNOG00000022704 | *Esyt3* | 1.843 | 0.882432 | 0.001279 | 0.029049 |
| ENSRNOG00000022745 | *RGD1306502* | 0.772 | -0.37241 | 0.001291 | 0.029209 |
| ENSRNOG00000022777 | *Six1* | 1.22 | 0.287451 | 0.00038 | 0.011361 |
| ENSRNOG00000023352 | *Fam78a* | 1.263 | 0.336936 | 0.002474 | 0.045202 |
| ENSRNOG00000023712 | *Stox1* | 3.527 | 1.81852 | 0.002824 | 0.049849 |
| ENSRNOG00000024889 | *Snip1* | 1.934 | 0.951613 | 5.24E-05 | 0.00221 |
| ENSRNOG00000025059 | *Nxph4* | 1.904 | 0.928703 | 0.001366 | 0.030406 |
| ENSRNOG00000025269 | *Slc25a44* | 0.767 | -0.38223 | 0.000341 | 0.010424 |
| ENSRNOG00000025406 | *Iqgap2* | 1.346 | 0.428151 | 0.002154 | 0.040879 |
| ENSRNOG00000026672 | *MGC94199* | 2.902 | 1.5372 | 9.64E-14 | 1.43E-11 |
| ENSRNOG00000026748 | *Dennd2a* | 1.234 | 0.303494 | 6.62E-05 | 0.002642 |
| ENSRNOG00000027456 | *Cdc42bpg* | 1.408 | 0.493829 | 8.2E-05 | 0.003203 |
| ENSRNOG00000027593 | *Ndufv3* | 2.31 | 1.20806 | 0.000957 | 0.023552 |
| ENSRNOG00000027628 | *Elp5* | 1.252 | 0.324125 | 0.001577 | 0.033413 |
| ENSRNOG00000027646 | *Pop4* | 0.8 | -0.32239 | 0.001845 | 0.037091 |
| ENSRNOG00000027839 | *Ptk2b* | 1.349 | 0.432009 | 0.000335 | 0.010258 |
| ENSRNOG00000028063 | *Tmem38b* | 1.421 | 0.506693 | 0.002799 | 0.049657 |
| ENSRNOG00000028302 | *Smarcb1* | 1.676 | 0.745355 | 4.31E-16 | 7.61E-14 |
| ENSRNOG00000028350 | *Arse* | 0.82 | -0.28663 | 0.00014 | 0.005039 |
| ENSRNOG00000029047 | *Cubn* | 2.207 | 1.14241 | 0.001799 | 0.036653 |
| ENSRNOG00000029079 | *Hspb7* | 1.207 | 0.271251 | 0.000379 | 0.011373 |
| ENSRNOG00000030750 | *Zfp2* | 0.101 | -3.31076 | 4.49E-05 | 0.001956 |
| ENSRNOG00000030932 | *Zfp14* | 3.286 | 1.716385 | 3.29E-07 | 2.31E-05 |
| ENSRNOG00000031660 | *LOC500948* | 10.366 | 3.373773 | 7.4E-12 | 9.25E-10 |
| ENSRNOG00000031731 | *Fam216a* | 0.813 | -0.29827 | 3.53E-05 | 0.001565 |
| ENSRNOG00000032136 | *Cdc42ep3* | 1.248 | 0.319863 | 0.000282 | 0.008877 |
| ENSRNOG00000032439 | *Rsl1d1* | 8.734 | 3.126661 | 4.91E-48 | 3.4E-45 |
| ENSRNOG00000032618 | *Mst1r* | 13.101 | 3.711605 | 0.000552 | 0.015196 |
| ENSRNOG00000032902 | *Ybx1-ps3* | 2.85 | 1.510821 | 6.38E-72 | 9.64E-69 |
| ENSRNOG00000036572 | *Sfxn4* | 0.699 | -0.51585 | 0.001798 | 0.036678 |
| ENSRNOG00000037850 | *Marc2* | 1.288 | 0.364699 | 2.95E-06 | 0.000172 |
| ENSRNOG00000037984 | *Sccpdh* | 0.001 | -9.62034 | 4.65E-70 | 5.94E-67 |
| ENSRNOG00000038166 | *Ptgr2* | 1.344 | 0.426385 | 0.002709 | 0.048436 |
| ENSRNOG00000039476 | *Pcdhb2* | 1.398 | 0.48347 | 1.11E-07 | 8.14E-06 |
| ENSRNOG00000039876 | *LOC681410* | 1.273 | 0.348678 | 2.73E-24 | 8.57E-22 |
| ENSRNOG00000042916 | *Rwdd1* | 1.278 | 0.354412 | 0.001192 | 0.027354 |
| ENSRNOG00000043486 | *Tnfrsf26* | 1.293 | 0.37064 | 0.000651 | 0.017541 |
| ENSRNOG00000045548 | *Entpd7* | 0.006 | -7.37237 | 4.12E-20 | 1.01E-17 |
| ENSRNOG00000045670 | *Tnfsf12* | 1.334 | 0.415419 | 2.2E-06 | 0.000132 |
| ENSRNOG00000045721 | *Exoc2* | 0.635 | -0.65548 | 0.00142 | 0.031234 |
| ENSRNOG00000045814 | *RGD1563962* | 0.633 | -0.65967 | 6.3E-05 | 0.002542 |
| ENSRNOG00000045911 | *Mepce* | 0.008 | -6.89597 | 2.56E-15 | 4.21E-13 |
| ENSRNOG00000046192 | *Nsun4* | 0.02 | -5.60942 | 2.62E-07 | 1.87E-05 |
| ENSRNOG00000046414 | *Vwa5a* | 0.483 | -1.05014 | 6.45E-44 | 3.97E-41 |
| ENSRNOG00000046452 | *Fcgr2b* | 7.39 | 2.885635 | 5.55E-05 | 0.002321 |
| ENSRNOG00000046990 | *Cpne1* | 0.705 | -0.50439 | 8.8E-09 | 7.57E-07 |
| ENSRNOG00000047113 | *Spsb2* | 2.425 | 1.278241 | 8.81E-05 | 0.003412 |
| ENSRNOG00000047115 | *Cox16* | 0.735 | -0.44411 | 0.000463 | 0.013222 |
| ENSRNOG00000047396 | *Rmnd5b* | 0.731 | -0.45156 | 0.000226 | 0.007531 |
| ENSRNOG00000047854 | *RT1-DMa* | 22.171 | 4.470597 | 0.000792 | 0.020392 |
| ENSRNOG00000047971 | *Mrpl36* | 20.7 | 4.37153 | 0.001258 | 0.028661 |
| ENSRNOG00000047982 | *LOC501317* | 0.449 | -1.15576 | 0.001552 | 0.03326 |
| ENSRNOG00000048169 | *Tuba8* | 26.202 | 4.711605 | 0.000227 | 0.007543 |
| ENSRNOG00000048172 | *Rac3* | 0.322 | -1.63269 | 0.002618 | 0.047158 |
| ENSRNOG00000048195 | *Nudt13* | 0.389 | -1.3608 | 0.000487 | 0.013743 |
| ENSRNOG00000048365 | *Eno2* | 0.708 | -0.49734 | 4.72E-08 | 3.7E-06 |
| ENSRNOG00000048441 | *Wdr82* | 1.291 | 0.36859 | 4.53E-07 | 3.13E-05 |
| ENSRNOG00000048470 | *Pomp* | 2.543 | 1.346257 | 1.22E-06 | 7.72E-05 |
| ENSRNOG00000048553 | *Iqcc* | 19.531 | 4.287662 | 0.001822 | 0.036896 |
| ENSRNOG00000048561 | *Hprt1* | 1.604 | 0.681945 | 0.001972 | 0.038809 |
| ENSRNOG00000048650 | *LOC103690175* | 4.498 | 2.169259 | 5.45E-16 | 9.42E-14 |
| ENSRNOG00000048932 | *Smagp* | 1.473 | 0.558435 | 1.37E-05 | 0.000677 |
| ENSRNOG00000049097 | *Rpl7a* | 1.547 | 0.629785 | 6.3E-17 | 1.2E-14 |
| ENSRNOG00000049287 | *MGC94199* | 0.281 | -1.83038 | 1.12E-11 | 1.38E-09 |
| ENSRNOG00000049593 | *Wbp11* | 0.688 | -0.53913 | 3.24E-11 | 3.95E-09 |
| ENSRNOG00000050374 | *Pigg* | 232.254 | 7.85956 | 5.85E-76 | 1.21E-72 |
| ENSRNOG00000050430 | *Vav1* | 0.68 | -0.55652 | 0.001447 | 0.031578 |
| ENSRNOG00000050792 | *Tnfaip6* | 1.283 | 0.360061 | 0.000113 | 0.004234 |
| ENSRNOG00000050827 | *NEWGENE_1310561* | 0.484 | -1.04767 | 2.35E-31 | 1.03E-28 |
| ENSRNOG00000050869 | *Cebpd* | 3.401 | 1.766053 | 0.000905 | 0.022509 |
| ENSRNOG00000051927 | *RF00607* | 30.556 | 4.933363 | 6.02E-05 | 0.002456 |
| ENSRNOG00000052199 | *RGD1566138* | 67.621 | 6.079406 | 1.64E-09 | 1.53E-07 |
| ENSRNOG00000052247 | *Manba* | 1.264 | 0.338006 | 8.48E-06 | 0.00044 |
| ENSRNOG00000052613 | *Casp6* | 2.274 | 1.185295 | 0.000174 | 0.00616 |
| ENSRNOG00000052804 | *Hoxa5* | 36.824 | 5.20257 | 9.29E-06 | 0.000479 |
| ENSRNOG00000052837 | *Dcun1d2* | 0.333 | -1.58849 | 1.92E-06 | 0.000117 |
| ENSRNOG00000053541 | *Kansl2* | 0.782 | -0.35537 | 6.57E-06 | 0.00035 |
| ENSRNOG00000053735 | *Hebp2* | 1.239 | 0.308846 | 0.000479 | 0.013653 |
| ENSRNOG00000054334 | *Abhd10* | 0.503 | -0.99158 | 0.001167 | 0.02684 |
| ENSRNOG00000054563 | *Selenoh* | 0.788 | -0.34434 | 5.89E-06 | 0.000316 |
| ENSRNOG00000054581 | *Tmem101* | 1.218 | 0.284542 | 0.000116 | 0.004308 |
| ENSRNOG00000056701 | *Gtf2a2* | 0.032 | -4.94675 | 5.17E-05 | 0.002183 |
| ENSRNOG00000056740 | *Cylc1* | 0.396 | -1.33607 | 0.001003 | 0.024401 |
| ENSRNOG00000057009 | *Ptp4a1* | 63.913 | 5.998032 | 4.46E-09 | 4E-07 |
| ENSRNOG00000057031 | *Six3* | 11.085 | 3.470597 | 0.001949 | 0.038491 |
| ENSRNOG00000057347 | *Cebpb* | 2.731 | 1.44949 | 1.26E-82 | 4.2E-79 |
| ENSRNOG00000057696 | *Sbk1* | 3.149 | 1.654825 | 8.77E-16 | 1.47E-13 |
| ENSRNOG00000057795 | *Dusp13* | 0.023 | -5.47104 | 9.45E-07 | 6.06E-05 |
| ENSRNOG00000057880 | *Myh11* | 1.371 | 0.454711 | 2.66E-10 | 2.78E-08 |
| ENSRNOG00000058439 | *Fam50a* | 8.193 | 3.034308 | 1.52E-80 | 3.61E-77 |
| ENSRNOG00000058555 | *RF00100* | 0.028 | -5.15876 | 1.19E-05 | 0.000595 |
| ENSRNOG00000058834 | *LOC103692471* | 3.628 | 1.859162 | 0.000181 | 0.006332 |
| ENSRNOG00000059069 | *AABR07061378.1* | 0.005 | -7.76901 | 3.48E-25 | 1.11E-22 |
| ENSRNOG00000059091 | *Potem* | 5.375 | 2.426203 | 0.001748 | 0.035793 |
| ENSRNOG00000059166 | *Ldb3* | 1.241 | 0.311305 | 0.000514 | 0.014322 |
| ENSRNOG00000059276 | *LOC498265* | 0.639 | -0.64722 | 0.001198 | 0.027449 |
| ENSRNOG00000059386 | *Nudt19* | 0.796 | -0.32942 | 0.002565 | 0.04656 |
| ENSRNOG00000059862 | *Avpr2* | 0.22 | -2.18148 | 3.53E-05 | 0.001566 |
| ENSRNOG00000060185 | *LOC103690317* | 1.56 | 0.641992 | 3.47E-10 | 3.56E-08 |
| ENSRNOG00000060594 | *Atn1* | 0.624 | -0.68063 | 3.97E-28 | 1.61E-25 |
| ENSRNOG00000060604 | *LOC103694910* | 0.033 | -4.90722 | 6.66E-05 | 0.002651 |
| ENSRNOG00000061215 | *Crym* | 0.586 | -0.77124 | 0.002206 | 0.041578 |
| ENSRNOG00000061348 | *Fam53b* | 1.921 | 0.941638 | 7.56E-18 | 1.49E-15 |
| ENSRNOG00000061989 | *Nkrf* | 2.526 | 1.337106 | 0.001046 | 0.024993 |
| ENSRNOG00000062125 | *Aox3* | 1.737 | 0.796979 | 7.78E-07 | 5.03E-05 |


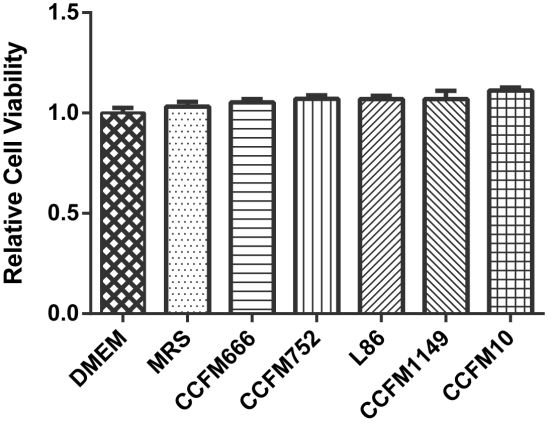


**Figure S1** Influence of MRS broth and probiotic supernatents preincubation on cell viability of A7R5 cells. Values are mean ± SEM (n=5).


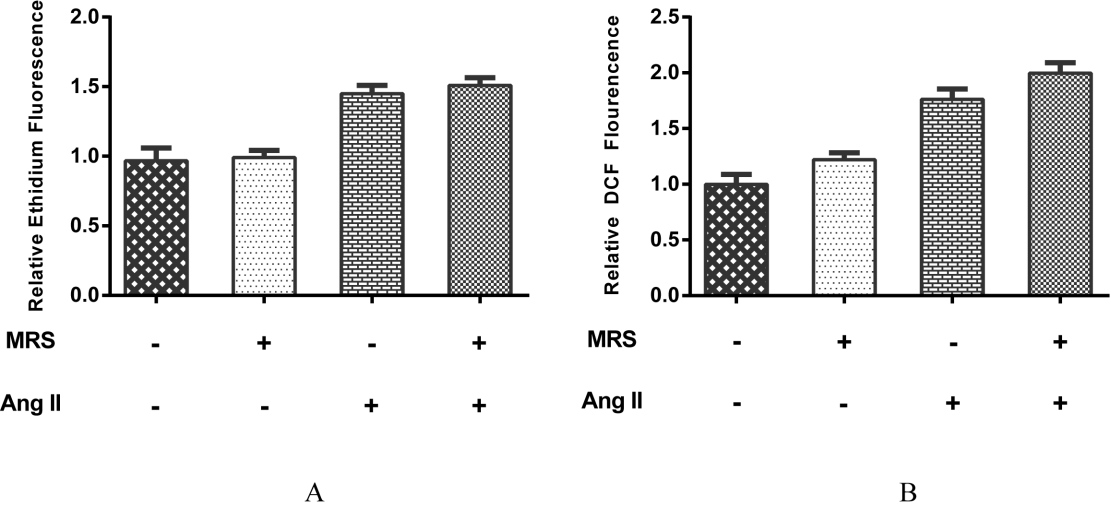


**Figure S2** Influence of MRS broth preincubation and angiotensin II stimulation on intracellular ROS levels of A7R5 cells. (A) Influence of MRS broth and angiotensin II on intracellular superoxide anion (O_2_^·-^) level. (B) Influence of MRS broth and angiotensin II on intracellular hydrogen peroxide (H_2_O_2_) level of A7R5 cells. Relative intracellular O_2_^·-^  and H_2_O_2_ levels were determined using DHE and DCFH-DA, respectively. Values are mean ± SEM (n=3).


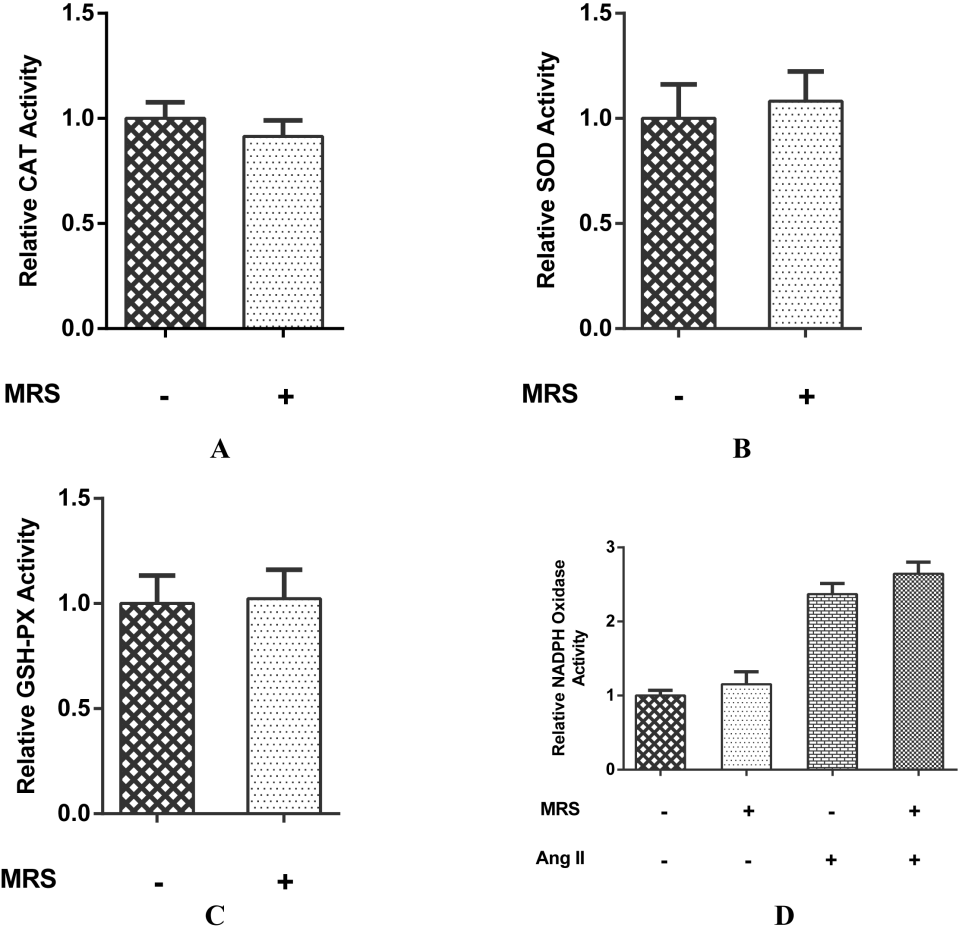


**Figure S3** Influence of MRS broth and angiotensin II on intracellular enzyme activity in A7R5 cells. Influence of MRS broth on intracellular CAT activity (A), total SOD (T-SOD) activity (B), and GSH-PX activity (C) in A7R5; and the effects of MRS broth preincubation and angiotensin II stimulation on NADPH oxidase activation in A7R5 (D). Values are mean ± SEM (n=3).


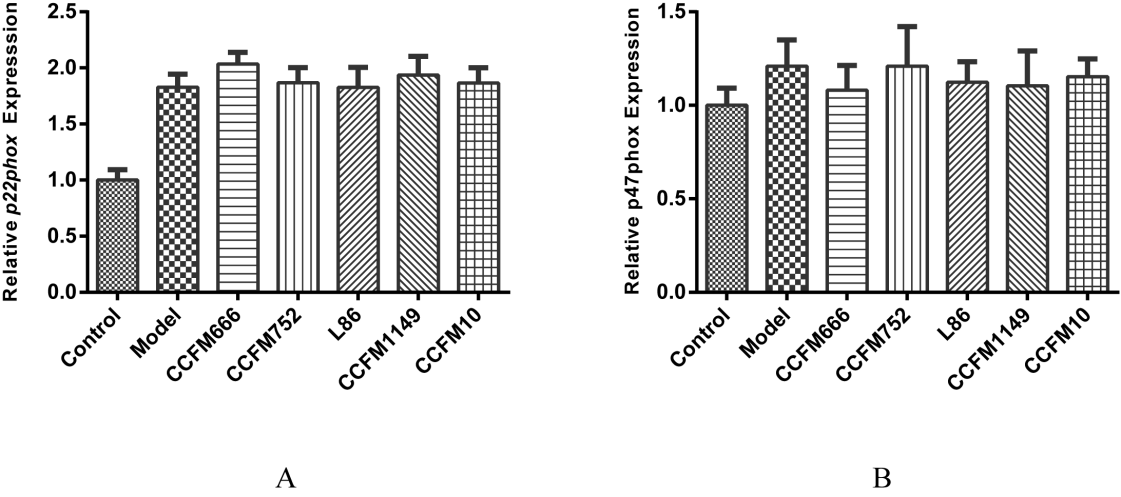


**Figure S4** Relative transcriptional levels of *p22phox* (A) and *p47phox* (B) of A7R5 cells. Values are mean ± SEM (n=3).
