# Supplementary figures and images for: Supernatants of Bifidobacterium longum and Lactobacillus plantarum Strains Exhibited Antioxidative Effects on A7R5 Cells
Source: Microorganisms. 2021 Feb 22;9(2):452. doi: 10.3390/microorganisms9020452 (PMC7927071; doi:10.3390/microorganisms9020452)

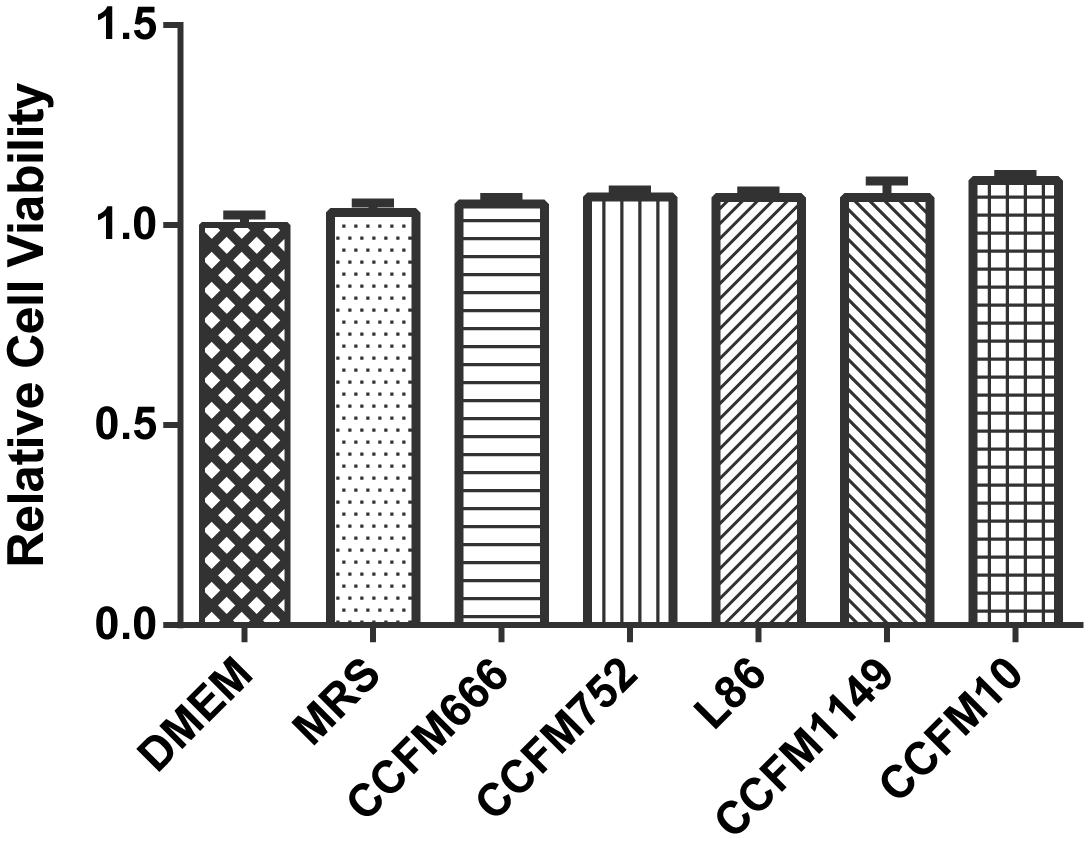

Supplement: Supplementary file 1 [file microorganisms-09-00452-s001.zip › Supplementary Information/Figure S1.tif]

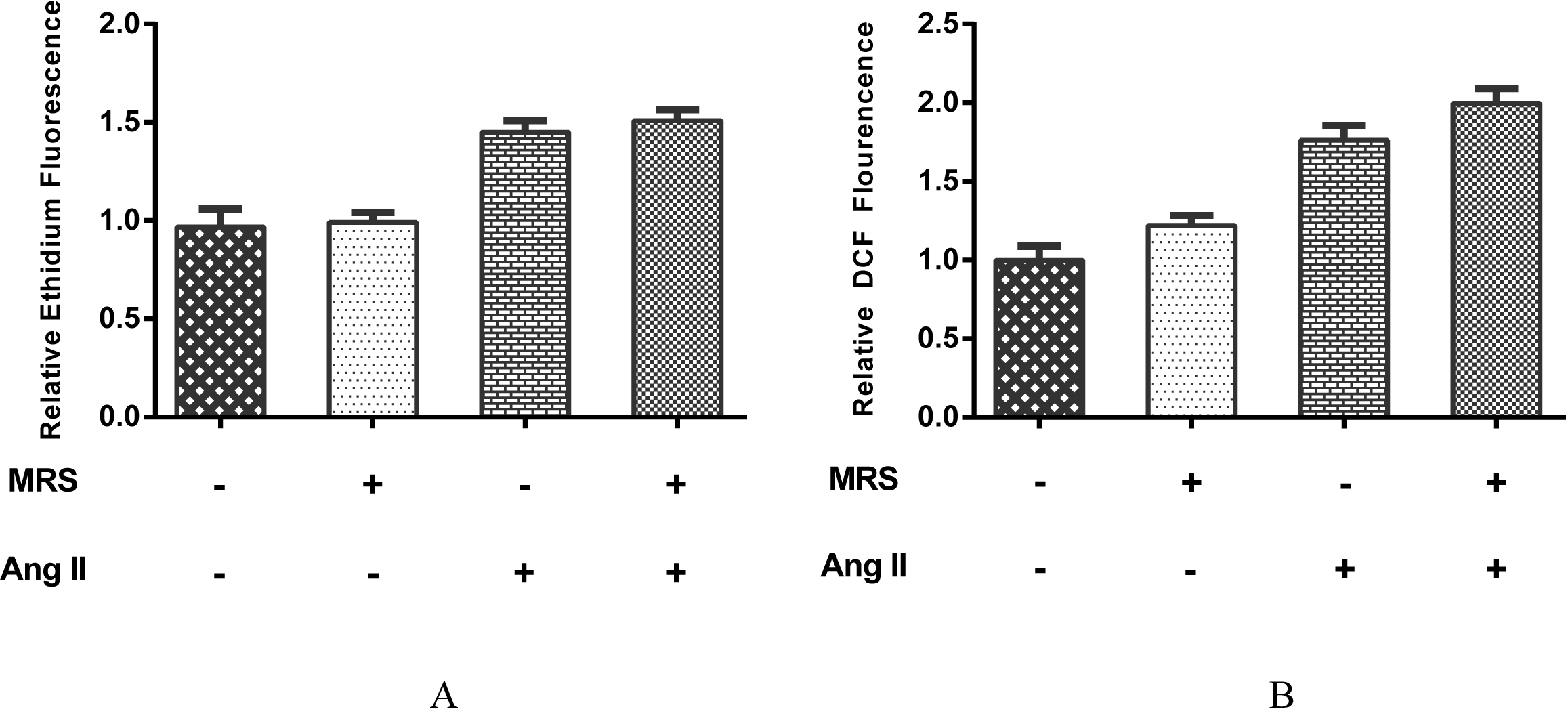

Supplement: Supplementary file 1 [file microorganisms-09-00452-s001.zip › Supplementary Information/Figure S2.tif]

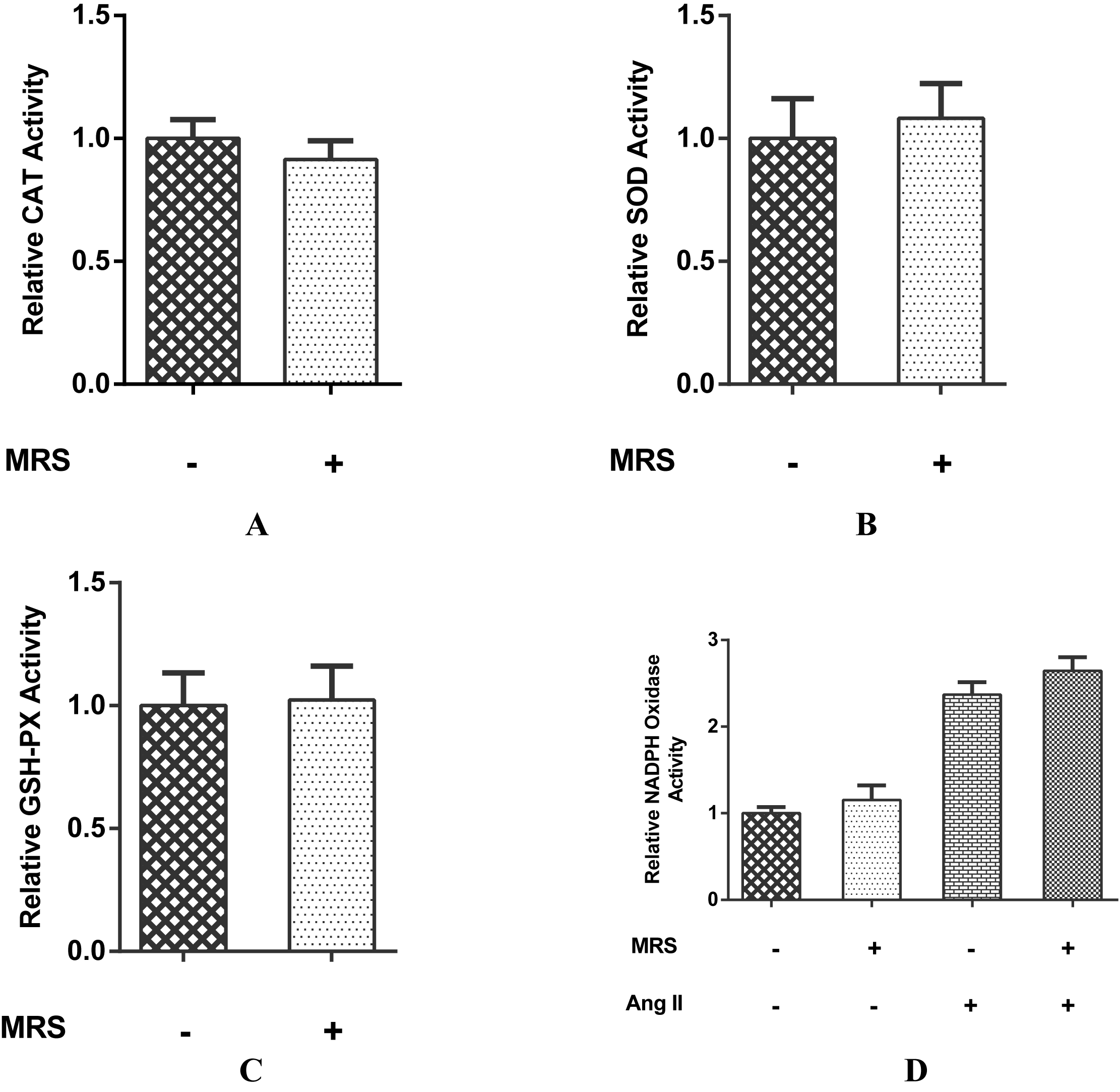

Supplement: Supplementary file 1 [file microorganisms-09-00452-s001.zip › Supplementary Information/Figure S3.tif]

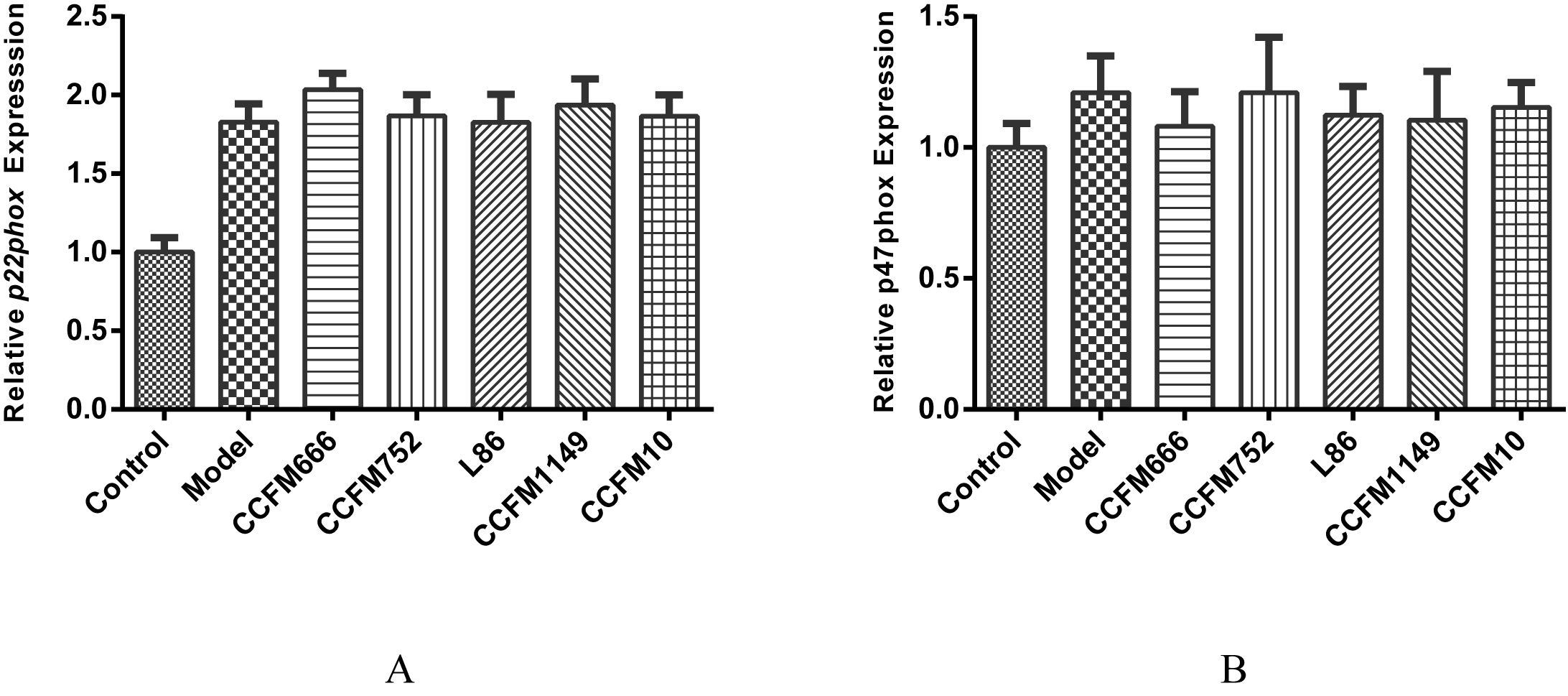

Supplement: Supplementary file 1 [file microorganisms-09-00452-s001.zip › Supplementary Information/Figure S4.tif]
